# Ferrocenyl‐Pyrenes, Ferrocenyl‐9,10‐Phenanthrenediones, and Ferrocenyl‐9,10‐Dimethoxyphenanthrenes: Charge‐Transfer Studies and SWCNT Functionalization†

**DOI:** 10.1002/chem.201904450

**Published:** 2020-01-23

**Authors:** Andrea Preuß, Sebastian Notz, Eduard Kovalski, Marcus Korb, Thomas Blaudeck, Xiao Hu, Jörg Schuster, Dominique Miesel, Tobias Rüffer, Alexander Hildebrandt, Katja Schreiter, Stefan Spange, Stefan E. Schulz, Heinrich Lang

**Affiliations:** ^1^ Faculty of Natural Sciences Institute of Chemistry Inorganic Chemistry Technische Universität Chemnitz 09107 Chemnitz Germany; ^2^ Current address: Faculty of Science School of Molecular Sciences The University of Western Australia Crawley, Perth WA 6009 Australia; ^3^ Center for Microtechnologies (ZfM) Technische Universität Chemnitz 09107 Chemnitz Germany; ^4^ Fraunhofer Institute for Electronic Nano Systems (ENAS) Technologie-Campus 3 09126 Chemnitz Germany; ^5^ Center for Advancing Electronics Dresden (cfaed) 09107 Chemnitz Germany; ^6^ Faculty of Natural Sciences Institute of Chemistry, Polymer Chemistry Technische Universität Chemnitz 09107 Chemnitz Germany

**Keywords:** (spectro)electrochemistry, density functional calculations, ferrocene, pyrene, solid-state structures, solvatochromism

## Abstract

The synthesis of 1‐Fc‐ (**3**), 1‐Br‐6‐Fc‐ (**5 a**), 2‐Br‐7‐Fc‐ (**7 a**), 1,6‐Fc_2_‐ (**5 b**), 2,7‐Fc_2_‐pyrene (**7 b**), 3,6‐Fc_2_‐9,10‐phenanthrenedione (**10**), and 3,6‐Fc_2_‐9,10‐dimethoxyphenanthrene (**12**; Fc=Fe(η^5^‐C_5_H_4_)(η^5^‐C_5_H_5_)) is discussed. Of these compounds, **10** and **12** form 1D or 2D coordination polymers in the solid state. (Spectro)Electrochemical studies confirmed reversible Fc/Fc^+^ redox events between −130 and 160 mV. 1,6‐ and 2,7‐Substitution in **5 a** (*E*°′=−130 mV) and **7 a** (*E*°′=50 mV) influences the redox potentials, whereas the ones of **5 b** and **7 b** (*E*°′=20 mV) are independent. Compounds **5 b**, **7 b**, **10**, and **12** show single Fc oxidation processes with redox splittings between 70 and 100 mV. UV/Vis/NIR spectroelectrochemistry confirmed a weak electron transfer between Fe^II^/Fe^III^ in mixed‐valent [**5 b**]^+^ and [**12**]^+^. DFT calculations showed that **5 b** *non*‐covalently interacts with the single‐walled carbon nanotube (SWCNT) sidewalls as proven by, for example, disentangling experiments. In addition, CV studies of the as‐obtained dispersions confirmed exohedral attachment of **5 b** at the SWCNTs.

## Graphical abstract

**Ferrocene complexes**: The synthesis of 1‐Fc‐, 1‐Br‐6‐Fc‐, 2‐Br‐7‐Fc‐, 1,6‐Fc_2_‐, 2,7‐Fc_2_‐pyrene, 3,6‐Fc_2_‐9,10‐phenanthrenedione, and 3,6‐Fc_2_‐9,10‐dimethoxyphenanthrene (Fc=Fe(η^5^‐C_5_H_4_)(η^5^‐C_5_H_5_)) is discussed. Two of these compounds form 1D or 2D coordination polymers in the solid state. (Spectro)electrochemical studies confirmed reversible Fc/Fc^+^ redox events between −130 and 160 mV.

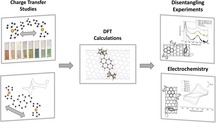

## Introduction

Redox‐active ferrocenyl‐functionalized five‐1 and six‐membered^2^ heterocycles as well as aromatic^2^, ^3^, ^4^, ^5^ and polyaromatic hydrocarbons^6^, ^7^, ^8^, ^9^ are well suited as model compounds for studying intramolecular electron transfer processes, as they possess short electron transfer distances. They are easy to functionalize at the heterocyclic or aromatic hydrocarbon core and/or the redox‐active moiety, enabling the straightforward modification of the electronic properties. In this respect, we recently focused on the synthesis and (spectro)electrochemical behavior of, in particular, ferrocenyl‐substituted heterocycles featuring SiR_2_,^10^ NR,^11^, ^12^ PR,^12^, ^13^, ^14^ O,^15^, ^16^ S,^17^, ^18^ ‐^*c*^C_2_N_2_S,^19^ TiCp_2_,^20^ or ZrCp_2_ 21 (R=H, organic group; Cp=η^5^‐C_5_H_5_) constituents. With the example of 2,5‐Fc_2_‐^*c*^C_4_H_2_E (Fc=Fe(η^5^‐C_5_H_4_)(η^5^‐C_5_H_5_); E=O, S, NR), it could be shown that similar geometries and hence comparable electrostatic interactions enable a correlation between electrochemical and spectroscopic properties.^11^, ^15^

Furthermore, aromatics are suitable to modify carbon nanotubes (CNTs) by *non*‐covalent interactions, for example, π–π stacking.^22^, ^23^ In this respect, naphthalene, phenanthrene, or pyrene derivatives are promising candidates to be attached to the CNT π perimeter through non‐covalent bonding.^24^ The pyrene group is favored over smaller polycyclic hydrocarbons, like phenyl‐based compounds, as reported for polycyclic aromatic ammonium salts.^25^, ^26^ The interactions between pyrenes and SWCNT (single‐walled carbon nanotube) sidewalls could also be proven by molecular dynamics simulations and DFT calculations.^27^, ^28^ Blaudeck et al. reported SWCNTs that are surface‐functionalized with 12‐(pyren‐1‐yl)dodecane‐1‐thiol, which form hybrids with gold nanoparticles.^29^ Such arrangements allow, for example, tuning of the transistor channel in nanoelectronic SWCNT field‐effect transistors, which enables the fabrication of optoplasmonic sensor arrays on the chip level.^29^, ^30^

Ferrocene‐functionalized CNT nanohybrids have attracted much attention in the fields of (bio)analytical electrochemistry,^31^ solar energy conversion,^32^ or as electrocatalytically active materials^33^ and photosensitizers,^34^ whereby the modification can be achieved by exohedral functionalization at the CNTs surface^31^, ^35^, ^36^ or in an endohedral fashion by filling the nanotubes with ferrocene species.^37^, ^38^, ^39^, ^40^ Exohedral covalent attachment of ferrocenes to SWCNTs is thereby better considered as non‐covalent binding. For instance, Allali et al. reported the linkage of diverse (poly)ethylene glycol‐functionalized ferrocenes to CNTs and their successful use as an electrochemical biosensor for the redox couple NADH/NAD^+^ (NADH=dihydronicotinamide adenine dinucleotide).^31^, ^36^ In contrast, Singh et al. prepared adenine‐functionalized multi‐walled carbon nanotubes, whereby the nucleobase pairing caused the uracil‐ferrocenyl attachment.^35^ The ferrocenyl electrochemical properties were retained after immobilization at the CNT surface, which makes the supramolecular hybrid material interesting for application in photovoltaic or optoelectronics.^35^

Non‐covalently functionalized CNTs bearing a ferrocenyl substituent were recently obtained through π‐stacking of 3‐ferrocenyl‐*N*‐(pyren‐1‐ylmethyl)propanamide to SWCNTs and tested as glucose sensor electrode materials.^41^ Ding and co‐workers functionalized CNTs with {[(2,2′‐bipyridyl)2‐(4,4′‐bis(4‐pyrenyl‐1‐ylbutyloxy)‐2,2′‐bipyridyl)]ruthenium(II)}(PF_6_)_2_.^42^ In the pyrene‐Ru/SWCNTs/Pt modified electrodes, the Ru^II^/Ru^III^ redox potential is stable over 200 cycles. Another hybrid material is CNT/cobalt bis(4‐pyren‐1‐yl‐*N*‐[5‐([2,2′;6′,2′′]terpyridin‐4′‐yloxy)‐pentyl]butyramide).^43^ However, all of these materials possess an alkyl linker between the pyrene entity and the transition metal complex fragment.

Lately, we became interested in attaching redox‐active Fc‐functionalized polyaromatic hydrocarbons to SWCNTs as a way to achieve debundeling as well as to influence the electronic properties of the thus‐modified carbon nanotubes and to use them as field‐effect sensors.^44^

Hence, within this study, the synthesis and characterization of a series of 1‐Fc‐, 1,6‐Fc_2_‐, and 2,7‐Fc_2_‐pyrenes, 3,6‐Fc_2_‐9,10‐phenanthrenedione, and 3,6‐Fc_2_‐9,10‐dimethoxyphenanthrene compounds is discussed. Their (spectro)electrochemical properties and their molecular structures in the solid state are reported. DFT calculations for 1,6‐Fc_2_‐pyrene and semiconducting and metallic SWCNTs are provided, as well as the use of 1,6‐Fc_2_‐pyrene to disentangle SWCNTs. The electrochemical properties of the as‐obtained dispersions are presented.

## Results and Discussion

**Scheme 1:**
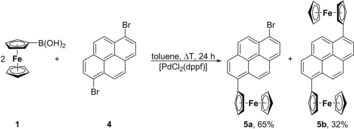
Synthesis of **5 a**,**b** by treatment of **1** with **4**.

**Scheme 2:**
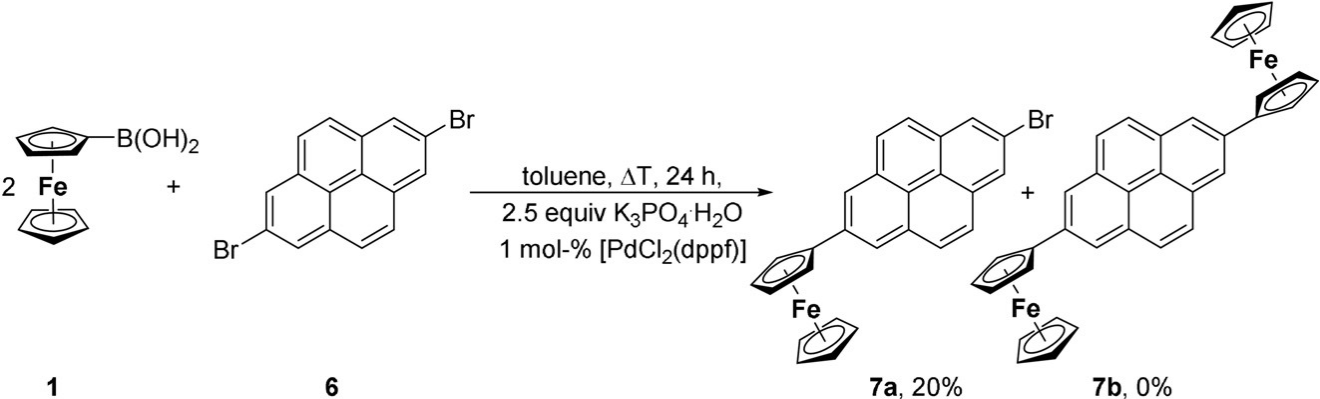
Synthesis of **7 a** from **1** and **6**.

**Scheme 3:**
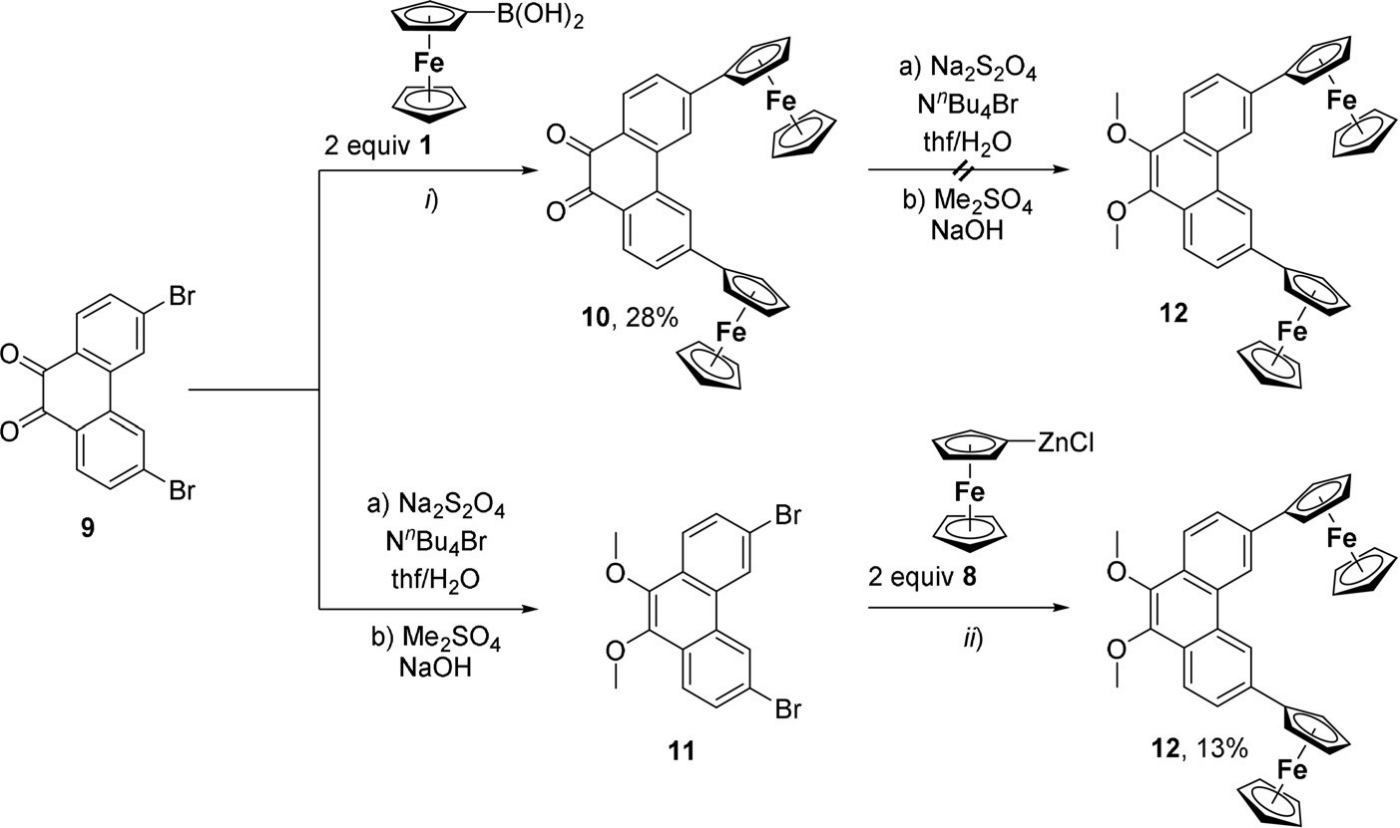
Synthesis protocol for the preparation of **10**–**12**. i) Toluene, Δ*T*, 24 h, 1 mol % [PdCl_2_(dppf)], 2.5 equiv K_3_PO_4_ **⋅**H_2_O; ii) tetrahydrofuran, Δ*T*, 24 h, 1 mol % [PdCl_2_(dppf)].

For the synthesis of the title compounds, either Suzuki–Miyaura (synthesis of **3**, **5 a**,**b**, **7 a**, **10**) or Negishi (**7 b**, **12**) C−C cross‐coupling reactions were applied (reactions 1 and (2), Schemes 1, 2, 3).
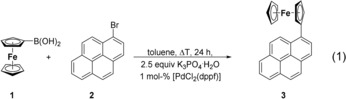



Ferrocene boronic acid FcB(OH)_2_ (**1**; Fc=Fe(η^5^‐C_5_H_4_)(η^5^‐C_5_H_5_)) was treated with the respective 1‐Br‐ (**2**), 1,6‐Br_2_‐ (**4**), 2,7‐Br_2_‐pyrene (**6**), or 3,6‐Br_2_‐9,10‐phenanthrenedione (**9**) in the presence of [PdCl_2_(dppf)] (dppf=1,1′‐bis(diphenylphosphino)ferrocene) as the catalyst in the molar ratio of 1:1 or 2:1 in boiling toluene. After appropriate work‐up, compounds **3**, **5 a**,**b**, **7 a**, and **10** could be isolated as orange (**3**, **5 a**,**b**, **7 a**) or green (**10**) solids in a yield of 3–65 % (Experimental Section). For comparison, compound 9‐ferrocenylphenanthren was synthesized according to the Suzuki C−C cross‐coupling protocol used within the synthesis of **3**, **5 a**,**b**, **7 a**, and **10** (for more details, see the Supporting Information, Reaction SI1).

In the transmetalation reaction of **1** with 1,6‐Br_2_‐C_16_H_8_ (**4**) in the molar ratio of 2:1, a mixture of the respective mono‐ and diferrocenyl‐functionalized pyrenes **5 a**,**b** was formed (Scheme 1). Even using an excess of **1** and/or higher reaction temperatures and prolonged stirring did not result in the formation of **5 b** as the main product. Compounds **5 a**,**b** could be separated from each other by column chromatography (Experimental Section).

In contrast, treatment of **1** with 2,7‐Br_2_‐C_16_H_8_ (**6**) in a 2:1 ratio in boiling toluene solely gave the monoferrocenyl‐substituted species 2‐Br‐7‐Fc‐pyrene (**7 a**), which was obtained as an orange solid in a yield of 32 % after column chromatography (Scheme 2). Even using a four‐fold excess of **1** did not give **7 b**, neither at ambient temperature nor in boiling toluene, and increasing the reaction time from 24 to 72 h was also unsuccessful. Changing the solvent from toluene to more polar ones and applying typical Suzuki reaction conditions did not significantly improve the yield of **7 a** nor the formation of **7 b**.

However, diferrocenyl pyrene **7 b** was accessible through the Negishi C−C cross‐coupling reaction as shown in reaction 2. Thus, treatment of two equivalents of FcZnCl (**8**)^45^ with **6** in the presence of 1.0 mol % [PdCl_2_(dppf)] as the catalyst in boiling tetrahydrofuran produced **7 b**, however, in a yield as low as 2 % (Experimental Section). In this reaction, other than **7 b**, ferrocene was formed as the main product. Compound **7 b** is a very poorly soluble species in common polar organic solvents. Once **7 b** was isolated in its solid form, it was almost impossible to redissolve it and hence spectroscopic and electrochemical experimental data on this organometallic compound are limited.
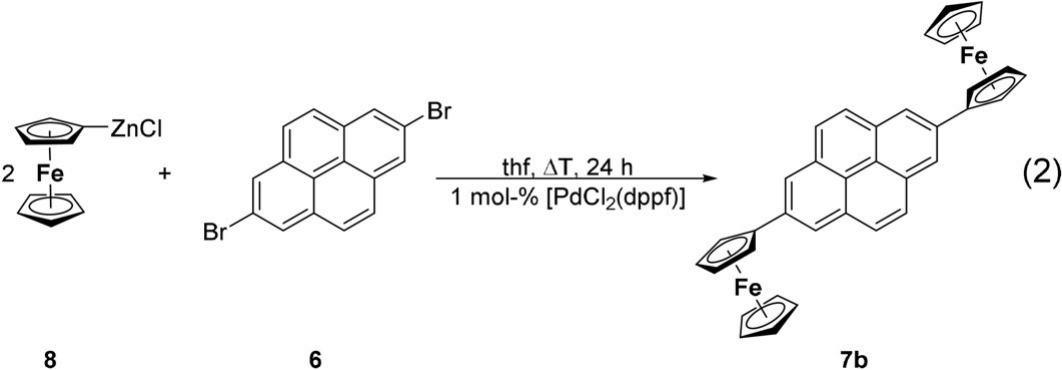



Compounds **10** and **12** were synthesized by either the Suzuki–Miyaura (**10**) or the Negishi (**12**) reaction starting from 3,6‐dibromophenanthrene‐9,10‐dione **9** (Scheme 3). Treatment of **9** with two equivalents of ferrocene boronic acid (**1**) in toluene in the presence of catalytic amounts of [PdCl_2_(dppf)] led to the formation of green 3,6‐diferrocenylphenanthrene‐9,10‐dione (**10**) in a yield of 28 %. However, the synthetic methodology described by Phulwale et al. for the reduction of the ketone functionalities in **10** did not lead to the formation of the respective 3,6‐diferrocenyl‐9,10‐dimethoxyphenanthrene compound **12**.^46^ Nevertheless, this compound was accessible when the reduction of **9** was performed prior to the C−C cross‐coupling reaction. Thus, compound **11** could be isolated in virtual quantitative yield. Upon treatment of **11** with two equivalents of FcZnCl (**8**) in tetrahydrofuran as solvent for 24 h in the presence of [PdCl_2_(dppf)] gave orange **12** in an overall yield of 13 % (Scheme 3, Experimental Section).

Compounds **3**, **5 a**,**b**, **7 a**,**b**, **10**, and **12** are orange solids with the exception of **10**, which is green. They are stable towards air and moisture in the solid state and in solution. With the exclusion of **7 b**, they dissolve in common polar organic solvents, such as dichloromethane, tetrahydrofuran, and acetonitrile; in toluene and hexane they are less soluble. Moreover, compound **10** shows solvatochromic behavior (see below).

The identity of **5 a**,**b**, **7 a**,**b**, **10**, and **12** was confirmed by elemental analysis, IR, ^1^H and ^13^C{^1^H} NMR spectroscopy, and high‐resolution ESI‐TOF mass‐spectrometry (Experimental Section), whereas the physical and analytical data (^1^H and ^13^C{^1^H} NMR, HRMS) of **3** are documented in reference 9. The solid‐state structures of all compounds (including 9‐Fc‐phenanthrene, for comparison; see the Supporting Information) were determined by single‐crystal X‐ray structure analysis. The electrochemical and spectroelectrochemical properties of all compounds were determined by cyclic and square wave voltammetry and in situ UV/Vis/NIR spectroscopy (except for **7 b**).

The spectroscopic (^1^H, ^13^C{^1^H} NMR) data of **3**, **5 a**,**b**, **7 a**,**b**, **10**, and **12** are consistent with their formulations as mono‐ or diferrocenyl‐functionalized pyrene, 9,10‐phenanthrenedione, and 9,10‐dimethoxyphenanthrene compounds, showing the characteristic coupling patterns of the appropriate isomers (Experimental Section).^9^ However, for **7 b** no reliable ^13^C{^1^H} NMR data could be obtained, owing to its low solubility in common polar organic solvents (see above).

**Figure 1:**
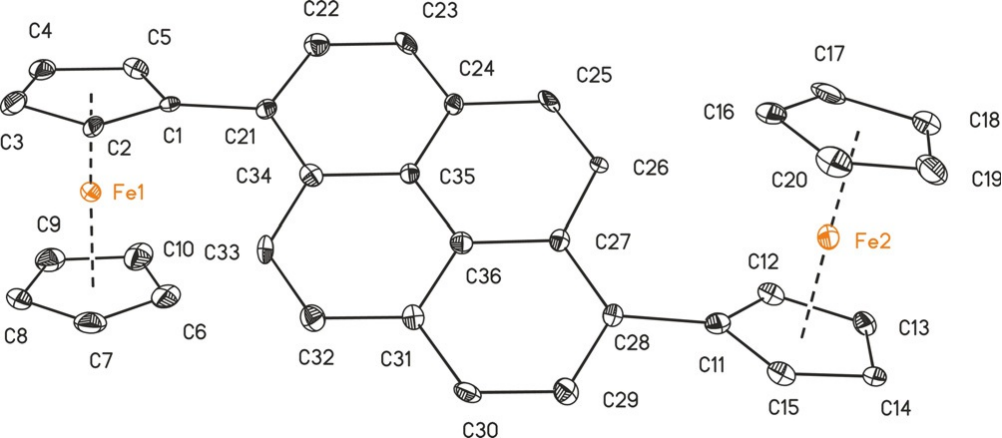
ORTEP (50 % probability level) of the molecular structure of **5 b** with the atom numbering scheme. All hydrogen atoms have been omitted for clarity. Selected bond lengths [Å] and angles [°]: C1−C21 1.480(12), C11−C28 1.504(12); C5‐C1‐C21 123.2(5), C2‐C1‐C21 129.5(8), C12‐C11‐C28 129.1(9), C15‐C11‐C28 124.3(9).

**Figure 2:**
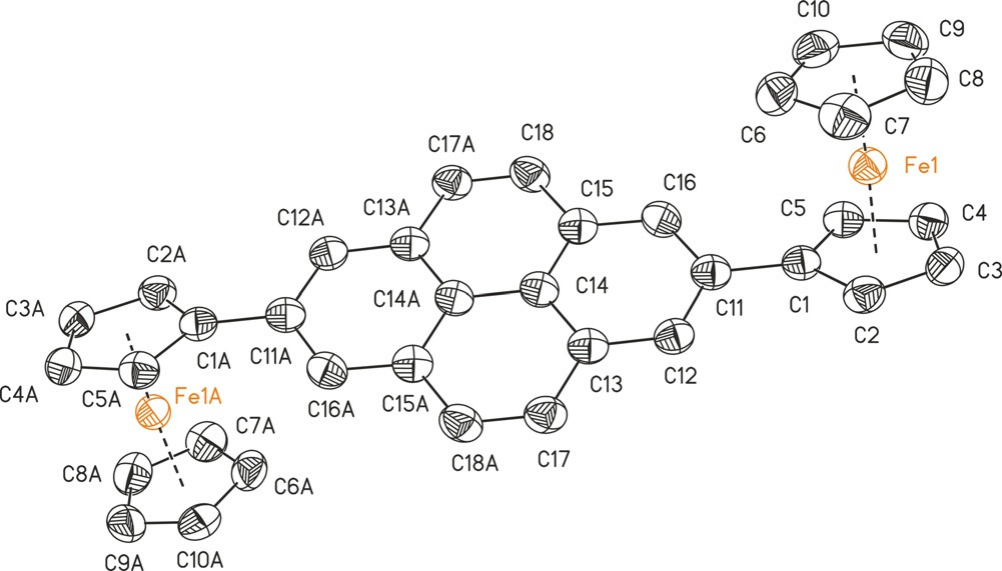
ORTEP (50 % probability level) of the molecular structure of **7 b** with the atom numbering scheme. All hydrogen atoms have been omitted for clarity. Selected bond lengths [Å] and angles [°], and torsion angles [°]: C1−C11 1.477(4); C5‐C1‐C11 125.8(2), C2‐C1‐C11 127.2(3), C12‐C11‐C1 120.6(2), C16‐C11‐C1 120.5(3); C11‐C1‐C2‐C3 −177.0(3), C11‐C1‐C5‐C4 177.5(2), C5‐C1‐C11‐C12 161.4(3), C2‐C1‐C11‐C12 −20.9(4), C5‐C1‐C11‐C16 −19.4(4), C2‐C1‐C11‐C16 158.3(3), C1‐C11‐C12‐C13 179.5(2), C1‐C11‐C16‐C15 −179.0(2). Symmetry generated atoms are indicated by the suffix A; symmetry code: −*x*+2, −*y*+1, −*z*+2.

**Figure 3:**
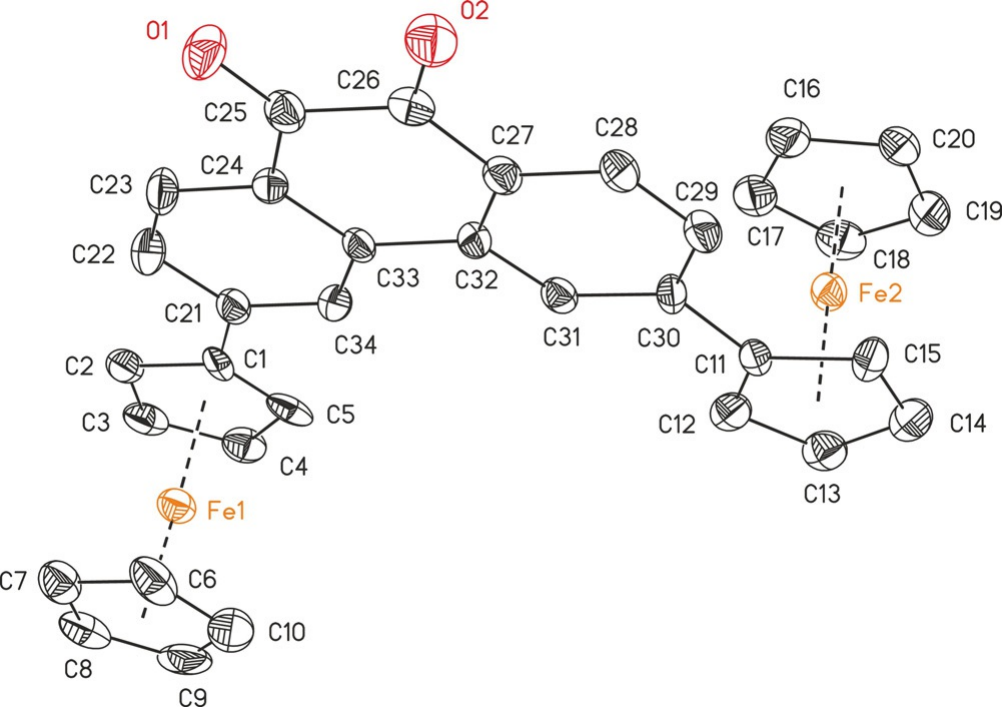
ORTEP (50 % probability level) of the molecular structure of **10** with the atom numbering scheme. All hydrogen atoms and solvent molecules have been omitted for clarity. Selected bond lengths [Å] and angles [°], and torsion angles [°]: C1−C21 1.554(8), C11−C30 1.542(7), C25−O1 1.204(6), C26−O2 1.220(6); C2‐C1‐C21 121.7(5), C5‐C1‐C21 125.1(5), C12‐C11‐C30 125.7(5), C15‐C11‐C30 122.4(5), O1‐C25‐C24 124.0(5), O1‐C25‐C26 118.7(5), O2‐C26‐C25 118.4(5), O2‐C26‐C27 123.4(5); C23‐C24‐C25‐O1 −0.5(8), O1‐C25‐C26‐C27 −179.1(5), O1‐C25‐C26‐O2 −178.9(5), C24‐C25‐C26‐O2 −178.9(5), O2‐C26‐C27‐C28 −0.1(8), O2‐C26‐C27‐C32 −179.7(5).

**Figure 4:**
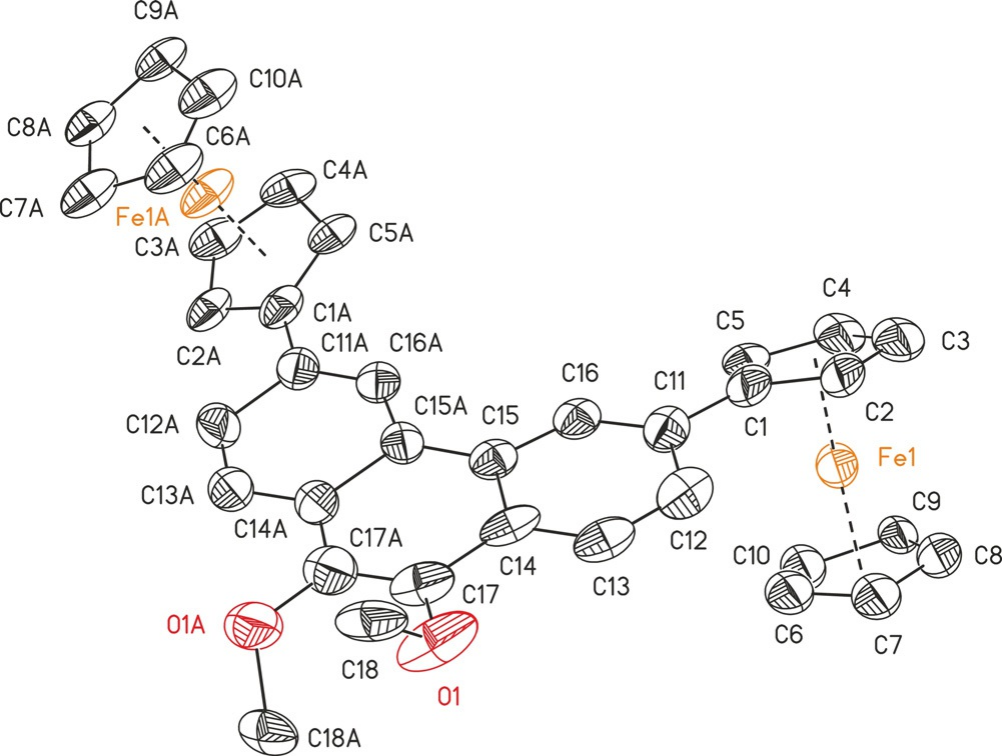
ORTEP (50 % probability level) of the molecular structure of **12** with the atom numbering scheme. All hydrogen atoms have been omitted for clarity. Selected bond lengths [Å] and angles [°], and torsion angles [°]: C1−C11 1.48(3), C17−O1 1.28(3), C18−O1 1.41(3); C17‐O1‐C18 116.1(19); C14‐C17‐O1‐C18 121(3), O1‐C17‐C17A‐O1A 13(5). Symmetry generated atoms are indicated by the suffix A; symmetry code: −*x*+1, −*y*, *z*.

The molecular structures of **3**, **5 a**,**b**, **7 a**,**b**, **10**, **12**, and 9‐ferrocenylphenanthrene in the solid state have been determined by single‐crystal X‐ray diffraction analysis. The ORTEP diagrams are shown in Figures 1, 2, 3, 4 and Figures SI1–SI4 (in the Supporting Information), and selected bond lengths [Å], angles [deg], and torsion angles [deg] are given in the captions of these figures. Additional crystallographic data are summarized in Tables SI1–SI5 (in the Supporting Information). Suitable single crystals were obtained either by diffusion of pentane into a chloroform solution containing **7 b** or by crystallization from saturated dichloromethane solutions containing **3**, **5 a**,**b**, **7 a**, or **12** at ambient temperature, whereas **9** was crystallized from a saturated hexane solution at −20 °C.

The compounds crystallize in the monoclinic space groups *C*2/*c* (**3**), *I*2/*a* (**5 a**), *P*2_1_/*n* (**5 b**), or *P*2_1_/*c* (**7 a**,**b**, **10**) and in the orthorhombic space groups *Pnn*2 (**12**) and *P*2_1_2_1_2_1_ (9‐ferrocenylphenanthrene) with one (**3**, **5 a**,**b**, **7 a**,**b**, **12**, 9‐ferrocenyl‐phenanthrene) or two (**10**) independent molecules in the asymmetric unit. Intermolecular π–π interactions were investigated between the aromatic entities and the ferrocenyl cyclopentadienyl units (Figures SI5–SI12, in the Supporting Information).

The distance between the ferrocenyl cyclopentadienyl *ipso*‐carbon atoms and the aromatic units is similar for all investigated compounds (from 1.476(3) Å (**7 a**) to 1.554(8) Å (**10**)) and agree well with literature values.^2^, ^6^, ^8^, ^15^, ^47^, ^48^, ^49^

In Tables SI3 and SI4 (in the Supporting Information), the bond lengths of the different aromatic cores are summarized. The substitution pattern does not affect the position of the single and double bonds in the aromatic moieties. This observation is in good agreement with values published for other substituted pyrenyl and phenanthrenyl derivatives.^9^, ^49^, ^50^, ^51^

The rotation of the ferrocenyls out of the aromatic co‐planarity was examined for **3**, **5 a**,**b**, **7 a**,**b**, **12**, and 9‐ferrocenylphenanthrene. Compounds containing hydrogen atoms in position 8 with regard to the Fc group (**3**, **5 a**,**b**, and 9‐FcPhen) possess increased values of 31.5(5)° (**5 b**) to 40.05(10)° (**5 a**), owing to a steric interaction with the α‐H atoms of the ferrocenyls. In contrast, the geometric properties of the 2‐ or 3‐substitution pattern (**7 a**,**b**, **10**, **12**) allow for more planar intersections between 0.1(3)° (**10**) and 22.1(9)° (**12**). Hence, these compounds should be preferred to achieve a high degree of electron transfer interaction between the π‐system of the ferrocenyls and the aromatic cores.^15^ The plane intersections of **3**, **5 a**,**b**, and 9‐ferrocenylphenanthrene argue for a weak interaction. In addition, the rms distortions (root mean square deviations) of the condensed aromatic units were calculated and are summarized in Table SI5 (in the Supporting Information), where **5 a** (0.0497) and **12** (0.0680) showed exceptional high values.

The diferrocenyl‐substituted compounds **5 b**, **7 b**, **10**, and **12** exhibit an *anti*‐positioning of the Fc groups towards each other with regard to the aromatic planes.^2^, ^10^, ^13^, ^14^, ^17^, ^18^, ^52^, ^53^ Furthermore, the Fe⋅⋅⋅Fe distances were calculated, ranging from 9.0374(13) (**10**) to 12.7288(8) Å (**7 b**). Thereby, the Fe⋅⋅⋅Fe distances in **10** (9.0374(13) Å Fe1–Fe2; 9.0390(13) Å, Fe3–Fe4) and **12** (10.123(6) Å) point towards a better electronic coupling between the ferrocenyl groups than in **5 b** (11.477(2) Å) and **7 b** (12.7288(8) Å).

With regard to the utilization of **3**, **5 a**,**b**, **7 a**,**b**, **10**, **12**, and 9‐ferrocenylphenanthrene as π‐surfactants on CNTs, the interaction through intermolecular π/C−H⋅⋅⋅π bonding was investigated for the aromatic and ferrocenyl units (Figures SI5–SI12 in the Supporting Information). Except for **7 b**, all compounds show intermolecular π–π interactions (for more details, see Figures SI5–SI12 in the Supporting Information), of which **3**, **5 b**, and 9‐ferrocenylphenanthrene show T‐shaped π‐interactions between C_5_H_5_/C_5_H_4_ units and the aromatic moieties with distances of 4.5276(14) (**3**)–4.994(6) Å (**5 b**). The solid‐state structures of **5 a** and **7 a** show T‐shaped and parallel displaced π‐interactions with distances between 4.6081(17) (**5 a**) and 4.6925(14) Å (**7 a**) and angles from 84.68(15) (**5 a**) to 89.24(14)° (**7 a**). The parallel displaced π‐interactions between the pyrene cores of **5 a** and **7 a** show distances of approximately 3.6 Å, forming dimers (for more details, see the Supporting Information).

In case of **10** and **12**, the parallel displaced π‐interactions between the arenes led to the formation of 1D (**12**) and 2D coordination polymers (**10**) along the *a*‐ (**10**) or *b*‐axis (**12**), resulting in the formation of columns (for more details, see Figures SI11 and SI12 in the Supporting Information). The columnar structure of **12** is based on the parallel displaced π‐interactions involving C11–C16 of the phenanthrene with centroid–centroid distances of 5.165(12) Å, exceeding the criterion for π‐stacking (3.3–3.8 Å).^54^, ^55^, ^56^ However, owing to displacement from ideal stacking, short distances between C13/C14/C16 and their symmetry equivalents of the adjacent molecule of 3.04(3)–3.39(3) Å were found, indicating strong π–π interactions.^54^, ^55^, ^56^

Compound **10** builds a 2D coordination polymer, whereby both molecules of the asymmetric unit are responsible for the columnar structure. Parallel displaced π‐interactions between the ferrocenyl's cyclopentadienyls interconnect the columns. The arrangement along the *a*‐axis is based on the interactions between the arenes of the phenanthrene backbone with distances ranging from 3.589(3) to 4.549(3) Å. Furthermore, this stacking is supported by π‐interactions involving the Fc‐C_5_H_4_ units and the aromatic moieties with distances ranging from 3.466(3) to 4.800(3) Å (for more details, see Figure SI11 in the Supporting Information).

### Solvatochromism

UV/Vis absorption spectra (*ṽ*_max_) of compound **10** were measured in a set of 23 common organic solvents of different polarities and hydrogen‐bonding abilities (Table 1). The concentration of **10** was *c*≈10^−5^ mol L^−1^. Concentration‐dependent UV/Vis spectroscopic investigations did not show a shift of the UV/Vis absorption maxima.

**Table 1:** UV/Vis absorption maxima (*ṽ*_max_) of **10** measured in 23 solvents of different polarity and hydrogen‐bond ability.

Solvent	*ṽ*_max_ [10^−3^ cm^−1^]	Solvent	*ṽ*_max_ [10^−3^ cm^−1^]
*N*,*N‐*dimethylformamide	16.42	toluene	17.51
dimethyl sulfoxide	16.92	2,2,2‐trifluoroethanol	15.20
tetramethylurea	17.48	tetrachloromethane	17.70
tetrahydrofuran	17.61	anisole	16.98
diethyl ether	17.73	benzene	17.30
1,1,1,3,3,3‐hexafluoro‐2‐propanol	15.20	4‐butyrolactone	16.78
1‐propanol	16.34	1,2‐dichloroethane	16.53
2‐propanol	16.34	nitromethane	16.67
ethanol	16.48	benzonitrile	16.53
methanol	16.61	cyclohexane	18.08
dichloromethane	16.50	hexane	17.76
ethyl acetate	17.51	Δ*ṽ* [cm^−1^]^[a]^	2880

[a] Solvatochromic range.

**Figure 5:**
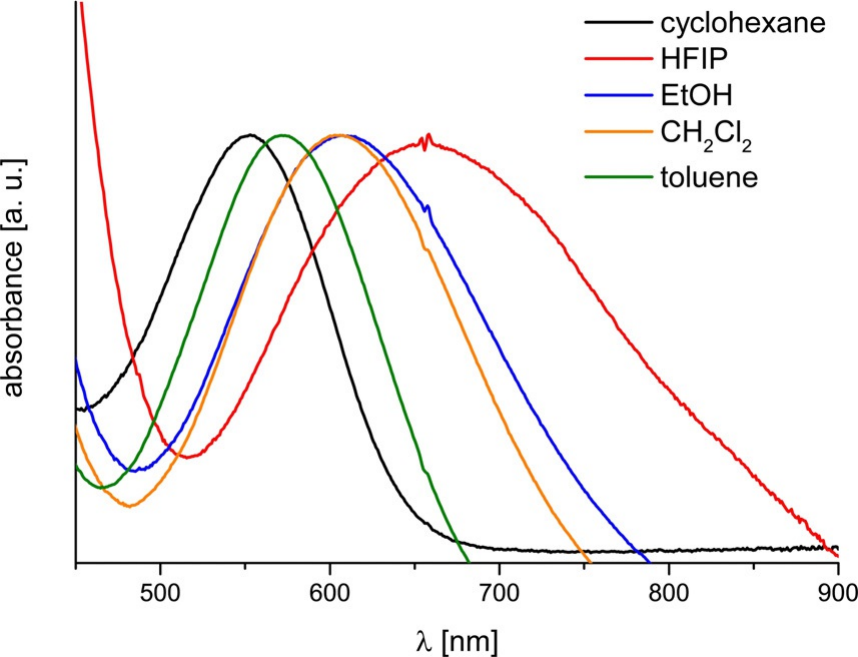
UV/Vis absorption spectra of **10** measured in five solvents (HFIP=1,1,1,3,3,3‐hexafluoro‐2‐propanol).

Compound **10** shows the largest bathochromic shift *λ*_max_ in 1,1,1,3,3,3‐hexafluoro‐2‐propanol and in 2,2,2‐trifluoroethanol with *λ*_max_ (**10**)=658 nm (Figure 5). The shortest *λ*_max_ was measured in cyclohexane (*λ*_max_ (**10**)=553 nm). This shift corresponds to a positive solvatochromism with a solvatochromic range of Δ*ṽ*_max_ (**10**)=2880 cm^−1^.

The interactions of solvatochromic dyes with pure solvents or solvent mixtures arise from a combination of many effects.^57^, ^58^, ^59^, ^60^, ^61^, ^62^ Multiple intermolecular solute/solvent interactions can be described by using linear free energy relationships (LSER).^57^, ^58^ To separate the effects of nonspecific chemical interactions, including electrostatic effects (dipolarity/polarizability), from specific interactions (hydrogen bonding), the simplified Kamlet–Taft equation [Eq. (1)] was applied.^63^, ^64^, ^65^, ^66^, ^67^

(1) $$\tilde{v}{}_{\max}=\tilde{v}{}_{\max,0}+a\alpha +b\beta +s\pi {}^{*}$$

According to Equation (1), the effect of hydrogen‐bonding donor capacity (HBD),^63^ the hydrogen‐bonding acceptor capacity (HBA),^64^ and the dipolarity/polarizability^65^, ^66^ of a solvent can be expressed by *α*, *β*, and π*. In addition, *ṽ*_max,0_ corresponds to a standard process, referenced to a nonpolar medium. The parameters *a*, *b*, and *s* represent solvent‐independent correlation coefficients, which reflect the relative effect of each of the three parameter *α*, *β*, and π*.

The solvent parameters *α*, *β*, and π* used for the multiple linear regression analysis are given in Table SI6 (in the Supporting Information).^60^ The regression of **10**, which is qualitatively the best according to the solvent scales of Kamlet–Taft, is given in Table 2.

**Table 2:** Solvent‐independent correlation coefficients *a*, *b*, and *s* of Kamlet–Taft parameters *α*, *β*, and π*, respectively, solute property of reference system (*ṽ*_max_), correlation coefficient (*R*), significance (*F*), and numbers of solvents (*n*) calculated for the solvatochromism of **10**.

	Compound **10**
*ṽ*_max,0_	(18.035±0.142)
*a*	(−1.098±0.106)
*b*	–
*s*	(−1.368±0.209)
*n*	23
*R*	0.938
*F*	<0.0001

The correlation coefficient *r* is greater than 0.90 for the LSE relationship, indicating a high validity of the multiparameter equation, allowing significant conclusions to be drawn. Compound **10** shows a positive solvatochromism with increasing acidity and dipolarity/polarizability of the solvents.

The negative sign of *s* indicates that the electronically excited state of **10** becomes solvated stronger and is consequently stabilized with increasing solvent dipolarity/polarizability. Owing to the strength of the higher dipole moment, the energy of the electronically excited state decreases more than the energy of the ground state. This is in good agreement with a bathochromic shift of the UV/Vis absorption maxima with increased polarity of the solvent.

The negative sign of parameter *a* for **10** indicates that there is a bathochromic shift of *λ*_max_ with increasing hydrogen‐bond donor capacity of the solvent (positive solvatochromism). Owing to the solvation of the carbonyl oxygen atom by HBD solvents the push–pull character becomes enhanced. This effect is on the same order of magnitude as the influence of the dipolarity/polarizability. The hydrogen‐bond acceptor capacity of the solvents, on the other hand, has no influence on the solvatochromic behavior of **10** (*b=*0).

A similar behavior was observed for ferrocenyl‐substituted maleimides.^68^ These compounds also show a positive solvatochromism, but a smaller solvatochromic range (Δ*ṽ*_max_) of 1820–2460 cm^−1^ in contrast to **10** with 2880 cm^−1^, which indicates a stronger solvatochromic behavior.^68^

### Electrochemistry and spectroelectrochemistry

The redox properties of compounds **3**, **5 a**,**b**, **7 a**,**b**, **10**, and **12** were investigated by cyclic voltammetry (CV), square wave voltammetry (SWV), and spectroelectrochemistry (in situ UV/Vis/NIR). As supporting electrolyte, an anhydrous dichloromethane solution containing 0.1 mol L^−1^ of [N*n*Bu_4_][B(C_6_F_5_)_4_] was used.^13^, ^69^, ^70^, ^71^, ^72^, ^73^ Contrary to smaller counter ions such as [PF_6_]^−^ or [Cl]^−^, [B(C_6_F_5_)_4_]^−^ stabilizes highly charged species in solution and minimizes ion pairing effects.^74^, ^75^, ^76^, ^77^ The shielding of the electrostatic interactions among the two redox sites is achieved by the ion pairing with the electrolyte's counter‐ion hence a minimization of this effect leads to an increase in the observed redox potential splitting.^78^ The voltammetry measurements were performed at 25 °C. All potentials are referenced to the FcH/FcH^+^ redox couple.^79^ The CV data of all compounds measured at a scan rate of 100 mV s^−1^ are summarized in Table 3 and Table SI6 (in the Supporting Information).

**Table 3:** CV data of **3**, **5 a**,**b**, **7 a**,**b**, **10**, and **12**. All values are given in mV vs. FcH/FcH^+^.^[a]^

Compound	*E*°_1_′^[b]^ (Δ*E*_p_)^[c]^	*E*°_2_′^[b]^ (Δ*E*_p_)^[c]^	*E*°_aryl_′′^[b]^ (Δ*E*_p_)^[c]^	Δ*E*°′^[d]^
**3**	30 (82)		1080 (103)	
**5 a**	−130 (65)		950 (68)	
**5 b**	20 (60)	95 (51)	1250 (97)	100^[e]^
**7 a**	50 (72)		1250 (95)	
**7 b** ^*f*^	20 (100)			90^[e]^
**10**	160 (84)			70^[e]^
**12**	30 (62)		1110 (94)	80^[e]^

[a] Conditions: potentials vs. FcH/FcH^+^, scan rate 100 mV s^−1^, at glassy carbon electrodes of **3**, **5 a**,**b**, **7 a**, **10**, and **12** (1.0 mmol L^−1^ [N*n*Bu_4_][B(C_6_F_5_)_4_] as supporting electrolyte) in anhydrous dichloromethane at 25 °C. [b] *E*°′=Formal potential. [c] Δ*E*_p_=Difference between the oxidation and the reduction potential. [d] Δ*E*°′=Potential difference between the two Fc‐related redox processes. [e] Determined by using deconvolution of the redox separation of the oxidation potentials in SWV (Figures SI15–SI16 in the Supporting Information) and the method by Richardson and Taube.^81^ [f] The analyte concentration was set to 0.1 mmol L^−1^.

**Figure 6:**
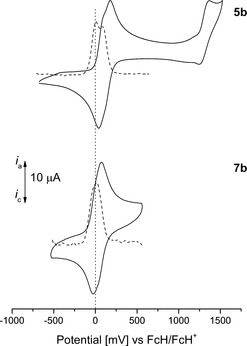
Cyclic (solid line) and square wave (dotted line) voltammograms (CV: potential area −1000 to 1750 mV; SW: potential area −1000 to 1750 mV) of **5 b** and **7 b**. Conditions: scan rate=100 (CV), 2.5 mV s^−1^ (SW) in dichloromethane solutions (**5 b**, 1.0 mmol L^−1^; **7 b**, 0.1 mmol L^−1^), supporting electrolyte=0.1 mol L^−1^ [N*n*Bu_4_][B(C_6_F_5_)_4_], working electrode=glassy carbon.

**Figure 7:**
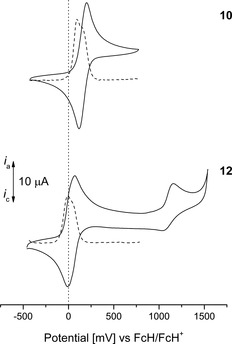
Cyclic (solid line) and square wave (dotted line) voltammograms (CV: potential area −1000 to 1750 mV; SW: potential area −1000 to 1750 mV) of **10** and **12**. Conditions: scan rate=100 (CV), 2.5 mV s^−1^ (SW) in dichloromethane solutions (1.0 mmol L^−1^), supporting electrolyte=0.1 mol L^−1^ [N*n*Bu_4_][B(C_6_F_5_)_4_], working electrode=glassy carbon.

The Fc groups of 9‐ferrocenylphenanthrene and **3** (Figure SI13 in the Supporting Information) as well as **5 b**, **7 b**, **10**, and **12** (Figure 6 and Figure 7; for the respective Br derivatives **5 a** and **7 a**, see Figure SI14 in the Supporting Information) show one (**3**, **5 a**, and **7 a**) or two (**5 b**, **7 b**, **10**, and **12**) reversible redox processes. The aromatic cores are characterized by a reversible (**5 a**) or irreversible redox event (**3**, **5 b**, **7 a**, and **12**) between 950 and 1250 mV, however, for **7 b**, **10**, and 9‐ferrocenylphenanthrene no oxidation of the aromatic core occurred under the applied measurement conditions.

The redox potentials of the monoferrocenyl‐substituted compounds are observed between −130 and 50 mV depending on the substituents (**5 a**, **7 a**), the substitution pattern (**3**, **5 a**, **7 a**) and the nature of the aromatic core (Table 3).

Within the series of monoferrocenyl‐substituted pyrenes, compound **5 a** can be oxidized at the lowest potential (−130 mV vs. FcH/FcH^+^). Comparison with **3** shows that the bromo substituent has a strong impact on the electron density at Fc, shifting the potential by 160 mV anodically. The Fc‐based redox process of **7 a** is further shifted to 50 mV (vs. FcH/FcH^+^) compared with **3**. Positions 2 and 7 are electron‐poor^80^ and hence the impact of the electron‐withdrawing bromine atom strongly shifts the redox potential cathodically compared with **5 a**. This observation is in contrast to the investigations of Fc‐substituted naphthalenes, where the influence of the substitution pattern is stronger than the impact of electron‐withdrawing entities like bromine.^44^ The nature of the aromatic core only has a minor effect on the electrochemical behavior, as evident from the similar oxidation potentials of **3** and 9‐ferrocenylphenanthrene, respectively. Dhokale et al. reported that 1‐ferrocenylpyrene and 1‐ferrocenylethynylpyrene show Fc‐based oxidations at 50 and 70 mV when using [N*n*Bu_4_][PF_6_] as the electrolyte versus a saturated calomel electrode (SCE).^9^ Furthermore, similar redox potentials were observed for monoferrocenyl‐substituted naphthalenes when using [N*n*Bu_4_][B(C_6_F_5_)_4_] as a weakly coordinating electrolyte.^44^

**Figure 8:**
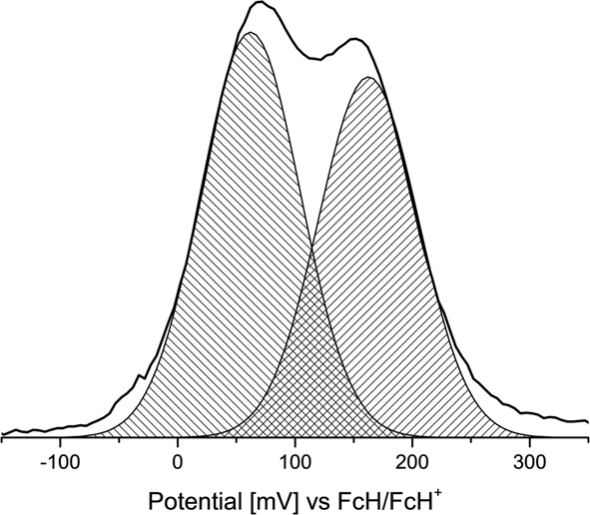
Deconvolution of the square wave voltammogram (SW: potential area −200 to 400 mV) of **5 b** to determine the redox separation according to the method by Richardson and Taube.^81^ Conditions: scan rate=2.5 mV s^−1^ (SW) in dichloromethane solutions (1.0 mmol L^−1^), supporting electrolyte=0.1 mol L^−1^ [N*n*Bu_4_][B(C_6_F_5_)_4_], working electrode=glassy carbon.

Diferrocenyl pyrenes **5 b** and **7 b** show a single redox event for both ferrocenyl groups at 20 mV (vs. FcH/FcH^+^; Figure 6). However, the relatively large Δ*E*_p_ values (Table 3) point to redox processes occurring in close potential proximity. Hence, the method of Richardson and Taube^81^ was used to determine Δ*E*°′ values of 90 (**7 b**) and 100 mV (**5 b**; Figure 8 and Figure SI15 in the Supporting Information). Kaafarani and co‐workers explored the electrochemical behavior of 2,7‐bis(carbazol‐9‐yl)pyrene and 2,7‐bis(3,6‐di‐*tert*‐butyl‐9 *H*‐carbazol‐9‐yl)pyrene by using [N*n*Bu_4_][PF_6_] as the electrolyte.^50^, ^82^ They observed a redox separation of 80 (2,7‐bis(3,6‐di‐*tert*‐butyl‐9 *H*‐carbazol‐9‐yl)pyrene) to 100 mV (2,7‐bis(carbazol‐9‐yl)pyrene), which is in good agreement with the results observed for **5 b** and **7 b**.^50^, ^82^ The group of Rao and Liu investigated the redox splitting of 2,7‐((η^5^‐C_5_Me_5_)(dppe)RuC≡C)_2_‐pyrene, which showed a value of 120 mV.^83^

Compounds **10** and **12** were synthesized to investigate the influence of electron‐withdrawing and ‐donating groups on the redox potential of the ferrocenyl oxidation. The methoxy‐functionalized compound **12** shows a redox process at 30 mV (vs. FcH/FcH^+^). In contrast to that, the electron‐withdrawing keto groups in positions 9 and 10 of **10** decrease the electron density of the Fc entities and therefore shift the respective potential by 130 mV anodically. The redox separation of **10** and **12** is lower than for the appropriate diferrocenyl‐substituted pyrenes **5 b** and **7 b**. By deconvolution of the SW voltammograms, redox splittings of 70 (**10**) and 80 mV (**12**) were found, pointing to a weak metal–metal interaction based on either electrostatic interactions or electron transfer (Table 3, Figure SI16 in the Supporting Information).^81^

For the purpose of classification, the electron transfer between the Fc/Fc^+^ termini in mono‐oxidized [**5 b**]^+^ and [**12**]^+^ spectroelectrochemical (in situ UV/Vis/NIR) measurements were performed. Compound **7 b** could not be investigated by this method, owing to its low solubility. Furthermore, the UV/Vis/NIR investigations of **10** revealed no intervalence charge transfer (IVCT) absorption but a MLCT (metal‐to‐ligand charge transfer) transition, which is in good agreement with the postulated resonance structures (Scheme SI2 in the Supporting Information).

**Figure 9:**
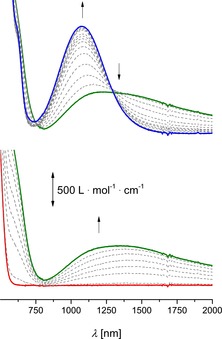
UV/Vis/NIR spectra of **5 b** at 25 °C in dichloromethane (2.00 mmol L^−1^) at rising potentials (bottom: −200 to 575 mV; top: 575 to 875 mV vs. Ag/Ag^+^); supporting electrolyte=[N*n*Bu_4_][B(C_6_F_5_)_4_]; arrows indicate the increasing and decreasing absorptions.

The UV/Vis/NIR spectroelectrochemical studies were carried out in dichloromethane (**5 b** and **12**) or propylene carbonate solutions (**12**) containing **5 b** or **12** (2.0 mmol L^−1^) and [N*n*Bu_4_][B(C_6_F_5_)_4_] (0.1 mol L^−1^) as supporting electrolyte in an OTTLE (optical transparent thin‐layer electrochemical) cell.^84^ A stepwise increase in the potentials (step widths 25, 50, and 100 mV) resulted in subsequent oxidation of **5 b** and **12**. During the measurements, oxidation of the neutral compounds to mixed‐valent [**5 b**]^+^ and [**12**]^+^ and finally to the fully oxidized species [**5 b**]^2+^ and [**12**]^2+^ was observed (Figure 9, Figures SI20, SI24, and SI25 in the Supporting Information). After complete oxidation, each compound was reduced at −200 mV to prove the reversibility of the process. It was found that the resulting UV/Vis/NIR spectra of **5 b** and **12** were identical to those of the neutral compounds.

For neutral **5 b** and **12**, as expected, no absorptions between 800 and 3000 nm were detected, whereas mono‐oxidation of **5 b** and **12** (*E*≈500 mV vs. Ag/Ag^+^) gave mixed‐valent species [**5 b**]^+^ and [**12**]^+^ and hence a broad absorption within this area (800–3000 nm) was observed. A further increase in the potential resulted in a decrease of this band, which is characteristic for intervalence charge transfer excitations (Figure 9, Figures SI24 and SI25 in the Supporting Information).^85^, ^86^, ^87^, ^88^, ^89^ Please note that the low Δ*E*°′ values (**5 b**: 100 mV, **12**: 80 mV) and therefore the small comproportionation constants (**5 b**: *K*_c_=70.0, **12**: *K*_c_=29.9) lead, in the best case, to a mixture consisting of an equilibrium of 73 % mixed‐valent monocationic [**12**]^+^ as well as 13.5 % neutral **12** and di‐cationic species [**12**]^2+^ each. As the composition of this equilibrium is not determinable by using an OTTLE cell, the extinction values are associated with considerable uncertainties.

The observed spectra were deconvoluted into three Gaussian‐shaped bands, which represent a LF (ligand field),^17^ a LMCT,^3^ and the IVCT absorptions of **5 b** and **12**. The NIR data of the IVCT absorptions are summarized in Table 4.

**Table 4:** NIR data of the IVCT absorptions of [**5 b**]^+^ and [**12**]^+^.^[a]^

Compound	*ν*_max_ [cm^−1^] (*ϵ*_max_ [L mol^−1^ cm^−1^])	Δ*ν*_1/2_ [cm^−1^]	Δ*ν*_1/2(theo)_^[b]^ [cm^−1^]
[**5 b**]^+^	7085 (510)	4150	4045
[**12**]^+^	10 095 (835)	3020	4829
[**12**]^+[c]^	10 990 (625)	2620	5037

[a] Conditions: in anhydrous dichloromethane at 25 °C; supporting electrolyte [N*n*Bu_4_][B(C_6_F_5_)_4_]. [b] Values calculated as Δ*ν*_1/2(theo)_=(2310 *ν*_max_)^1/2^ according to the Hush relationship for weakly coupled systems.^90^ [c] 2.00 mmol L^−1^ of **12** in propylene carbonate at 25 °C; supporting electrolyte [N*n*Bu_4_][B(C_6_F_5_)_4_].

Neutral **5 b** and **12** showed no absorption in the NIR region, whereas mixed‐valent [**5 b**]^+^ and [**12**]^+^ have weak and broad absorptions between 750 and 2000 nm. The IVCT bands of Fc‐substituted aromatics **5 b** and **12** exhibit different characteristics (Table 4). For **5 b**, three different spectra were investigated to identify the highest intensity of the metal–metal interaction band, owing to the overlap of IVCT and LMCT (Figure SI20 in the Supporting Information). The highest intensity of 510 L mol^−1^ cm^−1^ was found for 575 mV (vs. Ag/Ag^+^). From Figure SI20 (in the Supporting Information), it can be seen that in the series of increasing potential the IVCT absorption increases until its maximum at 575 mV, followed by a decrease of intensity upon further oxidation to [**5 b**]^2+^ and an increase of LMCT. The determined values of the IVCT absorption of **5 b** are in good agreement with 2,7‐bis(3,6‐di‐*tert*‐butyl‐9 *H*‐carbazol‐9‐yl)pyrene^82^ and therefore can be classified as a weakly coupled class II system according to Robin and Day.^91^

The smaller π‐conjugated bridge present in **12** leads to a more intense and narrow IVCT absorption (*ϵ*_max_≈835 L mol^−1^ cm^−1^, Δ*ν*_1/2_≈3020 cm^−1^) than in Fc_2_‐substituted pyrene **5 b** and is further shifted hypsochromically compared with the metal–metal interaction band of **5 b**. This statement is based on the higher electron density of the dimethoxy‐substituted phenanthrene moiety, which was observed in the CV measurements (Table 3). The phenanthrenyl bridge of **12** is oxidized more easily (1110 mV vs. FcH/FcH^+^) than the pyrenyl connectivity of **5 b** (1250 mV vs. FcH/FcH^+^; Table 3). Furthermore, the higher IVCT absorption of **12** shows a higher oscillator strength of 11.6×10^−3^ cm^−1^, determined from Equation (2), confirming a higher electron density of the phenanthrene core in **12** than of the pyrene unit in **5 b** (*f*=9.7×10^−3^ cm^−1^).

(2) $$f=4.6\times 10{}^{-9}\in {}_{\max}\Delta \nu {}_{1/2}$$

Within these species, the IVCT bands typically show strong solvatochromism.^85^, ^92^ To investigate this behavior, propylene carbonate (*μ*=4.9 D) was further chosen as an additional solvent for the spectroelectrochemical measurements of **12**. Analogous investigations on **5 b** failed in the UV/Vis/NIR setup, owing to its insufficient solubility in propylene carbonate. In the case of [**12**]^+^, the IVCT transition could be detected at approximately 11 000 cm^−1^ by using the solvent with higher polarity, revealing a solvatochromic shift (Δ*ν*_max_) of 1000 cm^−1^ (Table 4, Figures SI24 and SI25 in the Supporting Information). The magnitude of Δ*ν*_max_ in comparison for very strongly coupled systems (Δ*ν*_max_≈100 cm^−1^)^93^ confirms a class II classification according to Robin and Day.^91^

To gain a deeper insight into the communication between the aromatic core and the ferrocenyl ligands, the ligand‐to‐metal charge transfer properties of **3**, **5 a**,**b**, **7 a**, and **12** were investigated, except for **10**, which showed a MLCT (Figure SI23 in the Supporting Information). The UV/Vis/NIR spectra are depicted in Figures SI17–SI19, SI21, SI22, SI24, and SI25 in the Supporting Information). The respective LMCT data of [**3**]^+^, [**5 a**]^+^, [**5 b**]^2+^, [**7 a**]^+^, and [**12**]^2+^ are summarized in Table 5.

**Table 5:** NIR data of the LMCT absorptions of compounds [**3**]^+^, [**5 a**]^+^, [**5 b**]^2+^, [**7 a**]^+^, and [**12**]^2+^.

Compd.	*ν*_max_ [cm^−1^]^[b]^ (*ϵ*_max_ [L mol^−1^ cm^−1^])^[c]^	Δ*ν*_1/2_ [cm^−1^]^[d]^
[**3**]^+^	8510 (890)	2590
[**5 a**]^+^	8795 (1030)	2750
[**5 b**]^2+^	9140 (1230)	2730
[**7 a**]^+^	11 880 (970)	2550
[**12**]^2+^	10 155 (1820)	2675
[**12**]^2+[a]^	10 970 (875)	2790

[a] Conditions: 2.00 mmol L^−1^ of **12** in propylene carbonate at 25 °C; supporting electrolyte [N*n*Bu_4_][B(C_6_F_5_)_4_]. [b] *ν*_max_=Position of LMCT absorption. [c] *ϵ*_max_=Intensity of the LMCT absorption. [d] Δ*ν*_1/2_=Full width at half height of LMCT absorption.

The energy of the LMCT absorptions is related to the redox potentials of the Fc entities, displaying that with an increase of *E*°′, the transition energy *ν*_max_ is increased. The only exception is 1‐ferrocenylpyrene **3**, which shows an anodic shift of its redox event and its transition at 8150 cm^−1^. This might be based on the fact that the plane intersection of **3** in the solid state exhibits a rms value of 0.0348, which indicates a bent aromatic core. Further, the carbon atom C11 shows the highest displacement with 0.0685(17) Å, confirming a weak interaction between the Fc and the arene moieties (Table SI5 in the Supporting Information). Equivalent correlations have been reported for charge transfer complexes, for example, ferrocenyl naphthalenes,^44^ ferrocenyl thiophenes,^94^ diferrocenyl thiophenes,^18^ (oligo)pyrroles,^70^ and diverse (oligo)ferrocenyl thiophenes.^17^, ^95^, ^96^ By comparison of **5 a** with **7 a**, it is clear that a better communication between the Fc and the pyrene building blocks is given by the substitution in positions 1 and 6 than in 2 and 7, owing to higher intensity of the transition for **5 a**.

Based on the spectroelectrochemical properties (IVCT, LMCT; see above) and that the pyrene connectivity is favored to non‐covalently bind to CNTs,^25^, ^26^ we chose compound **5 b** for disentangeling and modification studies of the SWCNTs surface. Hence, DFT calculations were additionally carried out to support the respective pyrene–SWCNT interactions.

### DFT calculations of the interactions between 5 b and SWCNTs

**Figure 10:**
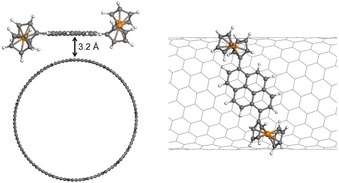
Top view (left) and side view (right) of the optimized structure for the adsorption of **5 b** on (14,7)‐SWCNT computed at the PBE‐D level. The white, orange, and gray spheres represent the H, Fe, and C atoms, respectively.

Density functional theory (DFT) calculations were performed to provide mechanistic insights into the interactions between **5 b** and SWCNTs. Here, we have considered both semiconducting (14,7)‐SWCNT and metallic (6,6)‐SWCNT as representative models of SWCNTs. Figure 10 shows the optimized structure for the adsorption of **5 b** on (14,7)‐SWCNT. It was found that the pyrene group of **5 b** tends to orient parallel to the CNT surface owing to optimized π–π interactions. The computed pyrene–CNT equilibrium distance and adsorption energy are 3.2 Å and −1.26 eV by using the PBE (Perdew–Burke–Ernzerhof) functional with empirical dispersion corrections (PBE‐D). In contrast, the plain DFT‐PBE calculations predict a much larger pyrene–CNT equilibrium distance (4.1 Å) and a negligible adsorption energy (−0.1 eV). These data indicate that the van der Waals forces are the dominant interactions between **5 b** and SWCNTs. This conclusion was also observed for the adsorption of **5 b** on a (6,6)‐SWCNT surface, suggesting that the diameter and chirality of CNTs have only a small influence on the adsorption mechanism. Additionally, Mulliken population analysis was employed to study the charge transfer in the ground state. The calculation results reveal that only a few electrons (about 0.1 *e*) are transferred from **5 b** to the CNT, which further confirms their π–π interactions.

### Disentangling experiments of SWCNTs with 5 b

To investigate the ability of **5 b** to interact with SWCNTs in a non‐covalent fashion in solution, debundeling experiments were performed with two commercially available materials: (i) chirality‐enriched (6,5)‐SWCNTs as powder (Sigma–Aldrich, batch number #MKB76336V) and (ii) NanoIntegris IsoSol S‐100® SWCNT in dispersion. (6,5)‐SWCNTs are, owing to their low purity, not suitable for electronic components but are an appropriate model system for the disentangling. However, the NanoIntegris IsoSol S‐100® SWCNT dispersion, consisting of up to 99.9 % semiconducting material, has proven to be an appropriate material for the scalable fabrication of CNT transistors and sensors^97^, ^98^, ^99^ but is stabilized in a chemically more complex manner.

**Figure 11:**
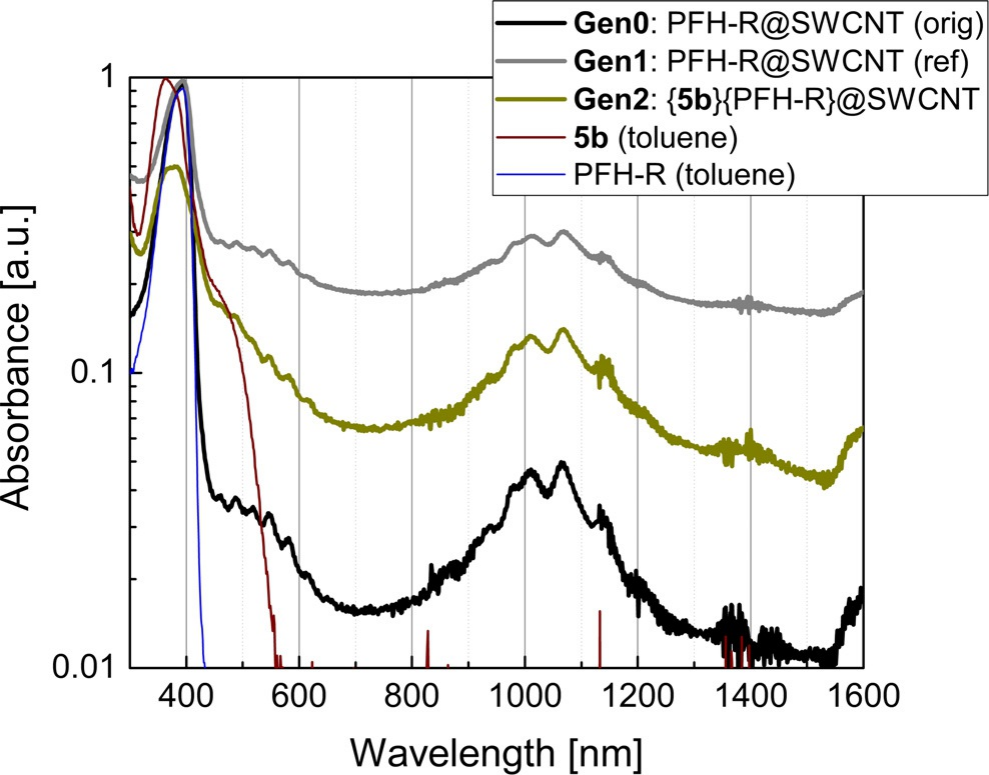
UV/Vis/NIR spectra of the (diluted) original SWCNT dispersion stabilized by PFH‐R (**Gen0**), a reference dispersion obtained from a vacuum‐filtrated solid with a slightly depleted PFH‐R content (**Gen1**), and a re‐dispersed SWCNT solid after PFH‐R depletion and **5 b** flushing (**Gen2**). Spectra of **5 b** in toluene and PFH‐R in toluene are given for comparison. Spectra **Gen0**, **Gen1**, **5 b** (toluene), and PFH‐R (toluene) are normalized to unity absorbance, spectrum **Gen2** is normalized to 0.5 for visibility reasons. (PFH‐R=9‐(9,9‐dihexyl‐9 *H*‐fluoren‐2‐yl)aryl‐based polymer).^100^

As indicated above, compound **5 b** was used for disentangling experiments with the NanoIntegris IsoSol S‐100® SWCNT dispersion and the results thereof are summarized in Figure 11. The investigations with chirality‐enriched (6,5)‐SWCNTs and **5 b** are presented in Figure SI26 (in the Supporting Information).

**Scheme 4:**
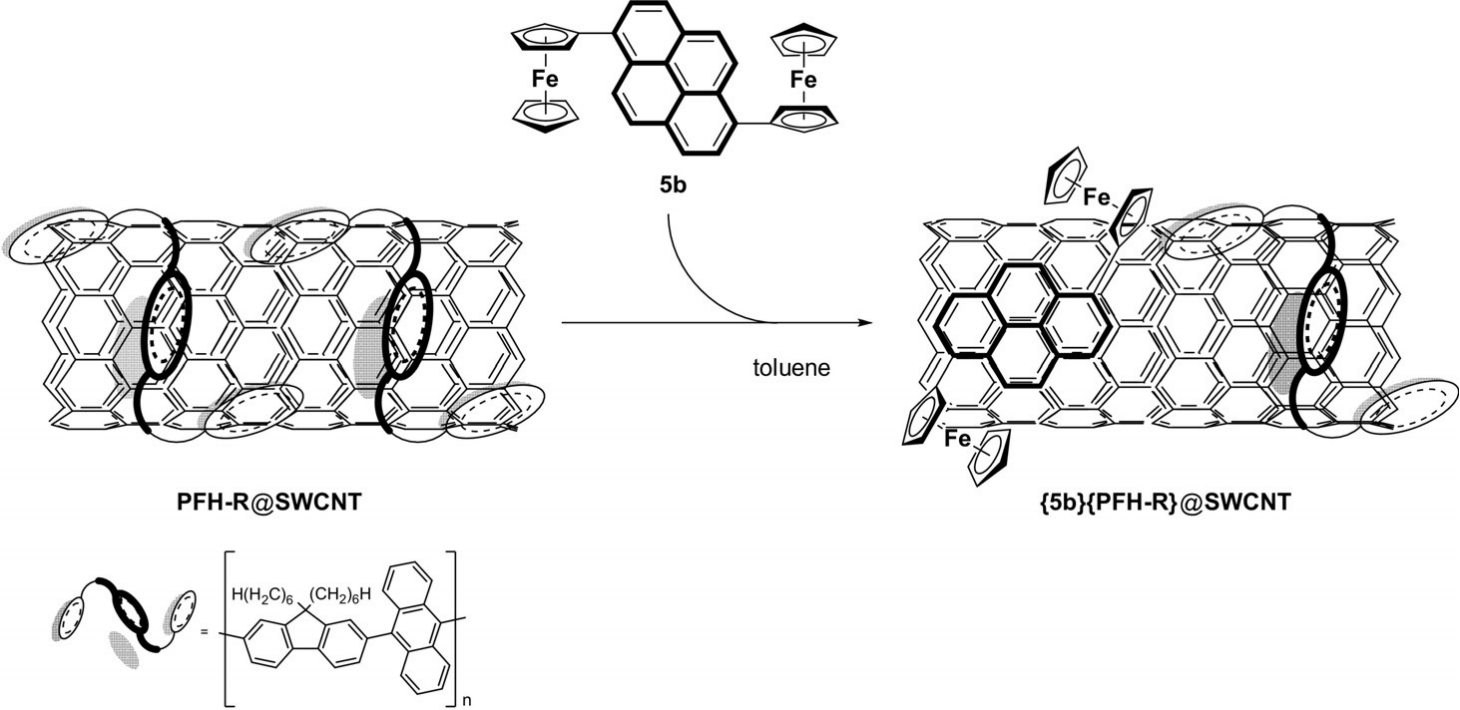
Exchange process for the replacement of the original dispersant by **5 b** for the debundeling of a commercial SWCNT dispersion (containing PFH‐R). Left) Commercial SWCNT dispersion, initial state. Right) Shell of PFH‐R partially replaced by **5 b** (PFH‐R=9‐(9,9‐dihexyl‐9 *H*‐fluoren‐2‐yl)aryl‐based polymer).^100^

For the debundeling of SWCNTs, NanoIntegris IsoSol S‐100® SWCNT dispersions were used in which the original containing polymeric dispersant was replaced by **5 b** by using a vacuum filtration procedure (Scheme 4, reference 29, Experimental Section; Figure 11).

In spectrum **Gen0** (Figure 11), the S22 transitions in the spectral range 900–1200 nm and the S33 transitions in the spectral range 450–650 nm of the chirality‐mixed SWCNTs are visible. At 392 nm, the optical transition of PFH‐R (9‐(9,9‐dihexyl‐9 *H*‐fluoren‐2‐yl)aryl‐based polymer)^100^ occurs. For the **Gen1** solid, both the S22 and S33 transitions of the SWCNT mixture as well as the PFH‐R transition remain unchanged from a spectral point of view, the relative composition of the absorption spectrum allows us to conclude a first successful depletion step for the reference solid through the vacuum filtration procedure. For the **Gen2** solid, the spectrum clearly mimics the SWCNT signature from the **Gen0** and **Gen1** spectra as well as the particular PFH‐R transition (spectrum PFH‐R (toluene) mathematically deconvoluted from the spill liquid and the **Gen0** spectrum). Additionally, the spectrum for the **Gen2** solid shows a transition at 352 nm, which can be identified as an absorption feature of **5 b** (measured spectrum **5 b** (toluene)), which unambiguously indicates that **5 b** is present in the redispersed SWCNT solid after the vacuum filtration procedure.

### Electrochemistry on dispersion Gen2

The functionalization of SWCNTs with **5 b** was further confirmed by electrochemical measurements. The redox properties of the dispersion **Gen2** ({**5 b**}{PFH‐R}@SWCNT) were investigated by cyclic voltammetry by using **Gen2**‐modified graphene paper^101^ as the working electrode (Experimental Section). The supporting electrolyte consists of an aqueous solution containing 1.0 mol L^−1^ KCl. The voltammetry measurements were performed at 25 °C. All potentials are referenced to the SCE using K_3_[Fe(CN)_6_] as the internal standard.^102^ The CV data are summarized in Table 6.

**Table 6:** CV data of dispersion **Gen2** by using graphene paper as the working electrode.^[a]^

Scan rate [mV s^−1^]	*E*°′^[b]^ (Δ*E*_p_)^[c]^
50	365 (41)
75	370 (41)
100	380 (38)
150	380 (47)
200	380 (51)
250	375 (55)
100	310 (173)^[d]^

[a] Conditions: potentials vs. SCE, **Gen2**@graphene as the working electrode (1.0 mol l ^−1^ KCl as supporting electrolyte) in water at 25 °C. [b] *E*°′=Formal potential. [c] Δ*E*_p_=Difference between the oxidation and the reduction potential. [d] Potential vs. SCE of **Gen2** after irreversible oxidation of pyrene.

**Figure 12:**
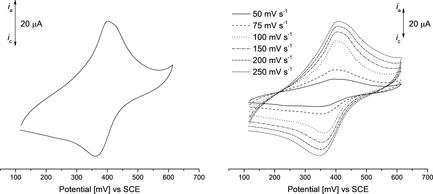
Cyclic voltammograms (potential area 100 to 600 mV) of **Gen2** with a scan rate of 100 mV s^−1^ (left) and at various scan rates of 50, 75, 100, 150, 200, and 250 mV s^−1^ (right). Aqueous solution of KCl (1.0 mol L^−1^) as supporting electrolyte, working electrode=modified graphene paper.

The CVs of **Gen2** show a ferrocenyl‐based redox process at 380 mV (vs. SCE, Figure 12), indicating the presence of **5 b** at the sidewalls of the SWCNTs. Moreover, the redox process is quite stable, as no significant decrease of the peak current is observable within multiple cycles (Figure SI28 in the Supporting Information).

For surface‐confined redox couples the linear relationship between the peak currents and the scan rates as well as the Δ*E*_p_ value of the respective redox processes are characteristic.^42^, ^103^ Therefore, the ferrocenyl‐based redox process was investigated with various scan rates (50–250 mV s^−1^; Table 6, Figure 12). The relationship between the peak currents and the scan rates was determined to be exponential with an exponent of 0.62 (*i*≈*v*_r_^0.62^; *v*_r_=sweep rate), which is far from a linear relationship, but also does not reflect the *i*≈√*v*_r_ of a fully dissolved redox system. The redox processes of dispersion **Gen2** show Δ*E*_p_ values between 40 and 55 mV (Table 6), which differ from the theoretical value of 0 V for immobilized redox systems,^103^ but is smaller than the Δ*E* of 57 mV for one‐electron processes.^103^, ^104^

To exclude that the redox process of **5 b** is based on interactions between **5 b** and graphene, **5 b**@graphene was additionally electrochemically characterized, which showed no ferrocenyl‐based redox processes (Figure SI27 in the Supporting Information). This emphasizes that **5 b** is exohedrally attached to the SWCNTs. In **Gen2**, where **5 b** is π‐bonded to SWCNTs, but not immobilized at the electrode surface, the above criteria for surface‐confined redox couples are unfulfilled, whereas simultaneously the electrochemical system **Gen2** does not behave accordingly to homogeneously dissolved redox‐active molecules.

**Figure 13:**
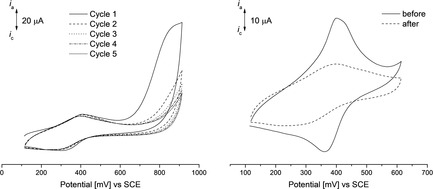
Cyclic voltammograms of **Gen2** in the potential area 100 to 900 mV (left), showing the irreversible pyrene oxidation and CVs of the ferrocenyl‐based oxidation of **Gen2** (right, potential area: 100 to 600 mV) before (solid) and after (dashed) pyrene oxidation. Scan rate=100 mV s^−1^ in aqueous solution of KCl (1 mol L^−1^) as supporting electrolyte, working electrode=modified graphene paper.

To investigate the influence of the pyrene core towards the ferrocenyl‐based redox process, the potential area was extended to 900 mV (Figure 13). An irreversible oxidation at 800 mV (vs. SCE) was observed, which decreases with the number of cycles and can be assigned to the oxidation of the pyrene.^42^ The irreversible oxidation of pyrene causes polymerization and hence oligopyrene is formed^42^, ^105^, ^106^ and hence the Fc‐based oxidation is cathodically shifted, indicating an increase of electron density at the Fc building blocks. The electrochemical Fc/Fc^+^ event is quite stable after polymerization, as the peak current decreases only slightly after five cycles (Figure SI28 in the Supporting Information).

## Conclusion

Compounds 1‐Fc‐ (**3**), 1‐Br‐6‐Fc‐ (**5 a**), 2‐Br‐7‐Fc‐ (**7 a**), 1,6‐Fc_2_‐pyrene (**5 b**), and 3,6‐Fc_2_‐9,10‐phenanthrenedione (**10**) were synthesized by reacting ferrocene boronic acid (**1**) with the respective bromo‐functionalized aromatics by applying the Pd‐catalyzed Suzuki C−C cross‐coupling protocol. The Negishi C−C cross‐coupling methodology allowed the formation of 2,7‐Fc_2_‐pyrene (**7 b**) and 3,6‐Fc_2_‐9,10‐dimethoxyphenanthrene (**12**).

The molecular structures of **3**, **5 a**,**b**, **7 a**,**b**, **10**, **12**, and, for comparison, 9‐Fc‐phenanthrene in the solid state are reported. The co‐planarity of the Fc entities towards the aromatic core was investigated for all compounds with regard to a higher degree of electron transfer between the π‐system of the Fc ligands through the arenes. The geometric characteristics of the positions 2 and 3 in **7 a**,**b**, **10**, and **12** allow a higher degree of planar intersections, owing to lower steric interactions of the Fc units with the H atoms in the respective positions (0.1(3) to 22.1(9)°). Parallel displaced π‐interactions were found for **10** and **12**, and **10** and **12** exhibit a columnar stacking between the aromatic building blocks, leading to the formation of 1D (**12**) or 2D coordination polymers (**10**) along the *a*‐ (**10**) or *b*‐axis (**12**).

Electrochemical studies of the Fc‐substituted aromatics **3**, **5 a**,**b**, **7 a**,**b**, **10**, and **12**, and 9‐ferrocenylphenanthrene for comparison, revealed a strong impact on the redox processes when bearing electron‐withdrawing or ‐donating functionalities such as Br or OMe units. Within the series **3**, **5 a**, and **7 a**, it could be shown that Br in position 1 influences the redox behavior most by shifting the potential cathodically from 50 mV in **7 a** to −130 mV in **5 a** (vs. FcH/FcH^+^). The Fc group in position 1 in **3** (30 mV vs. FcH/FcH^+^) is more easily to oxidize than the Fc in position 2 in **7 a** (*E*°′=50 mV). However, the 1,6‐ and 2,7‐substitution patterns of pyrenes **5 b** and **7 b** have no significant influence on the redox potentials and the redox splitting as was reported for Fc_2_‐naphthalenes,^44^ as well as 2,7‐bis(carbazol‐9‐yl)‐, 2,7‐bis(3,6‐di‐*tert*‐butyl‐9 *H*‐carbazol‐9‐yl)‐, and 2,7‐((η^5^‐C_5_Me_5_)(dppe)RuC≡C)_2_‐pyrenes.^50^, ^82^, ^83^ In contrast, the Fc/Fc^+^ redox potential of **10** and **12** differ as expected for electron‐withdrawing (C(=O)) or ‐donating (MeO) groups in positions 9 and 10 of the phenanthrene core. The redox splitting in the latter species is smaller than in pyrenes **5 b** and **7 b**, which is based on the closer proximity of the Fc ligands and the more electron‐poor π‐conjugated bridge in **10** and **12**.

To confirm the metal–metal interaction in **5 b**, **10**, and **12**, in situ UV/Vis/NIR studies were performed; **7 b** was excluded from these studies owing to its low solubility (see above). In the case of mono‐oxidized [**10**]^+^, the UV/Vis/NIR studies showed no IVCT absorption, which is due to the resonance structure of **10** and hence the redox separation is ascribed to electrostatic interactions. On the contrary, mixed‐valent [**5 b**]^+^ and [**12**]^+^ showed IVCT absorptions, however, of low intensity, indicating weakly coupled systems according to the classification by Robin and Day, which is in good agreement with, for example, 2,7‐bis(3,6‐di‐*tert*‐butyl‐9 *H*‐carbazol‐9‐yl)pyrene.^82^, ^91^ The IVCT transition of [**5 b**]^+^ appeared together with LMCT. Compound [**12**]^+^ showed a more intense and narrow IVCT absorption, indicating a stronger metal–metal interaction than for **5 b**. This might originate from the higher electron density at the Fc groups, owing to the electron‐donating OMe functionalities in positions 9 and 10 at phenanthrene. Furthermore, the IVCT transition of [**12**]^+^ was investigated in two different solvents, exploring its solvatochromic characteristics. A Δ*ν*_max_ shift of 1000 cm^−1^ was observed, confirming a class II classification according to Robin and Day.^91^

In addition, within the spectroelectrochemical studies, LMCT transitions were observed that are shifted bathochromically depending on the characteristics of the substituents and the substitution pattern. Comparing the intensities of the LMCT transitions of **5 a** with **7 a**, it becomes apparent that positions 1 and 6 at pyrene are favorable over positions 2 and 7. Moreover, the higher electron density of phenanthrene **12** bearing electron‐donating functionalities resulted in a more intense LMCT than in Fc_2_‐pyrene **5 b**. These findings are in good agreement with, for example, ferrocenyl naphthalenes,^44^ ferrocenyl thiophenes,^94^ diferrocenyl thiophenes,^18^ and (oligo)ferrocenyl‐pyrroles^70^ and ‐thiophenes.^17^, ^95^, ^96^

Solvatochromic studies on **10** showed a complex solvation of the push–pull system, reflecting the solvent properties such as hydrogen‐bond donor capacity, polarizability, and solvation, resulting in a bathochromic shift of *λ*_max_.

The interaction between **5 b** and SWCNTs was investigated by DFT calculations, which showed that the pyrene group of **5 b** is oriented parallel to the CNT surface based on optimized π–π interactions. The adsorption behavior of **5 b** is insignificantly influenced by the chirality or the diameter of the SWCNTs.

Based on the DFT calculations, the ability of **5 b** to interact non‐covalently with different SWCNTs (chirality‐enriched (6,5)‐SWCNTs and semiconducting SWCNTs) was investigated by disentangling experiments. The debundeling with chirality‐enriched (6,5)‐SWCNTs was performed for different sonication protocols (see the Supporting Information). For one parameter set, the dispersions containing SWCNTs and **5 b** in chloroform showed the typical S11 and the S22 transitions of disentangled SWCNTs in the UV/Vis/NIR spectra, which agree well with the reference spectra of a standard SWCNT dispersion, indicating a direct non‐covalent interaction between **5 b** and the SWCNT sidewalls.

With regard to the possible use of functionalized SWCNTs, integrated in CNT‐FETs, the interaction of **5 b** with semiconducting SWCNTS was studied (**Gen2**). As a result thereof, it was found that **5 b** is able to disentangle SWCNTs as evident from the appearance of the S22 and S33 transitions in the UV/Vis/NIR spectra.

The attachment of **5 b** in an exohedral fashion at the SWCNT sidewalls was proven by CV by using graphene paper as the working electrode, which showed a Fc/Fc^+^ redox event at 380 mV. This was further strengthened by the electrochemical investigations of solely **5 b**, showing no ferrocenyl‐based redox processes. At a potential of 800 mV, the irreversible oxidation of the pyrene core occurred, resulting in polymerization and subsequent formation of polypyrene oligomers.^42^, ^105^, ^106^

The debundeling and functionalization of SWCNTs by redox‐active Fc‐pyrenes in a non‐covalent manner is conceivable when using the 1,6‐substitution pattern at pyrene.

Based on these findings, compound **5 b** could be used in nanoelectronic application scenarios.

## Experimental Section

### Materials and methods

All synthesis procedures were performed under an atmosphere of argon with the solvents degassed prior to use. All reagents were obtained from commercial suppliers and used without further purification. The SWCNTs solid material was purchased from a commercial supplier (chirality‐enriched (6,5)‐SWCNTs, batch number #MKB76336V) and used without further treatment. A commercial SWCNT dispersion, NanoIntegris IsoSol S‐100®, batch SP31‐176, was purchased from commercial suppliers and used without any further purification. Ferrocene boronic acid was synthesized from lithioferrocene^45^ and triethyl borate on the basis of a procedure by Nesmejanow et al.^107^ 3,6‐Dibromo‐9,10‐dimethoxyphenanthrene was prepared according to the procedure published by Phulewale et al.^46^

NMR spectra were recorded with a Bruker Avance III 500 spectrometer operating at 500.3 MHz for ^1^H and 125.7 MHz for ^13^C{^1^H} in the Fourier transform mode at ambient temperature. Chemical shifts are reported in *δ* (ppm) downfield from tetramethylsilane with the solvent as the reference signal (^1^H NMR, CDCl_3_ *δ*=7.26 ppm; ^13^C{^1^H} NMR *δ*=77.16 ppm). FTIR spectra were recorded by using a Thermo Nicolet IR 200 instrument. The melting points were determined with a Gallenkamp MFB 595 010 m melting point apparatus. Elemental analyses were performed by applying a Thermos FlashAE 1112 instrument. High‐resolution mass spectra were recorded with a Bruker Daltonite micrOTOF‐QII spectrometer (electrospray ionization, Supporting Information). UV/Vis spectra between 210 and 1010 nm were recorded with a Carl Zeiss MCS 400 spectrometer utilizing CLD 300 (210–600 nm) and CLX 11 lamps (300–1010 nm). UV/Vis/NIR spectra of the SWCNT dispersions between 350 nm and 1300 nm were recorded by using a Shimadzu UV‐3100 PC absorption spectrometer on liquids in quartz cuvettes (optical path length 10.0 mm). For sonication, an Elmasonic P 30 H ultrasonic bath and a Bandelin KE76 tip sonotrode were used. Centrifugation of the dispersions was performed at a Sigma 3–30 K lab centrifuge in Oak Ridge tubes (VWR International) with 10 mL nominal volume.

### Electrochemistry

Electrochemical measurements of **3**, **5 a**,**b**, **7 a**,**b**, **10**, and **12** (1.0 mmol L^−1^ [N*n*Bu_4_][B(C_6_F_5_)_4_] as the supporting electrolyte) in dichloromethane were performed in a dried, argon‐purged cell at 25 °C. For the measurements, a three‐electrode cell containing a Pt auxiliary electrode, a glassy‐carbon working electrode (3.0 mm diameter), and an Ag/Ag^+^ (0.01 mmol L^−1^ [AgNO_3_]) reference electrode fixed on a Luggin capillary was used. The working electrode was pretreated by polishing it with a MicroFloc first with 1 μm and then with a 0.25 μm diamond paste. The reference electrode was constructed from a silver wire inserted in a 0.01 mmol L^−1^ [AgNO_3_] and a 0.1 mol L^−1^ [N*n*Bu_4_][B(C_6_F_5_)_4_] acetonitrile solution in a Luggin capillary with a Vycor tip. This Luggin capillary was inserted into a second Luggin capillary containing a 0.1 mol L^−1^ [N*n*Bu_4_][B(C_6_F_5_)_4_] dichloromethane solution and a Vycor tip. Experiments under the same conditions showed that all reduction and oxidation potentials were reproducible within ±5 mV. Experimental potentials were referenced against an Ag/Ag^+^ reference electrode, but the presented results are referenced against ferrocene as an internal standard as required by IUPAC.^79^ To achieve this, each experiment was repeated in the presence of 1 mmol L^−1^ decamethylferrocene (Fc*). Data were processed with a Microsoft Excel worksheet to set the formal reduction potentials of the FcH/FcH^+^ couple to 0.0 V. Under our conditions, the Fc*/Fc*^+^ couple appeared at −619 mV vs. FcH/FcH^+^, Δ*E*_p_=60 mV, whereas the FcH/FcH^+^ couple itself was at 220 mV vs. Ag/Ag^+^, Δ*E*_p_=61 mV.^108^ For the measurements of **5 b**@SWCNT (**Gen2**) nanoconjugates, a three‐electrode arrangement containing a Pt auxiliary electrode and an Ag/AgCl reference electrode (consisting of a silver wire covered with a thin film of AgCl) fixed in a glass tube (providing a pipetting controller for the supply of the electrolyte) at 25 °C was used.^109^ As the working electrode, graphene paper was used. For the electrochemical studies, the glass tube was fixed near the sample without direct contact points. With the help of the pipetting controller, the defined sample area was wetted with the electrolyte solution. Experimental potentials were referenced against an SCE, whereby the experimental arrangement was referenced against K_3_[Fe(CN)_6_] (5 mm aqueous solution) in 1 m aqueous KCl solution (0.358 V).^102^

### Spectroelectrochemistry

Spectroelectrochemical UV/Vis/NIR measurements of 2.0 mmol L^−1^ solutions of **3**, **5**, **7 a**, **10**, **12**, or 9‐ferrocenylphenanthrene in anhydrous dichloromethane containing 0.1 mol L^−1^ of [N*n*Bu_4_][B(C_6_F_5_)_4_] as the supporting electrolyte were performed in an OTTLE (optically transparent thin‐layer electrochemical)^84^ cell at 25 °C. The values obtained by deconvolution could be reproduced within *ϵ*_max_ ±100 L mol^−1^ cm^−1^, *ν*_max_ ±50 cm^−1^, and Δ*ν*_1/2_ ±50 cm^−1^.

### Single‐crystal X‐ray diffraction analysis

Diffraction data were collected with an Oxford Gemini S diffractometer using graphite‐monochromated Mo_Kα_ radiation (**3**, **4**, **5 a**,**b**, **7 a**, **10**, 9‐ferrocenylphenanthrene; *λ*=0.71073 Å) or Cu_Kα_ radiation (**7 b**, **12**; *λ*=1.54184 Å) at 110 K by using oil‐coated shock‐cooled crystals. The structures were solved by direct methods and refined by full‐matrix least‐squares procedures on *F* ^2^.^110^, ^111^, ^112^ All non‐hydrogen atoms were refined anisotropically, and a riding model was employed in the refinement of the hydrogen atom positions. Graphics of the molecular structures were created by using SHELXTL and ORTEP.^113^

### DFT Calculations

Density functional theory (DFT) calculations were performed with periodic models by using the CASTEP code.^114^ The exchange and correlation interactions were modeled by using the Perdew–Burke–Ernzerhof (PBE) functional^115^ within the generalized gradient approximation. To study the role of the van der Waals forces on the adsorption, an empirical dispersion correction (PBE‐D)^116^ as implemented in CASTEP was addressed. The wave functions of the valence electrons were expanded by using a plane‐wave basis set within a specified cutoff energy of 400 eV. Electron‐ion interactions were described by ultrasoft pseudopotentials.^117^ The Brillouin zone was sampled by Γ‐centered 1×1×2 Monkhorst–Pack k‐point mesh. The adsorption energy (*E*_ads_) of **5 b** on CNT is calculated by *E*_ads_=*E*_**5 b**/CNT_−*E*_**5 b**_−*E*_CNT_, where *E*_**5 b**/CNT_, *E*_**5 b**_, and *E*_CNT_ denote the total energies of the optimized structures of **5 b** adsorbed on CNT, gaseous **5 b**, and isolated CNT, respectively.

### Synthesis of 3, 5 a,b, 7 a, 9, and 10

#### General synthesis protocol for the Suzuki C−C cross‐coupling reactions, synthesis of 3, 5 a,b, 7 a, and 10

A three‐necked 100 mL flask was charged with [Pd(dppf)Cl_2_] (1 mol %), ferrocene boronic acid (**1**; 196 mg, 0.854 mmol, 1.2 equiv), K_3_PO_4_ **⋅**H_2_O (589 mg, 2.6 mmol, 2.5 equiv), and the respective aryl halide (1.0 equiv for 9‐bromophenanthrene and **2**, 0.5 equiv for **4**, **6**, and **9**). Anhydrous toluene (15 mL) was added. The reaction mixture was stirred for 5 min at ambient temperature and then heated to reflux for 24 h. After cooling to ambient temperature, the reaction mixture was filtered through a pad of silica. Afterwards, all volatiles were removed by evaporation. Purification was realized by column chromatography (silica, column size 4×25 cm) by using different dichloromethane/hexane mixtures (see below).

#### Synthesis of 1‐ferrocenylpyrene (3)^9^

The title compound was synthesized according to the general synthesis procedure described above by using 1‐bromopyrene (**2**; 200 mg, 0.711 mmol). Compound **3** was separated by column chromatography by using hexane/dichloromethane mixtures (v/v) starting from 9:1 (ferrocene, 98 mg, 0.53 mmol; 74 % based on 1‐bromopyrene) to 4:1 (**3**). After evaporation of all volatiles, **3** was obtained as an orange solid. Yield: 7.5 mg (0.019 mmol, 3 % based on **2**). M.p.: 205 °C; ^1^H NMR (CDCl_3_): *δ*=4.22 (s, 5 H, C_5_H_5_), 4.49 (t, *J*_HH_=1.8 Hz, 2 H, C_5_H_4_), 4.84 (t, *J*_HH_=1.8 Hz, 2 H, C_5_H_4_), 7.99 (t, *J*_HH_=7.6 Hz, 1 H, C_16_H_9_), 8.04–8.08 (m, 3 H, C_16_H_9_), 8.14–8.18 (m, 3 H, C_16_H_9_), 8.41 (d, *J*_HH_=8.0 Hz, 1 H, C_16_H_9_), 8.75 ppm (d, *J*_HH_=9.3 Hz, 1 H, C_16_H_9_); ^13^C NMR (CDCl_3_): *δ*=68.7 (C_5_H_4_), 69.9 (C_5_H_5_), 71.1 (C_5_H_4_), 87.4 (^q^C, C_5_H_4_), 124.5 (C_16_H_9_), 124.7 (C_16_H_9_), 125.0 (C_16_H_9_), 125.6 (C_16_H_9_), 126.1 (C_16_H_9_), 127.0 (C_16_H_9_), 127.1 (C_16_H_9_), 127.6 (C_16_H_9_), 128.9 (C_16_H_9_), 130.0 (C_16_H_9_), 131.1 (C_16_H_9_), 131.8 (C_16_H_9_), 134.2 ppm (C_16_H_9_); IR (KBr): *ν*=2957 (m), 2925 (s), 2854 (m), 1602 (w), 1508 (w), 1489 (w), 1430 (w), 1406 (w), 1264 (w), 1191 (w), 1105 (m), 1082 (m), 1051 (m), 1002 (m), 963 (w), 898 (w), 855 (s), 845 (s), 838 (s), 831 (s), 821 (s), 812 (m), 798 (m), 762 (m), 723 (m), 682 (m), 668 cm^−1^ (w). **Crystal data for 3**: C_26_H_18_Fe, *M*_r_=386.25 g mol^−1^, monoclinic, *C*2/*c*, *λ*=0.71073 Å, *a=*20.4388(8) Å, *b=*7.6777(4) Å, *c=*22.5396(9) Å, *V=*3490.4(3) Å^3^, *Z=*8, *ρ*_calcd_=1.470 mg cm^−3^, *μ*=0.871 mm^−1^, *T=*115.95(10) K, *θ* range 2.932–24.997°, 13603 reflections collected, 3063 independent reflections (*R*_int_ *=*0.0372), *R*1=0.0318, *wR*2=0.0726 (*I*>2σ(*I*)).

#### Synthesis of 1‐bromo‐6‐ferrocenylpyrene (5 a) and 1,6‐diferrocenylpyrene (5 b)

The compounds were synthesized according to the general synthesis protocol described above by using 1,6‐dibromopyrene (**4**; 144.0 mg, 0.4 mmol). Compounds **5 a** and **5 b** were separated by column chromatography (see above) by using hexane/dichloromethane mixtures (v/v) starting from 9:1 (ferrocene, 2 mg, 0.008 mmol, 2 % based on 1,6‐dibromopyrene) to 4:1 (**5 a**) and 1:9 (**5 b**). After evaporation of all volatiles, **5 a** and **5 b** were obtained as orange (**5 a**) or red solids (**5 b**).

**Compound** **5 a**: Yield: 120 mg (0.26 mmol, 65 % based on **4**). M.p.: 190 °C; ^1^H NMR (CDCl_3_): *δ*=4.22 (s, 5 H, C_5_H_5_), 4.50 (t, *J*_HH_=1.8 Hz, 2 H, C_5_H_4_), 4.83 (t, *J*_HH_=1.8 Hz, 2 H, C_5_H_4_), 7.99 (t, *J*_HH_=9.0 Hz, 2 H, C_16_H_8_), 8.11–8.27 (m, 3 H, C_16_H_8_), 8.44 (dd, *J*_HH_=10.2, 8.6 Hz, 2 H, C_16_H_8_), 8.76 ppm (d, *J*_HH_=9.3 Hz, 1 H, C_16_H_8_); ^13^C NMR (CDCl_3_): *δ*=68.9 (C_5_H_4_), 70.0 (C_5_H_5_), 71.2 (C_5_H_4_), 87.1 (^q^C, C_5_H_4_), 119.8 (C_16_H_8_), 124.5 (C_16_H_8_), 125.0 (C_16_H_8_), 125.2 (C_16_H_8_), 125.6 (C_16_H_8_), 125.9 (C_16_H_8_), 126.3 (C_16_H_8_), 126.6 (C_16_H_8_), 128.9 (C_16_H_8_), 129.2 (C_16_H_8_), 129.6 (C_16_H_8_), 129.7 (C_16_H_8_), 130.2 (C_16_H_8_), 130.3 (C_16_H_8_), 130.6 (C_16_H_8_), 135.3 ppm (C_16_H_8_); IR (KBr): *ν*=2960 (w), 2955 (w), 2925 (m), 1602 (w), 1508 (w), 1480 (w), 1464 (w), 1427 (w), 1378 (w), 1287 (w), 1262 (m), 1238 (w), 1162 (w), 1133 (w), 1105 (s), 1061 (s), 1031 (s), 959 (w), 910 (m), 853 (s), 848 (s), 832 (s), 816 (s), 805 (s), 717 (m), 681 (s), 668 (m), 629 cm^−1^ (m); HR‐MS (ESI‐TOF)*m*/*z* calcd for C_26_H_17_BrFe: 463.9859; found: 463.9849 [*M*]^+^; elemental analysis calcd for C_26_H_17_BrFe (465.16 g mol^−1^): C 67.13, H 3.68, found: C 67.69, H 4.76. **Crystal data for 5 a**: C_26_H_17_BrFe, *M*_r_=465.15 g mol^−1^, monoclinic, *I*2/*a*, *λ*=0.71073 Å, *a=*18.4055(14) Å, *b=*7.4788(6) Å, *c=*26.788(2) Å, *β*=96.988(7)°, *V=*3659.9(5) Å^3^, *Z=*8, *ρ*_calcd_=1.688 mg cm^−3^, *μ*=3.016 mm^−1^, *T=*116.8(6) K, *θ* range 3.565–24.992°, 8764 reflections collected, 3199 independent reflections (*R*_int_ *=*0.0352), *R*1=0.0327, *wR*2=0.0701 (*I*>2σ(*I*)).

**Compound** **5 b**: Yield: 72.3 mg (0.13 mmol, 32 % based on **4**). M.p.: 176 °C (decomposition); ^1^H NMR (CDCl_3_): *δ*=4.22 (s, 10 H, C_5_H_5_), 4.49 (t, *J*_HH_=1.8 Hz, 4 H, C_5_H_4_), 4.84 (t, *J*_HH_=1.8 Hz, 4 H, C_5_H_4_), 8.02 (d, *J*_HH_=9.3 Hz, 2 H, C_16_H_8_), 8.10 (d, *J*_HH_=8.0 Hz, 2 H, C_16_H_8_), 8.39 (d, *J*_HH_=7.9 Hz, 2 H, C_16_H_8_), 8.73 ppm (d, *J*_HH_=9.2 Hz, 2 H, C_16_H_8_); ^13^C NMR (CDCl_3_): *δ*=68.7 (C_5_H_4_), 69.9 (C_5_H_5_), 71.1 (C_5_H_4_), 87.6 (^q^C, C_5_H_4_), 124.1 (C_16_H_8_), 125.1 (C_16_H_8_), 125.4 (C_16_H_8_), 127.0 (C_16_H_8_), 129.0 (C_16_H_8_), 129.3 (C_16_H_8_), 129.7 (C_16_H_8_), 134.0 ppm (C_16_H_8_); IR (KBr): *ν*=2964 (m), 2925 (w), 2855 (w), 1601 (w), 1496 (w), 1456 (w), 1428 (w), 1409 (w), 1373 (w), 1262 (s), 1211 (w), 1105 (s), 1074 (s), 1022 (s), 881 (m), 866 (m), 854 (m), 804 (s), 684 (m), 668 cm^−1^ (m); HR‐MS (ESI‐TOF) *m*/*z* calcd for C_36_H_26_Fe_2_: 570.0729; found: 570.0734 [*M*]^+^; elemental analysis calcd for C_36_H_26_Fe_2_ (570.28 g mol^−1^): C 75.82, H 4.60; found: C 75.56, H 4.52. **Crystal data for 5 b**: C_36_H_26_Fe_2_, *M*_r_=570.27 g mol^−1^, monoclinic, *P*2_1_/*n*, *λ*=0.71073 Å, *a=*14.3748(18) Å, *b=*8.6113(9) Å, *c=*20.585(2) Å, *β*=105.022(12)°, *V=*2461.1(5) Å^3^, *Z=*4, *ρ*_calcd_=1.539 mg cm^−3^, *μ*=1.204 mm^−1^, *T=*116.95(10) K, *θ* range 3.043–24.996°, 5798 reflections collected, 5798 independent reflections (*R*_int_=0.0655), *R*1=0.0771, *wR*2=0.1997 (*I*>2σ(*I*)).

#### Synthesis of 2‐bromo‐7‐ferrocenylpyrene (7 a)

Compound **7 a** was prepared according to the general synthetic methodology described above by using 2,7‐dibromopyrene (**6**; 144.0 mg, 0.4 mmol). Compound **7 a** was separated by column chromatography by using hexane/dichloromethane mixtures (v/v) starting from 9:1 (ferrocene, 50 mg, 0.27 mmol; 67 % based on 2,7‐dibromopyrene) to 4:1 (**7 a**). After evaporation of all volatiles, the received solid was recrystallized from acetone at −20 °C. Compound **7 a** was isolated as an orange solid. Yield: 37 mg (0.08 mmol, 20 % based on **6**). M.p.: 223 °C; ^1^H NMR (CDCl_3_): *δ*=4.08 (s, 5 H, C_5_H_5_), 4.46 (t, *J*_HH_=1.8 Hz, 2 H, C_5_H_4_), 4.96 (t, *J*_HH_=1.8 Hz, 2 H, C_5_H_4_), 7.96 (d, *J*_HH_=9.0 Hz, 2 H, C_16_H_8_), 8.07 (d, *J*_HH_=9.0 Hz, 2 H, C_16_H_8_), 8.26 ppm (d, *J*_HH_=16.5 Hz, 2 H, C_16_H_8_); ^13^C NMR (CDCl_3_): *δ*=67.2 (C_5_H_4_), 69.7 (C_5_H_4_), 70.0 (C_5_H_5_), 85.3 (^q^C, C_5_H_4_), 119.7 (C_16_H_8_), 123.1 (C_16_H_8_), 123.4 (C_16_H_8_), 123.5 (C_16_H_8_), 126.7 (C_16_H_8_), 127.3 (C_16_H_8_), 128.7 (C_16_H_8_), 131.1 (C_16_H_8_), 132.6 (C_16_H_8_), 138.0 ppm (C_16_H_8_); IR (KBr): *ν*=3036 (w), 2924 (w), 2852 (w), 1597 (s), 1554 (m), 1487 (m), 1429 (m), 1409 (m), 1299 (m), 1245 (s), 1217 (w), 1152 (m), 1145 (m), 1105 (s), 1037 (m), 1030 (m), 998 (s), 929 (m), 898 (m), 878 (s), 867 (s), 858 (s), 853 (s), 846 (s), 830 (s), 820 (s), 803 (s), 762 (s), 707 (s), 669 cm^−1^ (s); HR‐MS (ESI‐TOF) *m*/*z* calcd for C_26_H_17_BrFe: 463.9859; found: 463.9849 [*M*]^+^; elemental analysis calcd for C_26_H_17_BrFe (465.16 g mol^−1^): C 67.13, H 3.68; found: C 67.25, H 3.71. **Crystal data for 7 a**: C_26_H_17_BrFe, *M*_r_=465.15 g mol^−1^, monoclinic, *P*2_1_/*c*, *λ*=0.71073 Å, *a=*10.3922(8) Å, *b=*13.9915(11) Å, *c=*12.8138(11) Å, *β*=99.217(3)°, *V=*1839.1(3) Å^3^, *Z=*4, *ρ*_calcd_=1.680 mg cm^−3^, *μ*=3.001 mm^−1^, *T=*100 K, *θ* range 3.525–24.997°, 26 828 reflections collected, 3234 independent reflections (*R*_int_=0.0982), *R*1=0.0268, *wR*2=0.0707 (*I*>2σ(*I*)).

#### Synthesis of 3,6‐diferrocenylphenanthrene‐9,10‐dione (10)

Compound **10** was synthesized in accordance with the general synthesis procedure described above by using 3,6‐dibromophenathren‐9,10‐dione (**9**; 311 mg, 0.85 mmol). Compound **10** was separated by column chromatography (alox) by using first hexane (ferrocene, 78 mg, 0.42 mmol, 49 % based on 3,6‐dibromophenathren‐9,10‐dione) and then dichloromethane/ethylacetate mixtures (v/v) of ratios 4:1 and 3:1 (**10**). After evaporation of all volatiles, compound **10** was obtained as a green solid. Yield: 136 mg (0.24 mmol, 28 % based on **9**). M.p.: 123 °C; ^1^H NMR (CDCl_3_): *δ*=4.13 (s, 10 H, C_5_H_5_), 4.55 (t, *J*_HH_=1.8 Hz, 4 H, C_5_H_4_), 4.86 (t, *J*_HH_=1.8 Hz, 4 H, C_5_H_4_), 7.56 (dd, *J*_HH_=8.1, 1.5 Hz, 2 H, C_14_H_6_), 8.05 (d, *J*_HH_=1.5 Hz, 2 H, C_14_H_6_), 8.15 ppm (d, *J*_HH_=8.1 Hz, 2 H, C_14_H_6_); ^13^C NMR (CDCl_3_): *δ*=67.6 (C_5_H_4_), 70.3 (C_5_H_5_), 70.9 (C_5_H_4_), 83.0 (^q^C, C_5_H_4_), 120.6 (C_14_H_6_), 127.1 (C_14_H_6_), 129.0 (C_14_H_6_), 130.9 (C_14_H_6_), 136.0 (C_14_H_6_), 149.5 (C_14_H_6_), 180.1 ppm (CO); IR (KBr): *ν*=2959 (m), 2925 (s), 2854 (m), 1774 (w), 1658 (m), 1590 (s), 1465 (m), 1416 (m), 1262 (s), 1105 (s), 1029 (s), 924 (m), 807 (s), 731 cm^−1^ (m); elemental analysis calcd for C_34_H_24_Fe_2_O_2_ (576.25 g mol^−1^): C 70.87, H 4.20; found: C 70.76, H 4.11. **Crystal data for 10**: C_35_H_26_Cl_2_Fe_2_O_2_, *M*_r_=661.16 g mol^−1^, monoclinic, *P*2_1_/*c*, *λ*=0.71073 Å, *a=*10.1023(5) Å, *b=*38.6119(18) Å, *c=*13.9755(8) Å, *β*=93.989(5)°, *V=*5438.2(5) Å^3^, *Z=*8, *ρ*_calcd_=1.615 mg cm^−3^, *μ*=1.297 mm^−1^, *T=*129.9(4) K, *θ* range 3.023–25.999°, 34 953 reflections collected, 10 644 independent reflections (*R*_int_=0.0537), *R*1=0.0819, *wR*2=0.1943 (*I*>2σ(*I*)).

### General synthesis procedure for the Negishi C−C cross‐coupling reactions, synthesis of 7 b and 12

A 1.9 m solution of *tert*‐butyllithium (4.6 mL, 7.5 mmol) in pentane was added dropwise at −30 °C to ferrocene (920 mg, 5 mmol) and KO*t*Bu (56 mg, 0.5 mmol) dissolved in tetrahydrofuran (20 mL). After 1 h of stirring at this temperature, anhydrous [ZnCl_2_ 2 thf] (2.2 g, 8 mmol) was added in a single portion. The solution was kept for 1 h at −30 °C and an additional hour at 25 °C. Afterwards, [PdCl_2_(dppf)] (35 mg, 0.03 mmol) and the respective dibromoarenes (**6**, **11**, 0.83 mmol) were added in a single portion and the reaction solution was stirred for 24 h at 60 °C. After evaporation of all volatiles, the precipitate was dissolved in dichloromethane (200 mL) and washed thrice with 100 mL portions of water. The organic phase was dried over MgSO_4_ and the solvent was removed under oil‐pump vacuum. The remaining solid was purified by column chromatography (silica, column size 4×25 cm) by using different dichloromethane/hexane mixtures. All volatiles were removed under reduced pressure. The title compounds were obtained as solids.

#### Synthesis of 2,7‐diferrocenylpyrene (7 b)

Compound **7 b** was synthesized according to the general synthetic methodology for the Negishi C−C cross‐coupling protocol by using 2,7‐dibromopyrene (**6**; 450 mg, 1.25 mmol). Compound **7 b** was separated by column chromatography by using hexane/dichloromethane eluent mixtures (v/v) starting from 9:1 (ferrocene, 212 mg, 1.14 mmol; 91 % based on 2,7‐dibromopyrene) to 1:9 (**7 b**). The obtained solid was recrystallized from acetone at −20 °C. Compound **7 b** was isolated as a red solid. Yield: 15 mg (0.027 mmol, 2 % based on **6**). M.p.: 265 °C (decomposition); ^1^H NMR (CDCl_3_): *δ*=4.08 (s, 10 H, C_5_H_5_), 4.45 (br s, 4 H, C_4_H_5_), 4.96 (br s, 4 H, C_4_H_5_), 8.04 (s, 4 H, C_16_H_8_), 8.24 ppm (s, 4 h, C_16_H_8_); IR (KBr): *ν*=3116 (w), 3092 (w), 1606 (m), 1424 (m), 1407 (m), 1382 (m), 1103 (s), 1030 (s), 1000 (s), 932 (m), 881 (m), 829 (m), 806 (m), 714 (m), 668 cm^−1^ (w); elemental analysis calcd for C_36_H_26_Fe_2_ (570.28 g mol^−1^): C 75.82, H 4.60; found: C 76.63, H 4.43. **Crystal data for 7 b**: C_36_H_26_Fe_2_, *M*_r_=570.27 g mol^−1^, monoclinic, *P*2_1_/*c*, *λ*=1.54184 Å, *a=*11.0921(3) Å, *b=*7.8868(2) Å, *c=*14.2973(4) Å, *β*=100.294(2)°, *V=*1230.61(6) Å^3^, *Z=*2, *ρ*_calcd_=1.539 mg cm^−3^, *μ*=9.630 mm^−1^, *T=*100 K, *θ* range 4.051–65.912°, 11 449 reflections collected, 2128 independent reflections (*R*_int_=0.0443), *R*1=0.0375, *wR*2=0.0961 (*I*>2σ(*I*)).

#### Synthesis of 3,6‐diferrocenyl‐9,10‐dimethoxyphenanthrene (12)

The title compound was prepared according to the general synthesis protocol for the Negishi C−C cross‐coupling by using 3,6‐dibromo‐9,10‐dimethoxyphenanthrene (**11**; 396 mg, 0.5 mmol). Compound **12** was separated by column chromatography by using hexane/dichloromethane eluent mixtures (v/v) starting from 9:1 (ferrocene, 73 mg, 0.39 mmol; 78 % based on 3,6‐dibromo‐9,10‐dimethoxyphenanthrene) to 1:9 (**12**). After evaporation of all volatiles, **12** was obtained as an orange solid. Yield: 100 mg (0.16 mmol, 13 % based on **11**). M.p.: 169 °C; ^1^H NMR (CDCl_3_): *δ*=4.10 (s, 10 H, C_5_H_5_), 4.12 (s, 6 H, OCH_3_), 4.43 (t, *J*_HH_=1.8 Hz, 4 H, C_5_H_4_), 4.86 (t, *J*_HH_=1.8 Hz, 4 H, C_5_H_4_), 7.80 (dd, *J*_HH_=8.5, 1.5 Hz, 2 H, C_14_H_6_), 8.16 (d, *J*_HH_=8.5 Hz, 2 H, C_14_H_6_), 8.68 ppm (d, *J*_HH_=1.5 Hz, 2 H, C_14_H_6_); ^13^C NMR (CDCl_3_): *δ*=61.2 (OCH_3_), 67.0 (C_5_H_4_), 69.4 (C_5_H_5_), 69.9 (C_5_H_4_), 86.0 (^q^C, C_5_H_4_), 119.4 (C_14_H_6_), 122.3 (C_14_H_6_), 126.1 (C_14_H_6_), 127.8 (C_14_H_6_), 128.5 (C_14_H_6_), 136.9 ppm (C_14_H_6_); IR (KBr): *ν*=2925 (s), 2854 (s), 1744 (m), 1700 (m), 1684 (m), 1653 (m), 1607 (s), 1559 (m), 1540 (m), 1507 (m), 1458 (s), 1446 (s), 1419 (m), 1337 (m), 1311 (s), 1189 (m), 1105 (s), 1092 (s), 1061 (s), 1027 (m), 983 (s), 885 (m), 874 (s), 707 (w), 668 (w), 661 cm^−1^ (w); elemental analysis calcd for C_36_H_30_Fe_2_O_2_ (606.32 g mol^−1^): C 71.31, H 4.99; found: C 70.50, H 4.89. **Crystal data for 12**: C_36_H_30_Fe_2_O_2_, *M*_r_=606.30 g mol^−1^, orthorhombic, *Pnn*2, *λ*=1.54178 Å, *a=*19.680(2) Å, *b=*6.3428(8) Å, *c=*10.6327(17) Å, *V=*1327.3(3) Å^3^, *Z=*2, *ρ*_calcd_=1.517 mg cm^−3^, *μ*=9.021 mm^−1^, *T=*100(2) K, *θ* range 4.493–64.897°, 23 836 reflections collected, 2100 independent reflections (*R*_int_=0.1099), *R*1=0.1412, *wR*2=0.4059 (*I*>2σ(*I*)).

### Debundeling of chirality‐enriched (6,5)‐SWCNTs with 5 b

#### Master solution of 5 b (5 b_Master_)

The master solution **5 b_Master_** was prepared by dissolving **5 b** (1.86 mg) in chloroform (5 mL, *c*=6.57×10^−4^ mol L^−1^). Afterwards, the obtained solution was treated by bath sonication (320 W, 80 kHz) for 10 min.

#### Dispersion FC00

The mixture FC00 was obtained by diluting **5 b_Master_** (800 μL) in chloroform (20 mL, *c*=0.01488 mg mL^−1^).

### Dispersion of SWCNTs with 5 b

#### Dispersion FC01

The mixture FC01 was prepared by dispersing SWCNTs solid material (0.82 mg) in FC00 (6 mL) and applying bath sonication (320 W, 80 kHz) for 30 min, followed by centrifugation (30 min, 30 000 g).

#### Dispersion FC02

The mixture FC02 was prepared by dispersing SWCNTs solid material (0.75 mg) in FC00 (6 mL) and applying tip sonication (5 s/55 s, 80 W) for 30 min, followed by centrifugation (30 min, 30 000 g).

Dispersions FC01 and FC02 were prepared with a SWCNT solid content of (0.128±0.009) mg mL^−1^.

### Reference dispersion of SWCNTs

#### Dispersion SWCNTs (CHCl_3_)

The reference dispersion SWCNTs (CHCl_3_) was obtained by pouring SWCNTs into CHCl_3_ and employing bath sonication (320 W, 80 kHz) for 30 min followed by centrifugation (30 min, 30 000 g). The SWCNT solid content was kept to (0.128±0.009) mg mL^−1^.

#### Dispersion SWCNTs (H_2_O)

The reference dispersion of SWCNTs (H_2_O) was obtained by adapting previously reported sonication procedures.^30^, ^118^, ^119^ The protocol comprised addition of a solution of widely known surfactants with documented effective debundeling ability (4 parts sodium deoxycholate (DOC) with 1 part sodium dodecyl sulfate (SDS) at a total aqueous surfactant concentration of 1 wt %) to the SWCNT solid material, followed by applying tip sonication (5 s/55 s, 80 W) for 30 min, followed by centrifugation (2 h, 55 000 g). The SWCNT solid content was kept to 0.040 mg mL^−1^.

### Debundeling of NanoIntegris IsoSol S‐100® SWCNT dispersion with 5 b

The commercial SWCNT dispersion, stabilized by a 9‐(9,9‐dihexyl‐9 *H*‐fluoren‐2‐yl)aryl‐based polymer (PFH‐R)^100^ and using toluene as the solvent base, was vacuum filtrated (2 mL) by using a Merck OmniPore JV micropore membrane filter (pore size 0.1 μm, Lot Nr. R7NA68631) in a standard vacuum filtration setup^29^ to produce a solid bucky paper on the filter fabric. The SWCNT solid was then first washed with toluene (20 mL) to achieve a viable depletion of the PFH‐R polymer around the SWCNTs^29^ and consequently flushed with **5 b** dissolved in toluene (5 mL, *c*=1.314×10^−3^ mol L^−1^) to attach **5 b** at those parts of the SWCNT sidewalls depleted from PFH‐R. To apply UV/Vis/NIR analysis for this step, the bucky paper obtained this way was manually delaminated from the filter by using standard lab cutlery. The slices of the SWCNT solid were then collected in a new glass vial (10 mL screw‐cap) and re‐dispersed in toluene (2 mL) by using a bath sonicator (20 min, 40 °C, 37 Hz, 200 W). The obtained liquid obtained by this procedure (**Gen2**) was subjected to the UV/Vis/NIR analysis and compared with basic spectra of **5 b** in toluene, just diluted NanoIntegris IsoSol S‐100® (**Gen0**) and a reference sample obtained directly by dissolving a bucky paper derived from NanoIntegris IsoSol S‐100® without the washing (toluene) and flushing (**5 b** in toluene) steps (**Gen1**).

### Associated Content

Additional structural data, cyclic voltammograms, square wave voltammograms, and UV/Vis/NIR spectra as well as spectroscopic details (^1^H and ^13^C{^1^H} NMR spectra) for all new compounds are given. This material is available free of charge via the Internet. CCDC https://www.ccdc.cam.ac.uk/services/strctures?id=doi:10.1002/chem.201904450 contain the supplementary crystallographic data for this paper. These data are provided free of charge by http://www.ccdc.cam.ac.uk/.

## Conflict of interest

The authors declare no conflict of interest.

## Supporting information

As a service to our authors and readers, this journal provides supporting information supplied by the authors. Such materials are peer reviewed and may be re‐organized for online delivery, but are not copy‐edited or typeset. Technical support issues arising from supporting information (other than missing files) should be addressed to the authors.

Supplementary
