# Supplementary material for: Ferrocenyl‐Pyrenes, Ferrocenyl‐9,10‐Phenanthrenediones, and Ferrocenyl‐9,10‐Dimethoxyphenanthrenes: Charge‐Transfer Studies and SWCNT Functionalization
Source: Chemistry. 2020 Jan 23;26(12):2635–52. doi: 10.1002/chem.201904450 (PMC7064959; doi:10.1002/chem.201904450)
Supplement: Supplementary file 1 — Supplementary [file CHEM-26-2635-s001.pdf]

CHEMISTRY
A European Journal

Supporting Information

Ferrocenyl-Pyrenes, Ferrocenyl-9,10-Phenanthrenediones, and Ferrocenyl-9,10-Dimethoxyphenanthrenes: Charge-Transfer Studies and SWCNT Functionalization**

Andrea Preuß,[a] Sebastian Notz,[a] Eduard Kovalski,[a] Marcus Korb,[a, b] Thomas Blaudeck,[c, d] Xiao Hu,[c, d] Jörg Schuster,[c, d, e] Dominique Miesel,[a] Tobias Rüffer,[a] Alexander Hildebrandt,[a] Katja Schreiter,[f] Stefan Spange,[f] Stefan E. Schulz,[c, d, e] and Heinrich Lang*[a, e]

chem_201904450_sm_miscellaneous_information.pdf

Content:

	Synthesis of 9-ferrocenylphenanthrene	pS4
Table SI1	Selected D-Fe bond lengths, angles and torsion angles of **5a**,**b** and **7a**,**b**	pS5
Table SI2	Selected D-Fe bond lengths, angles and torsion angles of **9-Fc-Phen**, **3**, **10** and **12**	pS5
Scheme SI1	Numbering schemes of phenanthrene and pyrene backbone	pS6
Table SI3	Bond lengths of pyrene substituents of **3**, **5a**–**b** and **7a**–**b**	pS6
Table SI4	Bond lengths of phenanthrene substituents of **9-Fc-Phen**, **10** and **12**	pS6
Table SI5	RMS deviations of the aromatic planes of compounds **3**, **5a**,**b**, **7a**,**b**, **9-FcPhen**, **10** and **12**	pS7
Figure SI1	ORTEP (50 % probability level) of the molecular structures of **9-FcPhen**	pS7
Figure SI2	ORTEP (50 % probability level) of the molecular structure of **3**	pS7
Figure SI3	ORTEP (50 % probability level) of the molecular structures of **5a**	pS8
Figure SI4	ORTEP (50 % probability level) of the molecular structure of **7a**	pS8
Figure SI5	ORTEP showing the intermolecular *T*-shaped π-interaction of **9-Fc-phen**	pS9
Figure SI6	ORTEP showing the intermolecular *T*-shaped π-interaction of **3**	pS9
Figure SI7	ORTEP showing the intermolecular parallel displaced π-interaction of **5a**	pS10
Figure SI8	ORTEP showing the intermolecular *T*-shaped π-interaction of **5b**	pS11
Figure SI9	ORTEP showing the intermolecular parallel displaced π-interactions of **7a**	pS11
Figure SI10	ORTEP showing the intermolecular *T*-shaped π-interaction of **7a**	pS12
Figure SI11	ORTEP showing the intermolecular parallel displaced π-interactions of **10**	pS13
Figure SI12	ORTEP showing the intermolecular parallel displaced π-interactions of **12**	pS13
Table SI6	Solvent polarity parameter sets of Kamlet–Taft: HBD ability α, HBA ability β, and dipolarity/polarizability π^*	pS14
Figure SI13	Cyclic and square wave voltammograms of **9-FcPhen** and **3**	pS15
Table SI7	CV data of **9-FcPhen**	pS15
Figure SI14	Cyclic and square wave voltammograms of **5a** and **7a**	pS16
Figure SI15	Deconvoluted square wave voltammograms of **7b**	pS16
Figure SI16	Deconvoluted square wave voltammograms of **10** and **12**	pS17
Scheme SI2	Resonance structures illustrating the through-bond charge transfer pathways in [**5b**]$^+$, [**7b**]$^+$, [**10**]$^+$ and [**12**]$^+$	pS17
Figure SI17	UV/vis/NIR spectra and deconvolution of **9-FcPhen**	pS18
Table SI8	NIR data of **9-FcPhen**	pS18
Figure SI18	UV/vis/NIR spectra and deconvolution of **3**	pS18
Figure SI19	UV/vis/NIR spectra and deconvolution of **5a**	pS19
Figure SI20	Deconvolution of the NIR spectra of [**5b**]$^+$	pS19
Figure SI21	Deconvolution of the NIR spectra of [**5b**]$^{2+}$	pS19
Figure SI22	UV/vis/NIR spectra and deconvolution of **7a**	pS20
Figure SI23	UV/vis/NIR spectra and deconvolution of **10**	pS20
Figure SI24	UV/vis/NIR spectra and deconvolution of **12** in dichloromethane	pS21

Figure SI25	UV/vis/NIR spectra and deconvolution of **12** in propylene carbonate	pS22
	Disentangling Experiments of chirality-enriched (*6,5*)-SWCNTs with **5b**	pS23
Figure SI26	UV/vis/NIR spectra of SWCNTs dispersed in chloroform in presence of **5b** as debundeling agent. Reference spectra of the same SWCNT batch in aqueous environment is given.	pS23
Figure SI27	Cyclic voltammogramm of **5b** (potential area –1250 to 1250 mV, scan rate 100 mV s^{-1}).	pS25
Figure SI28	Cyclic voltammogramms of cycles 2 to 5 of the ferrocenyl-based oxidation of **Gen2** (potential area: 100 to 600 mV) before (left) and after (right) the pyrene oxidation.	pS25
Figure SI29	^{1}H NMR spectrum of **9-FcPhen** in $CDCl_3$	pS26
Figure SI30	^{13}C NMR spectrum of **9-FcPhen** in $CDCl_3$	pS26
Figure SI31	^{1}H NMR spectrum of **5a** in $CDCl_3$	pS27
Figure SI32	^{13}C NMR spectrum of **5a** in $CDCl_3$	pS27
Figure SI33	^{1}H NMR spectrum of **5b** in $CDCl_3$	pS28
Figure SI34	^{13}C NMR spectrum of **5b** in $CDCl_3$	pS28
Figure SI35	^{1}H NMR spectrum of **7a** in $CDCl_3$	pS29
Figure SI36	^{13}C NMR spectrum of **7a** in $CDCl_3$	pS29
Figure SI37	^{1}H NMR spectrum of **7b** in $CDCl_3$	pS30
Figure SI38	^{1}H NMR spectrum of **10** in $CDCl_3$	pS30
Figure SI39	^{13}C NMR spectrum of **10** in $CDCl_3$	pS31
Figure SI40	^{1}H NMR spectrum of **12** in $CDCl_3$	pS31
Figure SI41	^{13}C NMR spectrum of **12** in $CDCl_3$	pS32

Synthesis of 9-ferrocenylphenanthrene (9-FcPhen)

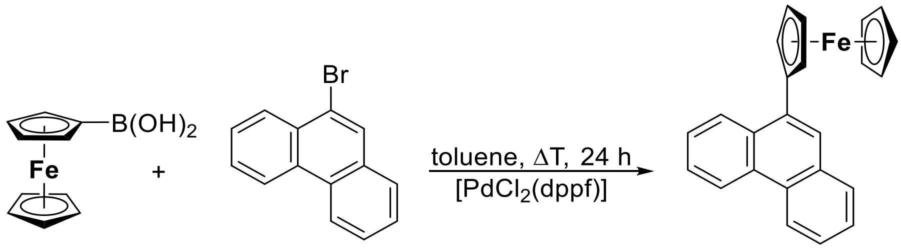

(1)

A three-necked 100 mL flask was charged with [Pd(dppf)Cl_2] (1 mol-%), ferrocene boronic acid (**1**) (196 mg, 0.85 mmol, 1.2 equiv), $K_3PO_4 \cdot H_2O$ (589 mg, 2.6 mmol, 2.5 equiv) and 9-bromophenanthrene (182.8 mg, 0.71 mmol). Anhydrous toluene (15 mL) was added. The reaction mixture was stirred for 5 min at ambient temperature and then heated to reflux for 24 h. After cooling the reaction mixture to ambient temperature, it was filtered through a pad of silica. Afterwards, all volatiles were removed by evaporation. Purification was realized by column chromatography (silica, column size 4 x 25 cm). Compound **9-FcPhen** was separated by using hexane/dichloromethane eluent mixtures (*v*/*v*) starting from 9:1 (ferrocene, 98 mg, 0.53 mmol; 74 % based on 1-bromophenanthrene) to 4:1 (9-ferrocenylphenanthrene). After evaporation of all volatiles, 9-ferrocenylphenanthrene was obtained as an orange solid.

Yield: 79.2 mg (0.22 mmol, 31 % based on 9-bromophenanthrene). m.p. 127 °C; ^{1}H NMR ($CDCl_3$, δ): 4.25 (s, 5H, C_5H_5), 4.44 (t, J_{HH} = 1.8 Hz, 2H, C_5H_4), 4.70 (t, J_{HH} = 1.8 Hz, 2H, C_5H_4), 7.58–7.68 (m, 4H, $C_{14}H_9$), 7.88–7.91 (m, 1H, $C_{14}H_9$), 8.14 (s, 1H, $C_{14}H_9$), 8.66 (dd, J_{HH} = 12.8 Hz, 4.7 Hz, 2H, $C_{14}H_9$), 8.75 (dd, J_{HH} = 8.5 Hz, 1 Hz, 1H, $C_{14}H_9$); ^{13}C NMR ($CDCl_3$, δ): 68.4 (C_5H_4), 69.8 (C_5H_5), 70.7 (C_5H_4), 87.6 (^{q}C, C_5H_4), 122.7 ($C_{14}H_9$), 123.0 ($C_{14}H_9$), 126.2 ($C_{14}H_9$), 126.3 ($C_{14}H_9$), 126.4 ($C_{14}H_9$), 126.9 ($C_{14}H_9$), 127.0 ($C_{14}H_9$), 128.4 ($C_{14}H_9$), 128.7 ($C_{14}H_9$), 129.8 ($C_{14}H_9$), 130.6 ($C_{14}H_9$) 131.5 ($C_{14}H_9$), 131.8 ($C_{14}H_9$), 134.5 ($C_{14}H_9$); IR (KBr, v/cm^{-1}): 2955 (m), 2925 (s), 2854 (m), 1652 (w), 1634 (w), 1592 (w), 1526 (w), 1494 (m), 1451 (m), 1435 (m), 1410 (m), 1373 (m), 1322 (w), 1173 (w), 1106 (m), 1042 (m), 999 (m), 948 (m), 902 (m), 884 (w), 837 (m), 822 (s), 770 (s), 749 (s), 729 (s); 668 (m); HR-MS (ESI-TOF, *m/z*). calcd for $C_{24}H_{18}Fe$ 362.0753, found 362.0801 $[M]^+$; elemental analysis calcd for $C_{24}H_{18}Fe$ (362.25 g/ mol): C 79.58, H 5.01, found: C 79.88, H 5.49.

Crystal data for 9-ferrocenylphenanthrene: $C_{24}H_{18}Fe$, M_r = 362.23 g/mol, orthorhombic, $P2_12_12_1$, λ = 0.71073 Å, a = 7.2716(3) Å, b = 12.9857(6) Å, c = 17.1469(8) Å, V = 1619.13(13) Å^3, Z = 4, ρ_{calcd} = 1.486 mg cm^{-3}, μ = 0.933 mm^{-1}, T = 115.00(14) K, Θ range 3.043 – 24.995°, 6243 reflections collected, 2826 independent reflections (R_{int} = 0.0402), R_1 = 0.0381, wR_2 = 0.0655 ($I > 2\sigma(I)$).

Table SI1. Selected D-Fe bond lengths, angles and torsion angles of **5a**,**b** and **7a**,**b**.

	**3**	**5a**	**5b**	**7a**	**7b**
D1–Fe1 (Å)	1.6398(3)	1.6485(4)	1.6467(13)	1.6500(3)	1.6436(4)
D2–Fe1 (Å)	1.6486(3)	1.6524(4)	1.6549(13)	1.6550(3)	1.6513(4)
D3–Fe2 (Å)			1.6608(14)		
D4–Fe2 (Å)			1.6566(14)		
D1–Fe1–D2 (°)	177.90(2)	179.01(3)	178.38(8)	177.92(2)	177.05(3)
D3–Fe2–D4 (°)			178.79(8)		
C_5H_4(D1)–C_6(C11) (°)	37.02(8)	40.05(10)		17.66(12)	20.53(16)
C_5H_4(D1)–C_6(C21) (°)			31.9(4)		
C_5H_4(D2)–C_6(C28) (°)			31.5(5)		
C1–D1–D2–C6 (°)	–0.07(14)	8.9(2)	4.0(7)	1.63(16)	3.2(2)
C11–D3–D4–C16 (°)			–3.5(7)		

D1 = centroid of C1-C5; D2 = centroid of C6-C10; D3 = C11-C15; D4 = C16-C20.

Table SI2. Selected D-Fe bond lengths, angles and torsion angles of **9-FcPhen**, **3**, **10** and **12**.

	**9-FcPhen**	**10**	**12**
D1–Fe1 (Å)	1.6479(6)	1.6435(8)	1.64(6)
D2–Fe1 (Å)	1.6484(6)	1.6620(8)	1.64(6)
D3–Fe2 (Å)		1.6460(8)	
D4–Fe2 (Å)		1.6585(8)	
D5–Fe3 (Å)		1.6375(8)	
D6–Fe3 (Å)		1.6660(8)	
D7–Fe4 (Å)		1.6556(8)	
D8–Fe4 (Å)		1.6557(8)	
D1–Fe1–D2 (°)	176.40(4)	177.81(6)	179(5)
D3–Fe2–D4 (°)		178.58(6)	
D5–Fe3–D6 (°)		178.17(6)	
D7–Fe4–D8 (°)		178.43(6)	
C_5H_4(D1)–C_6(C11) (°)	38.68(15)		22.1(9)
C_5H_4(D1)–C_6(C21) (°)		2.3(3)	
C_5H_4(D3)–C_6(C30) (°)		0.1(3)	
C_5H_4(D5)–C_6(C61) (°)		2.8(3)	
C_5H_4(D7)–C_6(C70) (°)		1.7(3)	
C1–D1–D2–C6 (°)	–5.3(3)	1.7(4)	13(3)
C1–D1–D2–C10 (°)			
C11–D3–D4–C16 (°)		6.4(4)	
C41–D5–D6–C46 (°)		–5.5(4)	
C51–D7–D8–C60 (°)		–3.2(4)	

D1 = centroid of C1-C5; D2 = centroid of C6-C10; D3 = C11-C15; D4 = C16-C20; D5 = C41-C45; D6 = C46-C50; D7 = C51-C55, D8 = C56-C60.

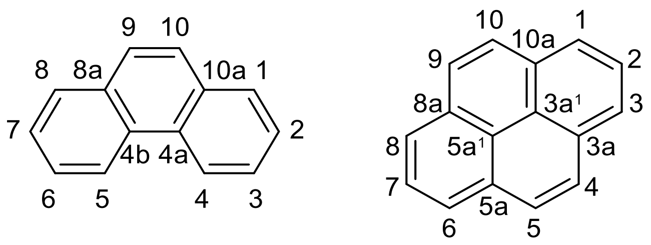

Scheme SI1. Numbering schemes of phenanthrene and pyrene.

Table SI3. Bond lengths of pyrene substituents of **3**, **5a**–**b** and **7a**–**b**.

	**3**	**5a**	**5b**	**7b**	**7a**
C1–C2 (Å)	1.392(3)	1.402(4)	1.413(13)	1.400(4)	1.399(3)
C2–C3 (Å)	1.375(3)	1.374(4)	1.378(12)	1.398(4)	1.396(3)
C3–C3a (Å)	1.394(3)	1.393(4)	1.378(12)	1.388(4)	1.398(3)
C3a–C3a^{1} (Å)	1.419(3)	1.420(4)	1.419(12)	1.428(4)	1.422(3)
C3a–C4 (Å)	1.430(3)	1.431(4)	1.438(12)	1.441(4)	1.428(3)
C3a^{1}–C5a^{1} (Å)	1.425(3)	1.433(4)	1.423(11)		1.416(3)
C4–C5 (Å)	1.347(3)	1.344(4)	1.343(12)		1.353(3)
C5–C5a (Å)	1.437(3)	1.438(4)	1.420(13)		1.433(3)
C5a–C5a^{1} (Å)	1.420(3)	1.431(4)	1.430(11)		1.420(3)
C5a–C6 (Å)	1.399(3)	1.398(4)	1.429(12)		1.394(3)
C6–C7 (Å)	1.375(3)	1.379(4)	1.399(13)		1.383(3)
C7–C8 (Å)	1.377(3)	1.380(4)	1.384(12)		1.384(3)
C8–C8a (Å)	1.390(3)	1.395(4)	1.421(12)		1.400(3)
C8a–C5a^{1} (Å)	1.418(3)	1.422(4)	1.422(13)		1.423(3)
C8a–C9 (Å)	1.428(3)	1.428(4)	1.429(12)		1.433(3)
C9–C10 (Å)	1.349(3)	1.348(4)	1.365(12)		1.347(3)
C10–C10a (Å)	1.439(3)	1.439(4)	1.431(13)	1.437(4)	1.443(3)
C10a–C3a^{1} (Å)	1.436(3)	1.433(4)	1.459(11)	1.425(4)	1.416(3)
C10a–C1 (Å)	1.410(3)	1.411(4)	1.433(12)	1.400(4)	1.391(3)

Table SI4. Bond lengths of phenanthrene substituents of **9-FcPhen**, **10** and **12**.

	**9-FcPhen**	**10 (1)**	**10 (2)**	**12**
C1–C2 (Å)	1.356(6)	1.371(8)	1.360(8)	1.38(4)
C2–C3 (Å)	1.402(6)	1.401(7)	1.400(7)	1.50(3)
C3–C4 (Å)	1.368(6)	1.403(7)	1.404(7)	1.31(3)
C4–C4a (Å)	1.400(6)	1.390(7)	1.386(7)	1.40(3)
C4a–C4b (Å)	1.457(6)	1.480(7)	1.491(7)	
C4a–C10a (Å)	1.416(5)	1.407(7)	1.402(7)	1.52(3)
C4b–C5 (Å)	1.404(6)	1.391(7)	1.379(7)	
C4b–C8a (Å)	1.420(6)	1.412(7)	1.401(7)	
C5–C6 (Å)	1.372(6)	1.412(7)	1.405(7)	
C6–C7 (Å)	1.392(6)	1.388(7)	1.408(7)	
C7–C8 (Å)	1.363(6)	1.374(7)	1.362(8)	
C8–C8a (Å)	1.422(6)	1.394(7)	1.416(7)	
C8a–C9 (Å)	1.445(5)	1.466(7)	1.458(7)	
C9–C10 (Å)	1.360(5)	1.537(8)	1.542(8)	

C10–C10a (Å)	1.418(6)	1.463(7)	1.455(7)	1.48(4)
C10a–C1 (Å)	1.410(6)	1.407(7)	1.415(7)	1.33(3)

Table SI5. RMS (root mean square) deviations of the aromatic planes of compounds **3**, **5a**,**b**, **7a**,**b**, **9-ferrocenylphenanthrene**, **10** and **12** and the atom showing the highest displacement (d_{max} / Å).*)

Compd.	RMS	d_{max}
**3**	0.0348	0.0685(17) C11
**5a**	0.0497	–0.041(2) C18
**5b**	0.0418	0.087(8) C29
**7a**	0.0092	0.0157(19) C19
**7b**	0.0072	0.011(2) C18
**9-FcPhen**	0.0453	0.082(4) C20
**10**	0.0089	0.022(4) C25
**12**	0.0680	0.089(16) C11

*) The values were calculated by using WinGX.

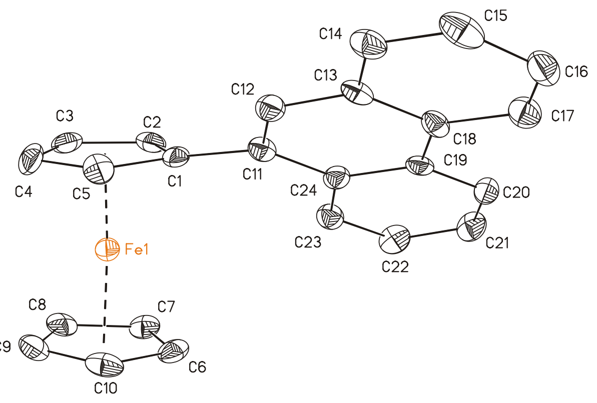

Figure SI1. ORTEP Diagramm (50 % probability level) of the molecular structure of **9-FcPhen** with the atom numbering scheme. All hydrogen atoms have been omitted for clarity. Selected bond distances (Å) and angles (deg), and torsion angles (deg): C1–C11 = 1.482(5), C2–C1–C11 = 130.1(4), C5–C1–C11 = 123.0(4), C12–C11–C1 = 116.6(4), C24–C11–C1 = 124.8(4), C11–C1–C2–C3 = –168.9(4), C11–C1–C5–C4 = 170.0(4), C2–C1–C11–C12 = 134.6(5), C5–C1–C11–C12 = –34.3(6), C1–C11–C12–C13 = –179.3(4), C5–C1–C11–C24 = 147.2(4), C2–C1–C11–C24 = –43.9(7).

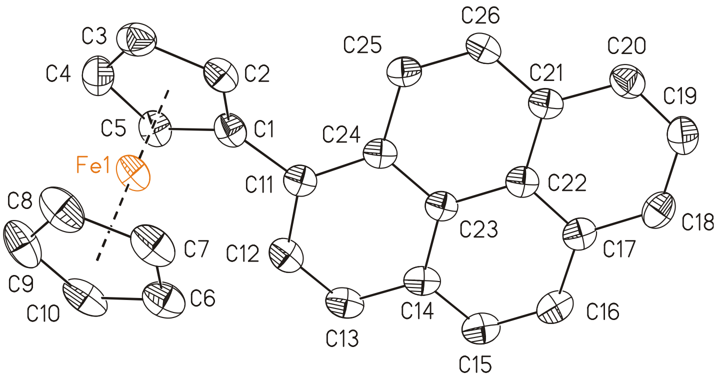

Figure SI2. ORTEP Diagramm (50 % probability level) of the molecular structure of **3** with the atom numbering scheme. All hydrogen atoms have been omitted for clarity. Selected bond distances (Å) and angles (deg), and torsion angles (deg): C1–C11 = 1.482(3), C2–C1–C11 = 129.03(18), C5–C1–C11 = 124.2(2), C12–C11–C1 = 119.35(19), C24–C11–C1 = 122.28(19), C11–C1–C2–C3 = –177.3(2), C11–

C1–C5–C4 = 177.1(2), C2–C1–C11–C12 = 141.0(2), C5–C1–C11–C12 = –35.3(3), C1–C11–C12–C13 = –177.88(19), C5–C1–C11–C24 = 144.8(2), C2–C1–C11–C24 = –38.9(3).

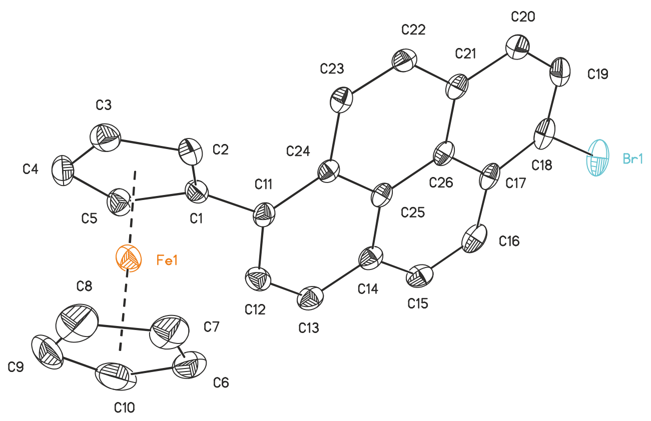

Figure SI3. ORTEP (50 % probability level) of the molecular structure of **5a** with the atom numbering scheme. All hydrogen atoms have been omitted for clarity. Selected bond distances (Å) and angles (deg), and torsion angles (deg): C1–C11 = 1.485(4), C18–Br1 = 1.912(3), C5–C1–C11 = 122.7(2), C2–C1–C11 = 131.0(2), C12–C11–C1 = 117.9(2), C24–C11–C1 = 123.3(2), C19–C18–Br1 = 117.5(2), C17–C18–Br1 = 119.5(2), C11–C1–C2–C3 = 177.6(3), C11–C1–C5–C4 = –178.0(2), C5–C1–C11–C12 = -38.1(4), C2–C1–C11–C12 = 143.9(3), C5–C1–C11–C24 = 137.2(3), C2–C1–C11–C24 = –40.8(4), C1–C11–C12–C13 = 174.7(3), C1–C11–C24–C25 = –174.9(2), C1–C11–C24–C23 = 3.6(4), C26–C17–C18–Br1 = –176.35(19), C16–C17–C18–Br1 = 2.0(4), Br1–C18–C19–C20 = 177.0(2).

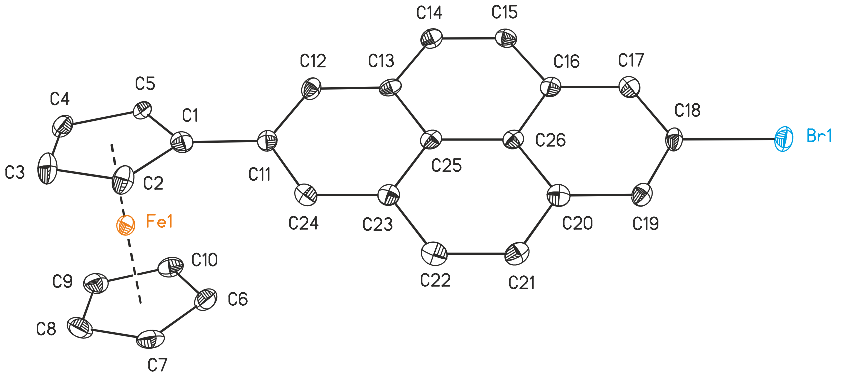

Figure SI4. ORTEP (50 % probability level) of the molecular structure of **7a** with the atom numbering scheme. All hydrogen atoms have been omitted for clarity. Selected bond distances (Å) and angles (deg), and torsion angles (deg): C1–C11 = 1.476(3), Br1–C18 = 1.902(2), C5–C1–C11 = 126.3(2), C2–C1–C11 = 126.4(2), C12–C11–C1 = 120.9(2), C24–C11–C1 = 120.4(2), C17–C18–Br1 = 118.11(18), C19–C18–Br1 = 119.39(16), C11–C1–C2–C3 = –178.2(2), C11–C1–C5–C4 = 177.9(2), C2–C1–C11–C12 = –163.4(2), C5–C1–C11–C12 = 18.7(4), C2–C1–C11–C24 = 16.7(4), C5–C1–C11–C24 = –161.2(2), C1–C11–C12–C13 = –179.0(2), C1–C11–C24–C23 = 179.8(2), C16–C17–C18–Br1 = 178.56(16), Br1–C18–C19–C20 = –179.48(16).

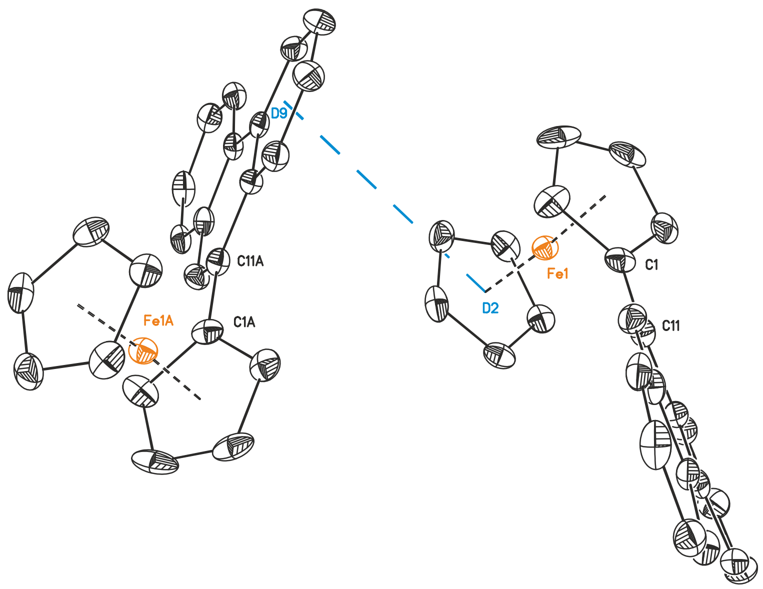

	d (Å)	α (°)
D2 ··· D9	4.981(3)	78.3(2)

Figure SI5. ORTEP (50 % probability level) of the molecular structure of **9-FcPhen** with a selected atom numbering scheme, showing the intermolecular *T*-shaped π-interaction (blue dashed lines) between the C_5H_5 units and C19-C24 unit forming a dimer. All hydrogen atoms have been omitted for clarity. (Symmetry code for generating-equivalent atoms; A:–x, y–1/2, 1/2–z).

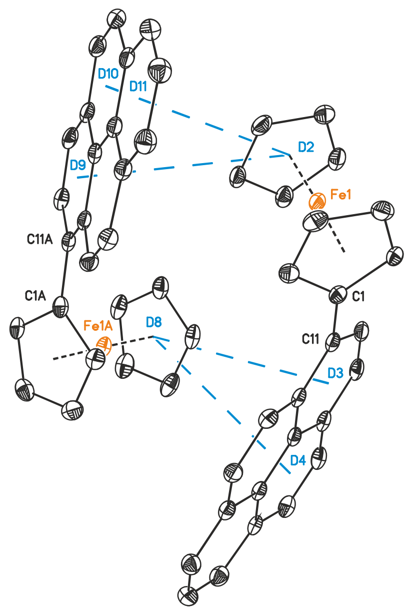

	d (Å)	α (°)
D2 ··· D9	4.5501(14)	79.44(12)
D2 ··· D10	4.5276(14)	79.64(12)
D3 ··· D8	4.5501(14)	79.44(12)
D4 ··· D8	4.5276(14)	79.64(12)

Figure SI6. ORTEP (30 % probability level) of the molecular structure of **3** with selected atom numbering scheme, showing the intermolecular *T*-shaped π-interaction (blue dashed lines) between the C_5H_5 unit

and the C_6H_4 moiety of pyrenyl forming a dimer. All hydrogen atoms have been omitted for clarity. (Symmetry code for generating-equivalent atoms; A: –x, y, 5/2–z).

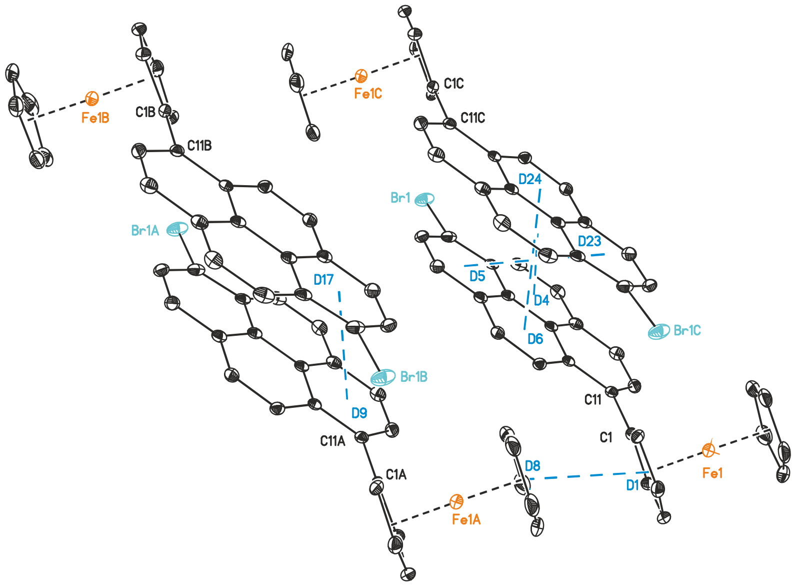

	d (Å)	α (°)
D1 ··· D8	4.894(2)	1.78(19)
D4 ··· D24	4.5778(16)	1.82(13)
D5 ··· D23	4.8119(17)	0
D6 ··· D24	3.8498(15)	0
D9 ···D17	4.5822(16)	2.14(13)
D1 ···D17	4.6081(17)	84.68(15)

Figure SI7. ORTEP (30 % probability level) of the molecular structure of **5a** with selected atom numbering scheme, showing the intermolecular parallel displaced π-interaction (blue dashed lines) between the C_5H_5 units and the pyrenyl moieties. All hydrogen atoms and further interactions (*T*-shaped) have been omitted for clarity. (Symmetry code for generating-equivalent atoms; A: x, y+1, z; B: 1/2–x, 3/2–y, 1/2–z; C: 1/2–x, 1/2–y, 1/2–z).

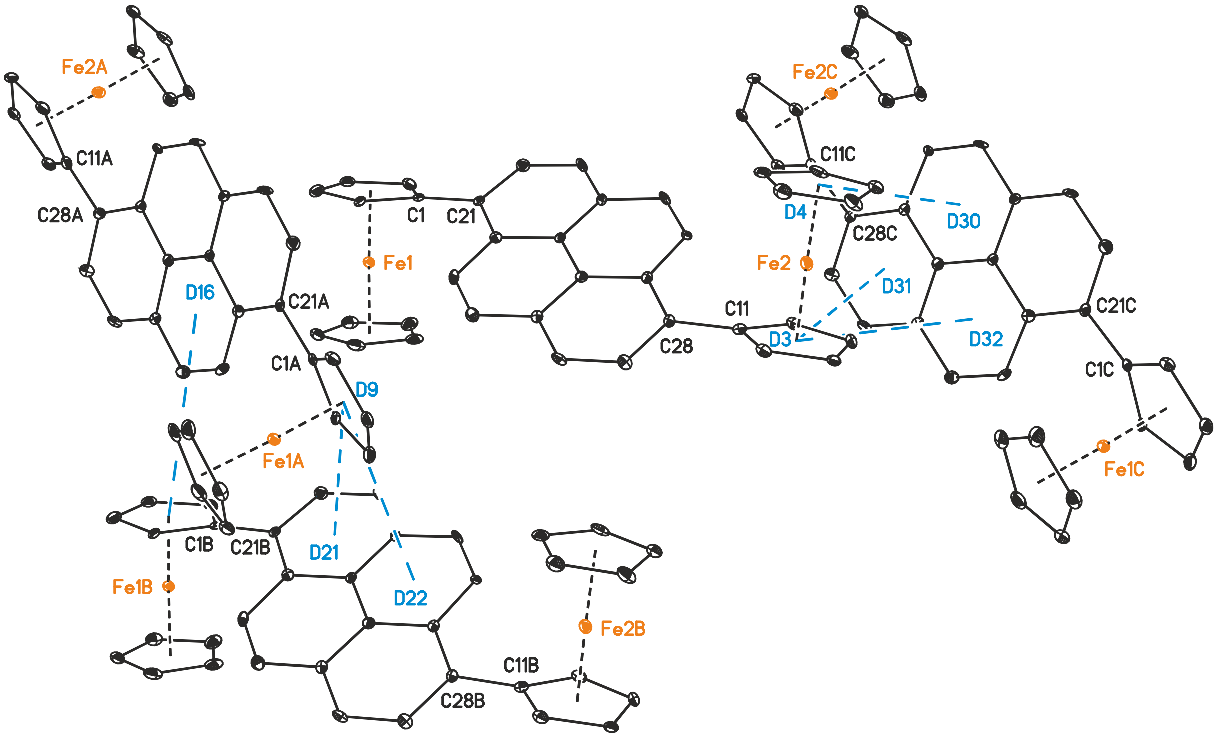

	d (Å)	α (°)
D3 ··· D31	4.678(6)	87.2(5)
D3 ··· D32	4.656(6)	87.3(5)
D4 ··· D30	4.718(6)	86.5(5)
D9 ··· D21	4.725(6)	86.6(5)
D9 ···D22	4.619(6)	85.6(5)
D16 ···D17	4.994(6)	86.7(5)

Figure SI8. ORTEP (30 % probability level) of the molecular structure of **5b** with selected atom numbering scheme, showing the intermolecular *T*-shaped π-interaction (blue dashed lines) between the C_5H_5 unit and the C_6H_4 moiety. All hydrogen atoms and further interactions have been omitted for clarity. (Symmetry code for generating-equivalent atoms; A: 5/2–x, y–1/2, 5/2–z; B: x, y–1, z; C: 7/2–x, y+1/2, 5/2–z).

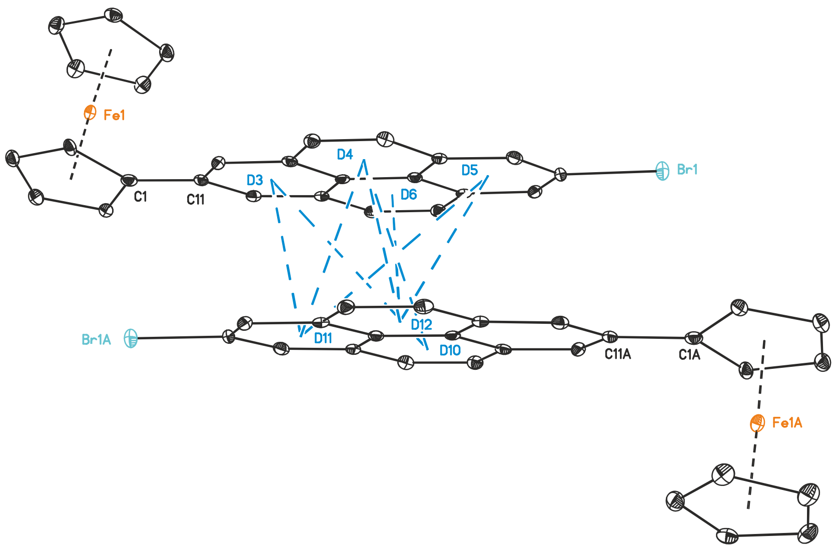

	d (Å)	α (°)	slippage
D3 ··· D10	4.6700(13)	0.85(10)	
D3 ··· D11	3.7590(13)	0.74(10)	
D4 ··· D10	3.7899(13)	0	1.695
D4 ··· D11	3.5535(13)	0.94(10)	
D4 ··· D12	3.7528(13)	0.69(10)	
D5 ···D11	4.7763(13)	0	3.370
D5 ···D12	4.2849(13)	0.30(10)	
D6 ···D12	5.0839(14)	0	3.792

Figure SI9. ORTEP (30 % probability level) of the molecular structure of **7a** with selected atom numbering scheme, showing the intermolecular parallel displaced π-interaction (blue dashed lines) between the pyrenyl units forming a dimer. All hydrogen atoms have been omitted for clarity. (Symmetry code for generating-equivalent atoms; A: 2–x, 1–y, 1–z).

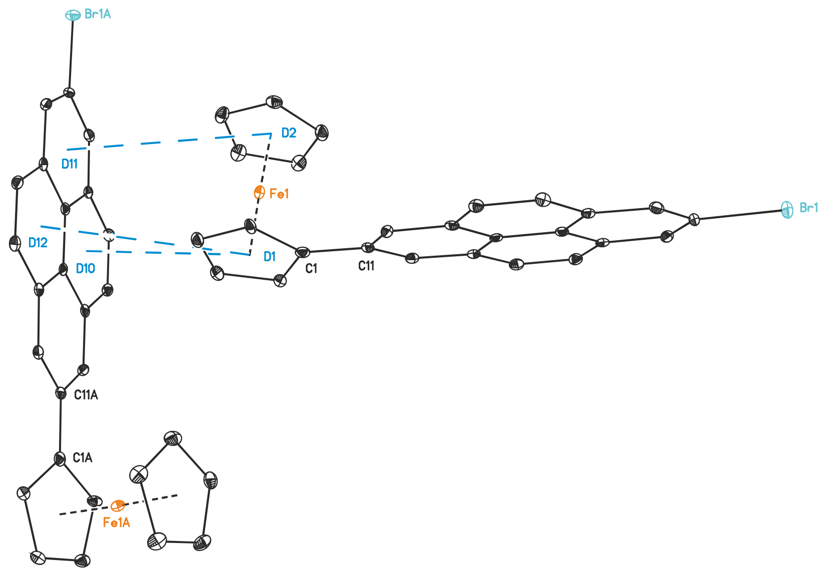

	d (Å)	α (°)
D1 ··· D10	4.6451(14)	89.24(12)
D1··· D12	4.6472(14)	89.21(12)
D2 ··· D11	4.6925(14)	87.13(13)

Figure SI10. ORTEP (30 % probability level) of the molecular structure of **7a** with selected atom numbering scheme, showing the intermolecular *T*-shaped π-interaction (blue dashed lines) between the C_5H_5 unit and the pyrenyl moiety forming a dimer. All hydrogen atoms have been omitted for clarity. DIMER!!!! (Symmetry code for generating-equivalent atoms; A: 1–x, y–1/2, 1/2–z).

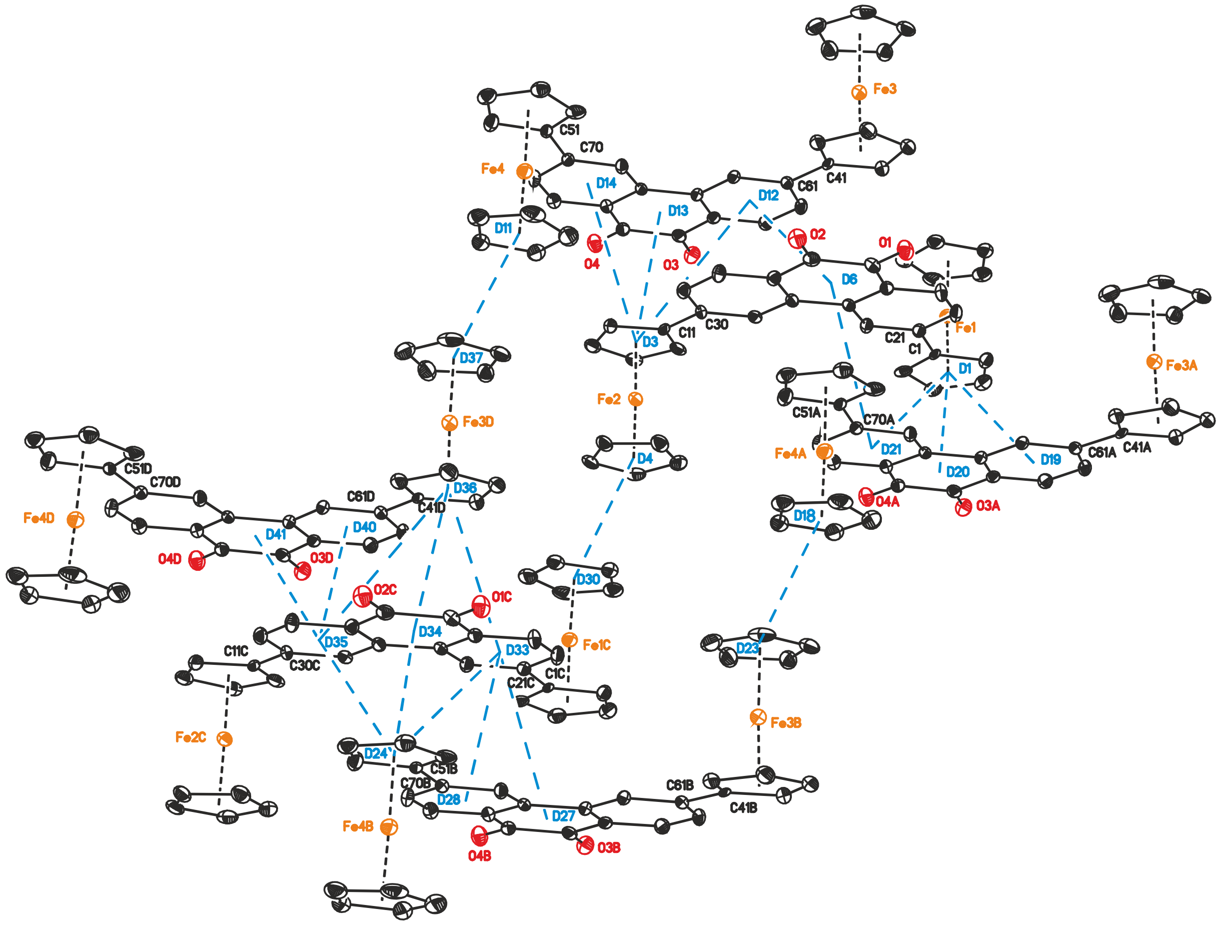

	d (Å)	α (°)	slippage

D1 ··· D19	4.201(3)	1.5(3)	
D1 ··· D20	3.424(3)	2.0(3)	
D1 ··· D21	4.736(3)	2.2(3)	
D3 ··· D12	4.799(3)	0.8(3)	
D3 ··· D13	3.521(3)	0.4(3)	
D3 ··· D14	4.465(3)	0.0(3)	3.007
D4 ··· D30	3.623(4)	2.0(4)	
D6 ···D12	4.452(3)	1.0(2)	
D6 ···D21	4.519(3)	0.4(2)	
D11 ··· D37	3.617(4)	1.8(4)	
D33 ··· D24	4.736(3)	1.6(3)	
D33 ··· D27	4.549(3)	0.5(2)	
D33 ··· D28	3.653(3)	0.0(3)	1.444
D33 ··· D36	4.278(3)	2.6(3)	
D34 ··· D24	3.478(3)	1.2(3)	
D34 ··· D36	3.466(3)	2.4(3)	
D35 ··· D24	4.390(3)	1.5(3)	
D35 ··· D36	4.800(3)	2.9(3)	
D35 ··· D40	3.589(3)	0.5(3)	
D35 ··· D24	4.420(3)	0.1(2)	

Figure SI11. ORTEP (30 % probability level) of the molecular structure of **10** with selected atom numbering scheme, showing the intermolecular parallel displaced π-interaction (blue dashed lines). All hydrogen atoms and further interactions have been omitted for clarity. (Symmetry code for generating-equivalent atoms; A: x+1, y, z; B: x+1, y, 1+z; C: x, y, 1+z; D: x, y, 1+z).

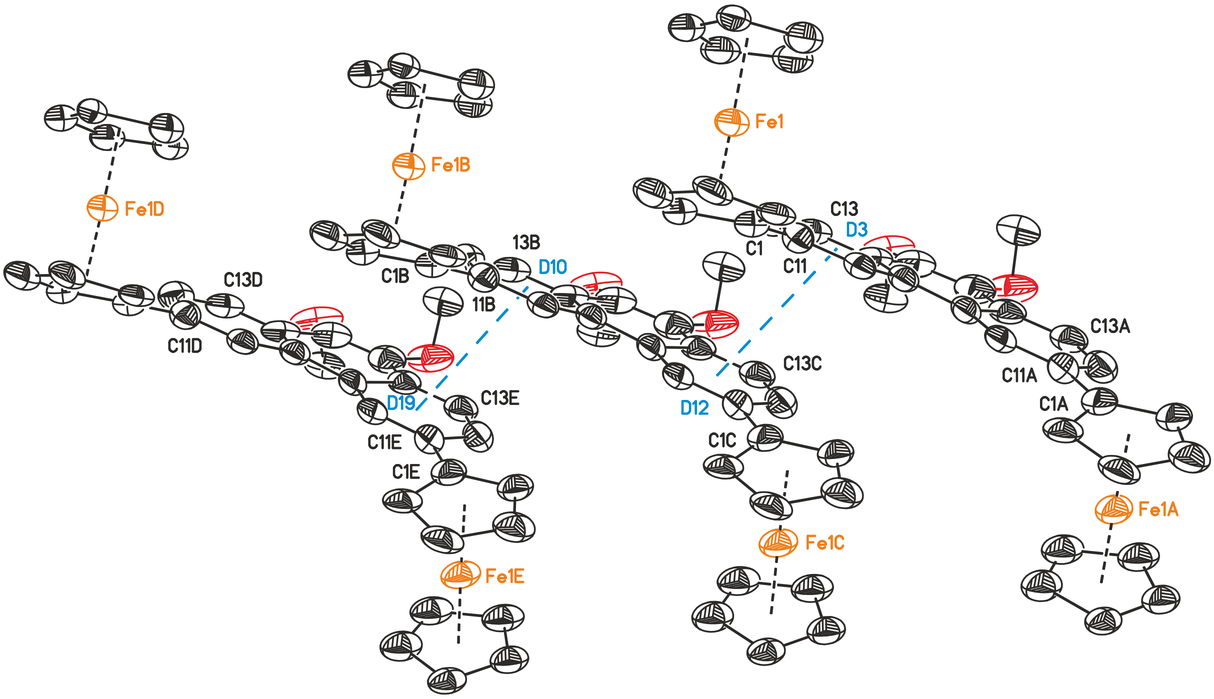

	d (Å)	α (°)
D3 ··· D12	5.165(12)	40
D10··· D19	5.165(12)	40
C13 ··· C13	3.17(3)	
C13 ··· C14	3.39(3)	
C16 ··· C16	3.04(3)	

Figure SI12. ORTEP (30 % probability level) of the molecular structure of **12** with selected atom numbering scheme, showing the intermolecular parallel displaced π-interaction (blue dashed lines) between the phenanthrenyl moieties. All hydrogen atoms and further interactions have been omitted for

clarity. (Symmetry code for generating-equivalent atoms; A: –x, –1–y, z; B: x, 1+y, z; C: –x, –y, z; D: x, 2+y, z; E: –x, 1–y, z).

Table SI6. Solvent polarity parameter sets of Kamlet–Taft:[1] HBD ability *α*, HBA ability *β*, and dipolarity/polarizability *π**.

Solvent	KAMLET–TAFT		
	α	*β*	*π**
n-hexane	0.00	0.00	-0.04
Cyclohexane	0.00	0.00	0.00
Triethylamine	0.00	0.71	0.14
diethyl ether	0.00	0.47	0.27
tetrachloromethane	0.00	0.10	0.28
p-xylene	0.00	0.12	0.43
1-decanol	0.70	0.82	0.45
1-butanol	0.84	0.84	0.47
2-propanol	0.76	0.84	0.48
1-propanol	0.84	0.90	0.52
1,2-dimethoxyethane	0.00	0.41	0.53
Toluene	0.00	0.11	0.54
Ethanol	0.86	0.75	0.54
ethyl acetate	0.00	0.45	0.55
1,4-dioxane	0.00	0.37	0.55
Tetrahydrofuran	0.00	0.55	0.58
Chloroform	0.20	0.10	0.58
Benzene	0.00	0.10	0.59
Methanol	0.98	0.66	0.60
1,1,1,3,3,3-hexafluoro-2-propanol	1.96	0.00	0.65
Acetone	0.08	0.43	0.71
Anisole	0.00	0.32	0.73
2,2,2-trifluorethanol	1.51	0.00	0.73
Acetonitrile	0.19	0.40	0.75
1,2-dichloroethane	0.00	0.10	0.81
Dichloromethane	0.13	0.10	0.82
Tetramethylurea	0.00	0.80	0.83
Nitromethane	0.22	0.06	0.85
4-butyrolactone	0.00	0.49	0.87
hexamethylphosphor-amide	0.00	1.05	0.87
Pyridine	0.00	0.64	0.87
N,*N*-dimethylacetamide	0.00	0.76	0.88
N,*N*-dimethylformamide	0.00	0.69	0.88
Benzonitrile	0.00	0.37	0.90
ethan-1,2-diol	0.00	0.00	0.95
1,1,2,2-tetrachloroethane	0.90	0.52	0.92
Formamide	0.71	0.48	0.97
dimethyl sulfoxide	0.00	0.76	1.00
Water	1.17	0.47	1.09

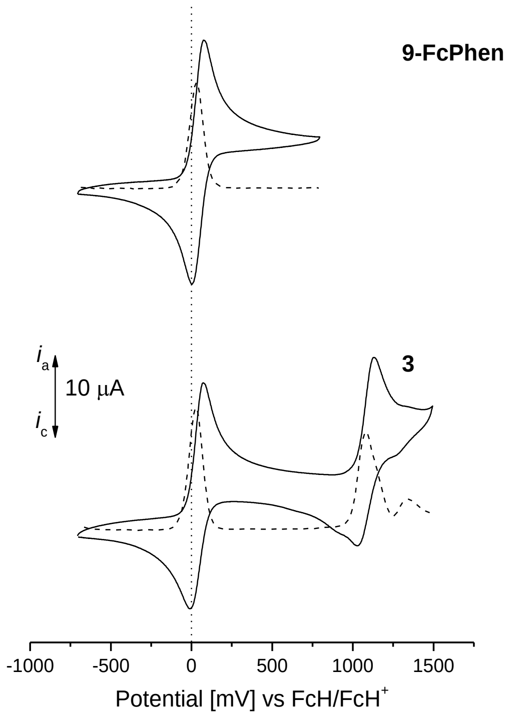

Figure SI13. Cyclic (solid lines) and square wave (dotted line) voltammograms (CV: potential area –1000 to 1750 mV; SW: potential area -1000 to 1750 mV) of **9-FcPhen** and **3**. Conditions: scan rate 100 mV s^{-1} (CV), 2.5 mV s^{-1} (SW) in dichloromethane solutions (1.0 mmol L^{-1}), supporting electrolyte 0.1 mol L^{-1} [N^nBu_4][$B(C_6F_5)_4$], working electrode glassy carbon.

Table SI7. CV Data of **9-FcPhen**. [a]

Compound	$E°_1{}'^b$ $(\Delta E_p)^c$	$E°_{aryl}{}'^b$ $(\Delta E_p)^c$
**9-FcPhen**	40 (74)	1470[d]

[a]Potentials vs FcH/FcH$^+$, scan rate 100 mV s^{-1}, at glassy carbon electrode of the analyte (1.0 mmol L^{-1} [N^nBu_4][$B(C_6F_5)_4$] as supporting electrolyte) in anhydrous dichloromethane at 25 °C. [b]$E°'$ = formal potential. [c]ΔE_p = difference between the oxidation and the reduction potential. [d]Values determined using square wave voltammetry.

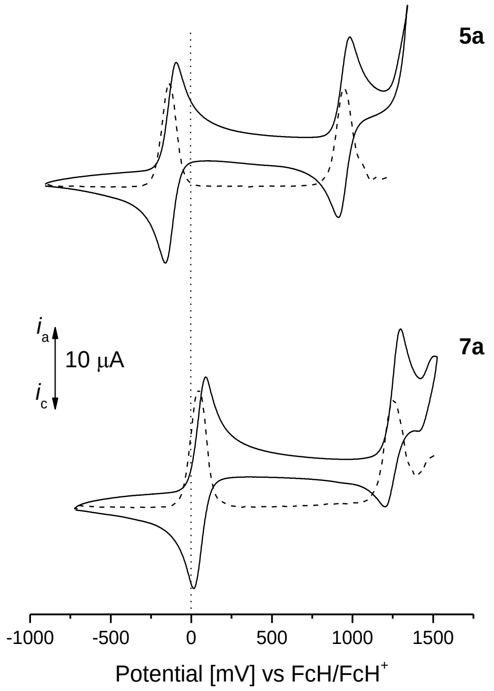

Figure SI14. Cyclic (solid line) and square wave (dotted line) voltammograms (CV: potential area -1000 to 1750 mV; SW: potential area -1000 to 1750 mV) of **5a** and **7a**. Conditions: scan rate 100 mV s^{-1} (CV), 2.5 mV s^{-1} (SW) in dichloromethane solutions (1.0 mmol L^{-1}), supporting electrolyte 0.1 mol L^{-1} [N^{n}Bu$_4$][B(C$_6$F$_5$)$_4$], working electrode glassy carbon.

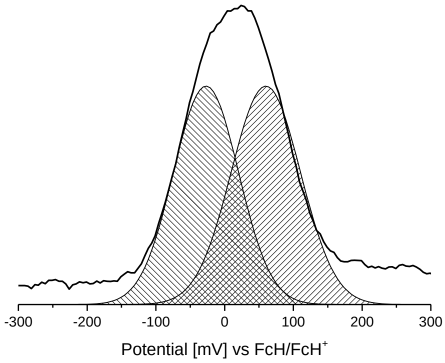

Figure SI15. Deconvolution of the square wave voltammograms (SW: potential area -300 to 300 mV) of **7b** to determine the redox separation according to the method of Richardson and Taube.[2] Conditions: 2.5 mV s^{-1} (SW) in dichloromethane solutions (0.1 mmol L^{-1}), supporting electrolyte 0.1 mol L^{-1} [N^{n}Bu$_4$][B(C$_6$F$_5$)$_4$], working electrode glassy carbon.

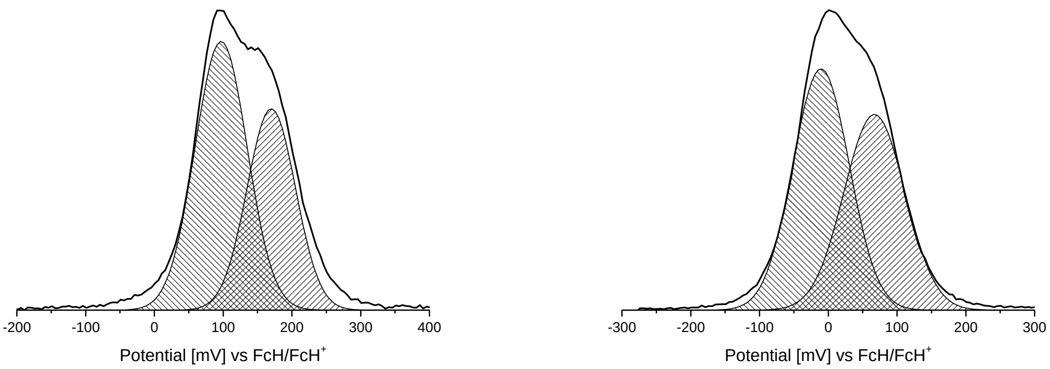

Figure SI16. Deconvolution of the square wave voltammograms (SW: potential area -1000 to 1750 mV) of **10** (left) and **12** (right) to determine the redox separation according to the method of Richardson and Taube.[2] Conditions: 2.5 mV s^{-1} (SW) in dichloromethane solutions (1.0 mmol L^{-1}), supporting electrolyte 0.1 mol L^{-1} [N^{n}Bu$_4$][B(C$_6$F$_5$)$_4$], working electrode glassy carbon.

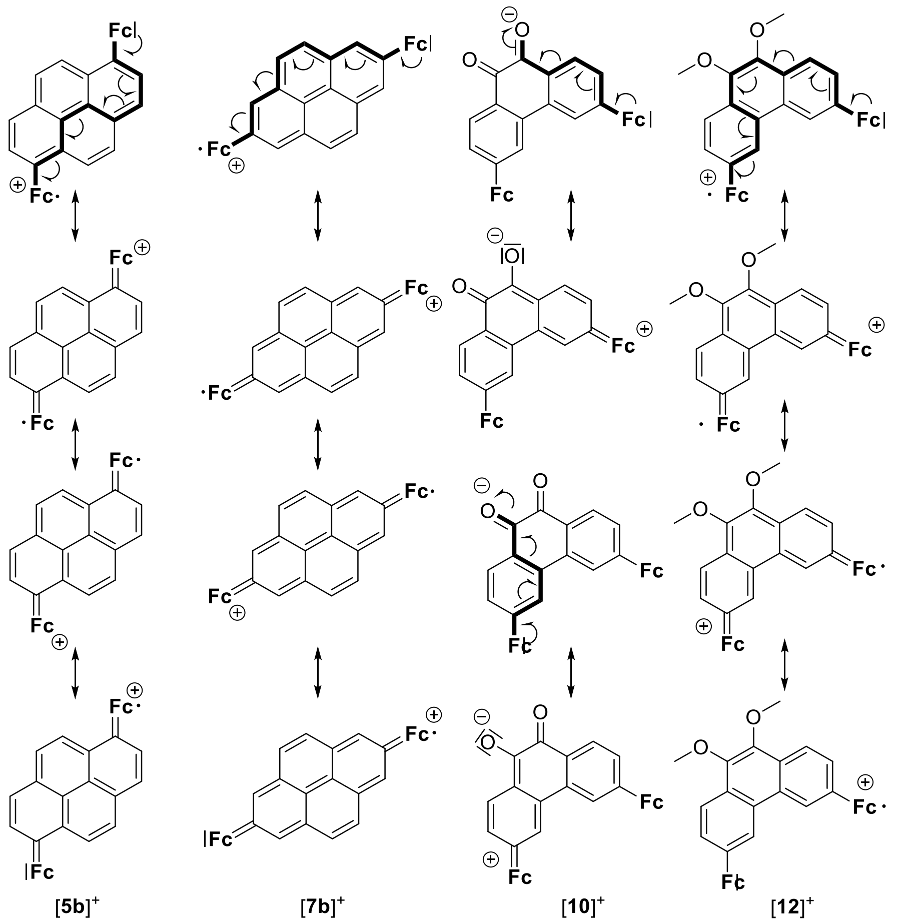

Scheme SI2. Resonance structures illustrating the through-bond charge transfer pathways in [**5b**]$^+$, [**7b**]$^+$, [**10**]$^+$ and [**12**]$^+$.

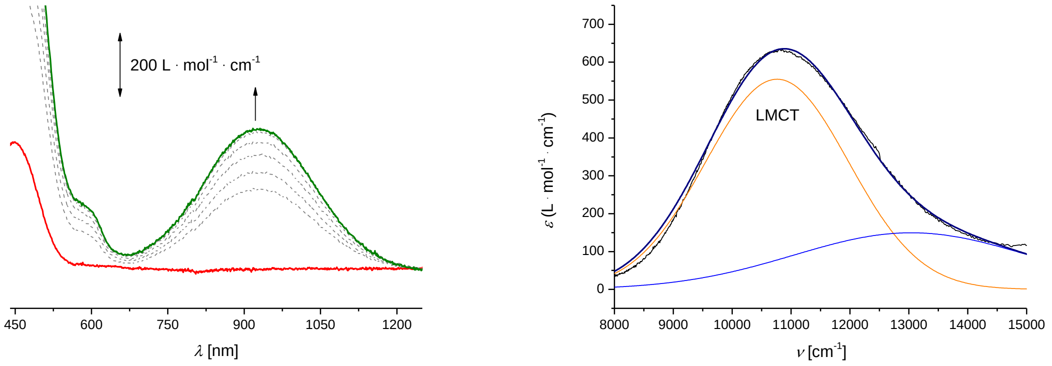

Figure SI17. Left: UV/vis/NIR spectra of **9-FcPhen** at 25 °C in dichloromethane (2.00 mmol · L^{-1}) at rising potentials (-200 to 800 mV *vs* Ag/Ag^+); supporting electrolyte $[N^nBu_4][B(C_6F_5)_4]$; arrows indicate the increasing and decreasing absorptions. Right: Deconvolution of the NIR absorption of [**9-FcPhen**]$^+$ using two Gaussian shaped bands.

Table SI8. NIR Data of the LMCT Absorptions of **9-FcPhen**.

Compd.	ν_{max} (cm^{-1})^a (ε_{max} ($L{\cdot}mol^{-1}{\cdot}cm^{-1}$))^b	$\Delta\nu_{1/2}$ (cm^{-1})^c
[**9-FcPhen**]$^+$	10765 (555)	2860

$^a\nu_{max}$ = Position of LMCT absorption. $^b\varepsilon_{max}$ = Intensity of the LMCT absorbtion. $^c\Delta\nu_{1/2}$ = Full width at half-height of LMCT absorption.

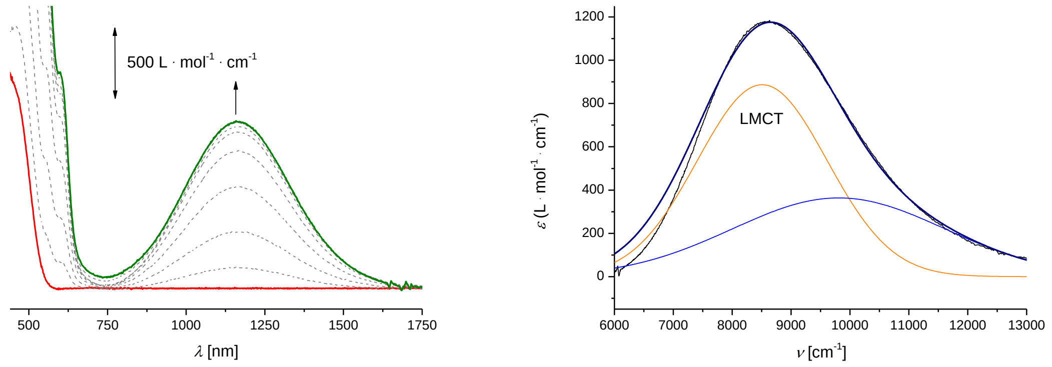

Figure SI18. Left: UV/vis/NIR spectra of **3** at 25 °C in dichloromethane (2.00 mmol · L^{-1}) at rising potentials (-200 to 600 mV *vs* Ag/Ag^+); supporting electrolyte $[N^nBu_4][B(C_6F_5)_4]$; arrows indicate the increasing and decreasing absorptions. Right: Deconvolution of the NIR absorption of [**3**]$^+$ using two Gaussian shaped bands.

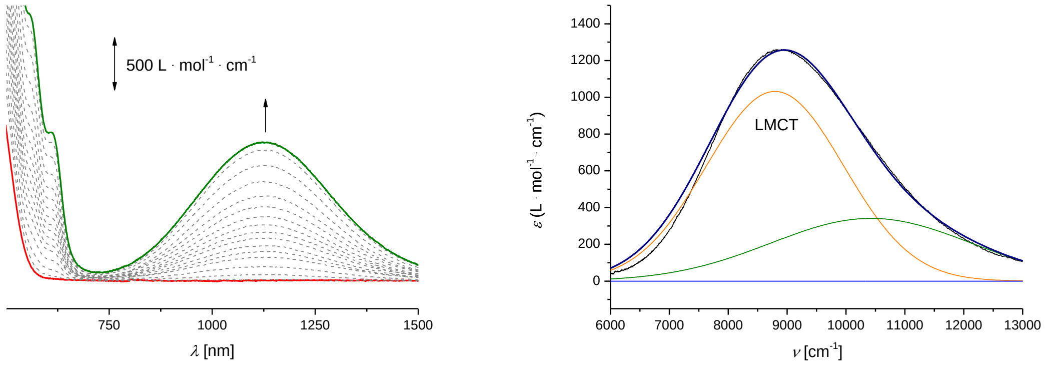

Figure SI19. Left: UV/vis/NIR spectra of **5a** at 25 °C in dichloromethane (2.00 mmol · L^{-1}) at rising potentials (-200 to 1400 mV *vs* Ag/Ag^+); supporting electrolyte [N^nBu_4][$B(C_6F_5)_4$]; arrows indicate the increasing and decreasing absorptions. Right: Deconvolution of the NIR absorption of [**5a**]$^+$ using two Gaussian shaped bands.

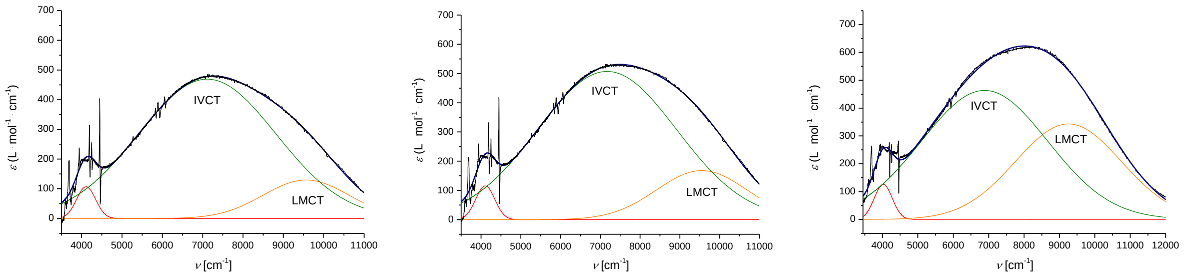

Figure SI20. Deconvolution of the NIR absorption of [**5b**]$^+$ at 560 mV (left), 575 mV (middle) and 590 mV (right) *vs* Ag/Ag^+ using three Gaussian shaped bands.

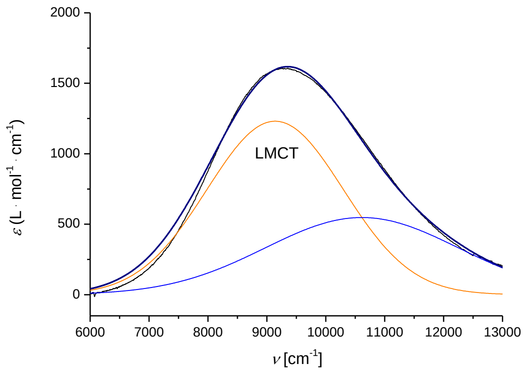

Figure SI21: Deconvolution of NIR absorption at 875 mV of [**5b**]$^{2+}$ using two Gaussian shaped bands.

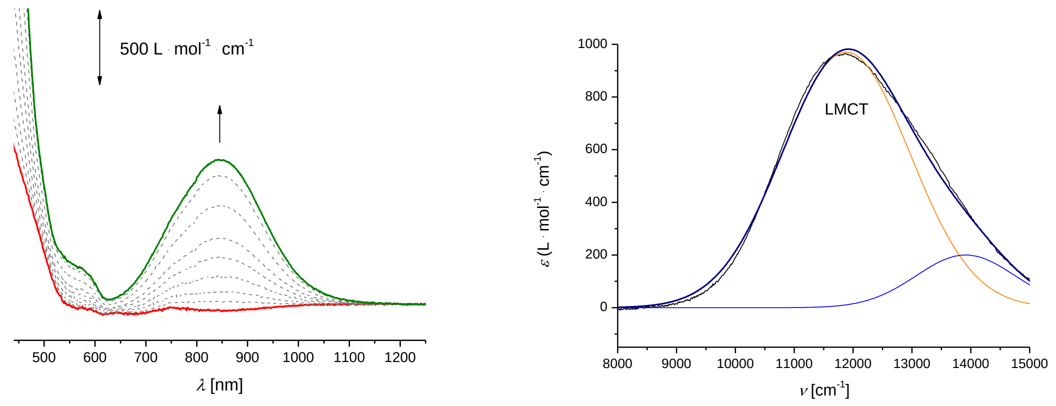

Figure SI22. Left: UV/vis/NIR spectra of **7a** at 25 °C in dichloromethane (2.00 mmol · L^{-1}) at rising potentials -00 to 425 mV *vs* Ag/Ag^{+}); supporting electrolyte [N^{n}Bu$_4$][B(C$_6$F$_5$)$_4$]; arrows indicate the increasing and decreasing absorptions. Right: Deconvolution of the NIR absorption of [**7a**]$^{+}$ using two Gaussian shaped bands.

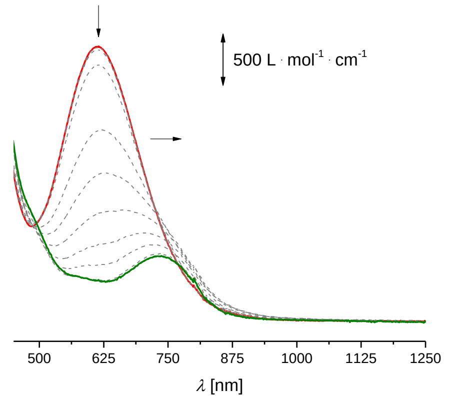

Figure SI23. Left: UV/vis/NIR spectra of **10** at 25 °C in dichloromethane (2.00 mmol · L^{-1}) at rising potentials (-200 to 675 mV *vs* Ag/Ag^{+}); supporting electrolyte [N^{n}Bu$_4$][B(C$_6$F$_5$)$_4$]; arrows indicate the increasing and decreasing absorptions.

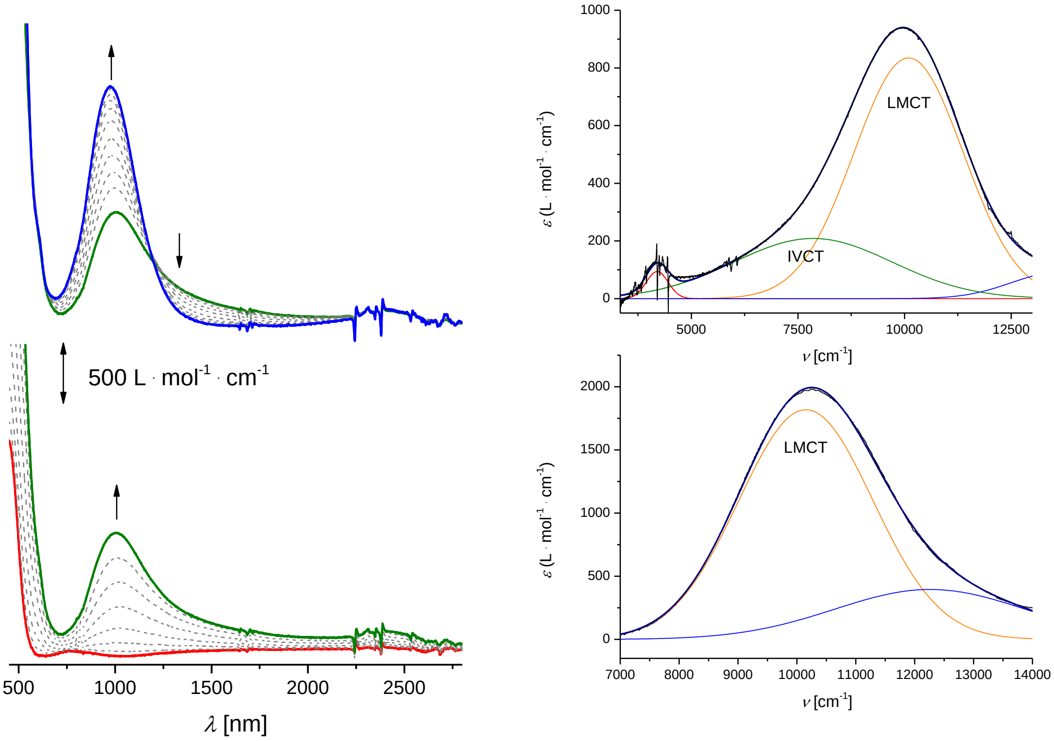

Figure SI24. Left: UV/vis/NIR spectra of **12** at 25 °C in dichloromethane (2.00 mmol · L^{-1}) at rising potentials (bottom: -200 to 350 mV; top: 350 to 650 mV *vs* Ag/Ag^+); supporting electrolyte [N^nBu_4][$B(C_6F_5)_4$]; arrows indicate the increasing and decreasing absorptions. Right, top: Deconvolution of the NIR absorption of [**12**]$^+$ using three Gaussian shaped bands. Right, bottom: Deconvolution of NIR absorption of [**12**]$^{2+}$ using two Gaussian shaped bands.

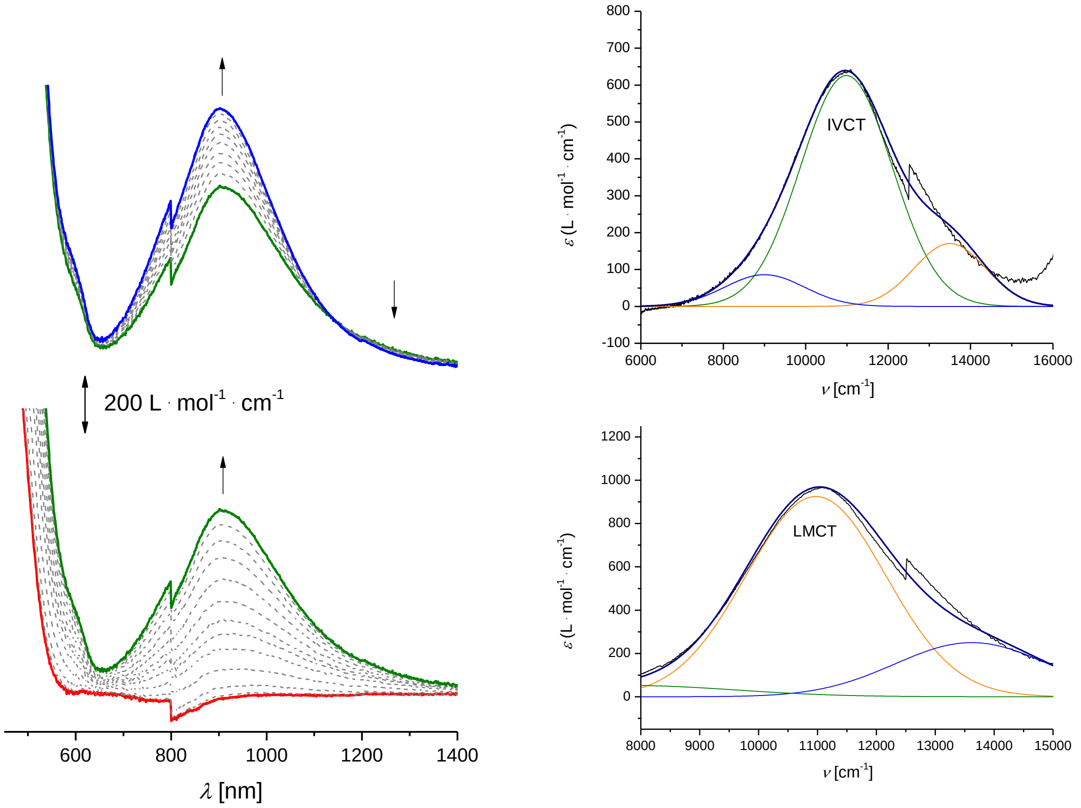

Figure SI25. Left: UV/vis/NIR spectra of **12** at 25 °C in propylene carbonate (2.00 mmol · L^{-1}) at rising potentials (bottom: -200 to 675 mV; top: 675 to 900 mV *vs* Ag/Ag^+); supporting electrolyte $[N^nBu_4][B(C_6F_5)_4]$; arrows indicate the increasing and decreasing absorptions. Right, top: Deconvolution of the NIR absorption of $[\mathbf{12}]^+$ using three Gaussian shaped bands. Right, bottom: Deconvolution of NIR absorption of $[\mathbf{12}]^{2+}$ using three Gaussian shaped bands.

Disentangling Experiments of chirality-enriched (*6*,*5*)-SWCNTs with 5b

In a typical study, a commercial SWCNT solid material was suspended in a chloroform solution of **5b** and treated with different sonication protocols (Experimental). The resulting suspension was centrifuged and the supernatant layer decanted and subjected to UV/vis/NIR analysis. The obtained UV/vis/NIR spectra are summarized in Figure SI26.

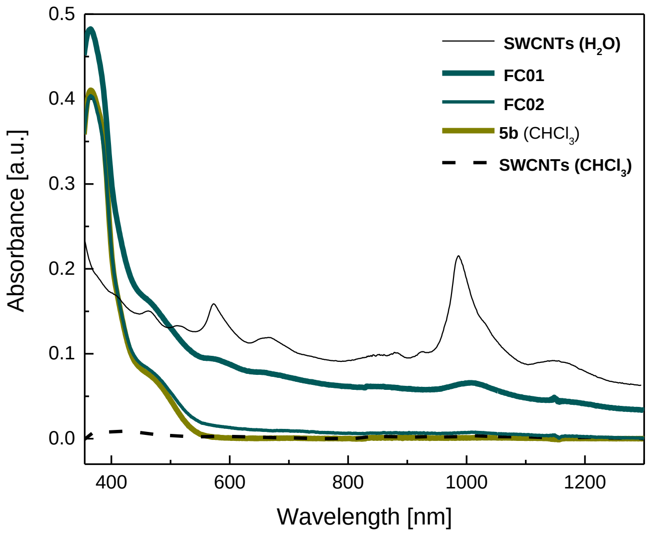

Figure SI26: UV/vis/NIR spectra of SWCNTs dispersed in chloroform in presence of **5b** as debundeling agent. Reference spectra of the same SWCNT batch in aqueous environment is given.

Figure SI26 shows the UV/vis/NIR spectra of the dispersions **FC01** and **FC02** together with the reference spectra **SWCNTs (H_2O)**, **5b** ($CHCl_3$) and **SWCNTs ($CHCl_3$)**. For the case of **FC01**, the *S11* transition of the *(6,5)*-SWCNTs is clearly seen at 1005 nm ($CHCl_3$), and this transition as well as the entire spectrum including the *S22* transitions in the range 390–710 nm images the reference spectrum of the SWCNTs in standard aqueous dispersants. Note that a solvatachromic shift of 5–15 nm between water and chloroform has to be taken into account and the spectra alike the raw material contain

quantities of SWCNTs of other chirality. From the absorbance ratio of the *S11* transition optical densities (**SWCNTs (H_2O)**: 0.12, **FC01**: 0.015) and the ratio of the solids contents (**SWCNTs (H_2O)**: 0.04 mg mL^{-1}, **FC01**: 0.125 mg mL^{-1}) we can assign that compound **5b** in chloroform shows a debundeling efficiency of 4 % compared to the one of an aqueous SWCNT dispersion achieved according to standard protocols[3–5] using a mixture of standard surfactants DOC:SDS 4:1 (DOC = sodium deoxycholate; SDS = sodium dodecyl sulfate). In contrast, UV/vis/NIR spectra of dispersion **FC02** shows neither the *S11* nor the *S22* transition compared to the reference spectrum of the SWCNTs in aqueous media. This finding indicates that the applied dispersion procedure with different sonication parameters does not lead to an effective disentangling of the SWCNTs in the presence of **5b**. The ability to effectively disentangle SWCNT solid material of **5b** can be explained by the pyrene moiety, which interacts *via* π-π interaction with the sidewalls of the SWCNTs.[6] Furthermore, the pyrene group is favorable instead using naphthyl- or phenyl-based compounds as reported for polycyclic aromatic ammonium salts.[7,8] The ability of **5b** to act as carbon-nanotube solubilizer is comparable to the results reported for dispersion of C_{70}-peapods (C_{70}@SWCNTs) by trimethyl-(2-oxo-2-pyrene-1-yl-ethyl)-ammonium bromide in water.[9]

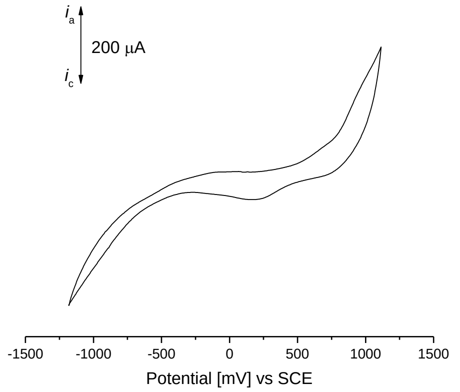

Figure SI27: Cyclic voltammogramm of **5b** (potential area -1250 to 1250 mV, scan rate 100 mV s^{-1}). Conditions: aqueous solution of KCl (1 mol L^{-1}) as supporting electrolyte, working electrode modified graphene paper.

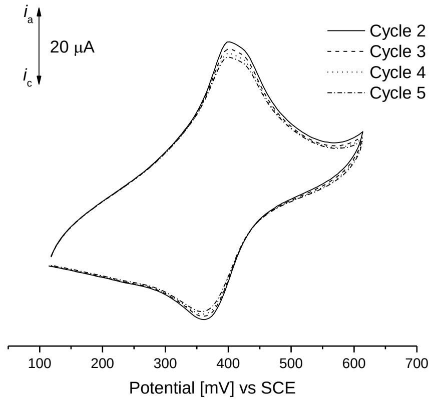

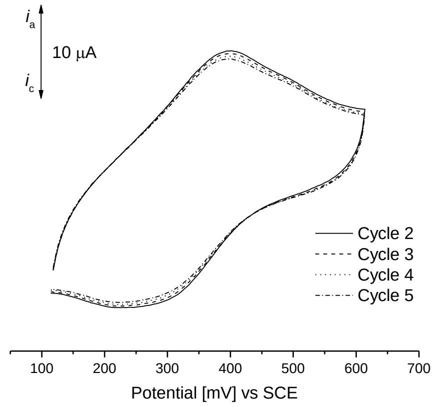

Figure SI28: Cyclic voltammogramms of cycles 2 to 5 of the ferrocenyl-based oxidation of **Gen2** (potential area: 100 to 600 mV) before (left) and after (right) the pyrene oxidation. Conditions: scan rate 100 mV s^{-1} in aqueous solution of KCl (1 mol L^{-1}) as supporting electrolyte, working electrode modified graphene paper.

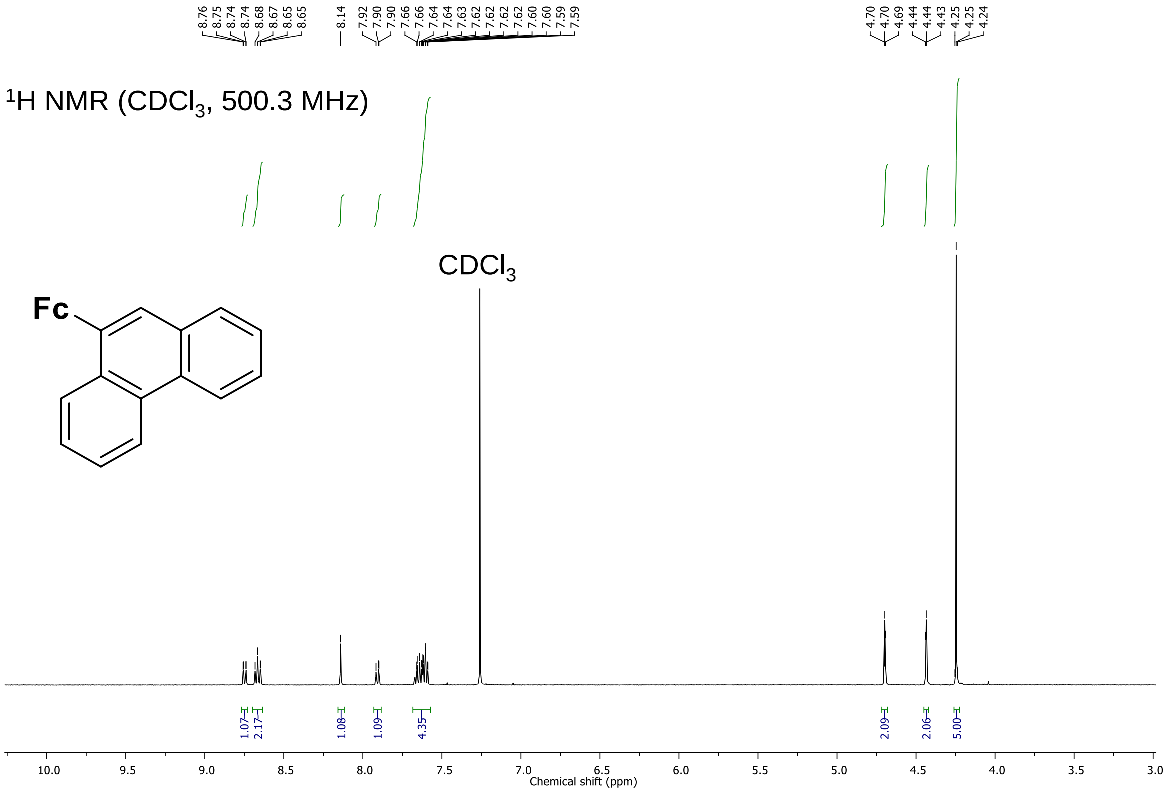

Figure SI29. ^{1}H NMR spectrum of **9-FcPhen** in $CDCl_3$.

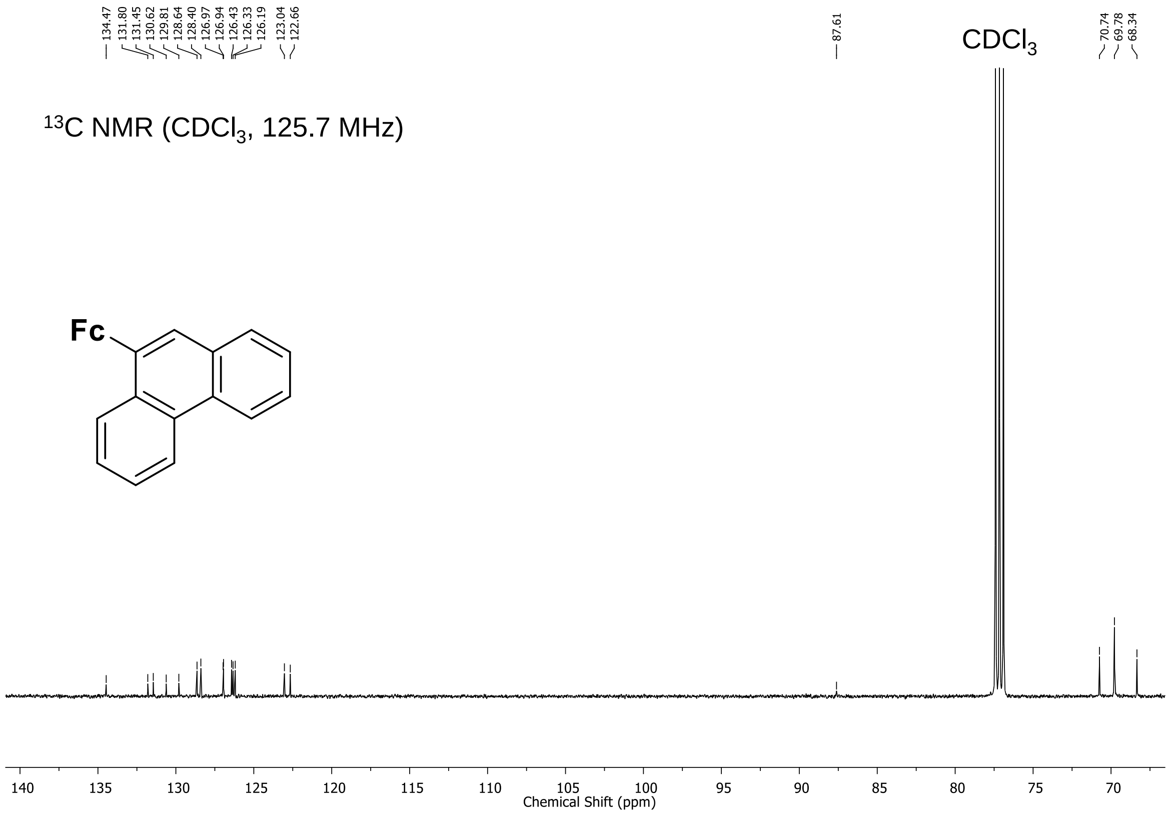

Figure SI30. ^{13}C NMR spectrum of **9-FcPhen** in $CDCl_3$.

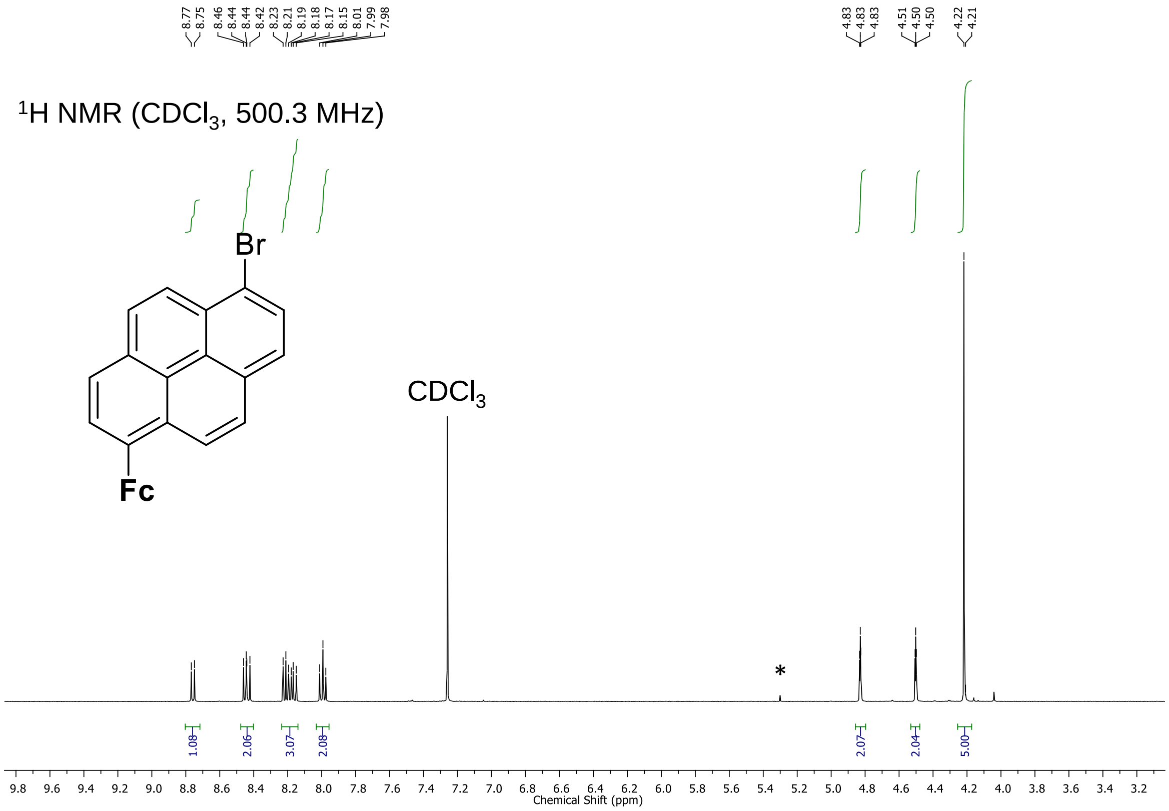

Figure SI31. ^{1}H NMR spectrum of **5a** in $CDCl_3$, *CH_2Cl_2.

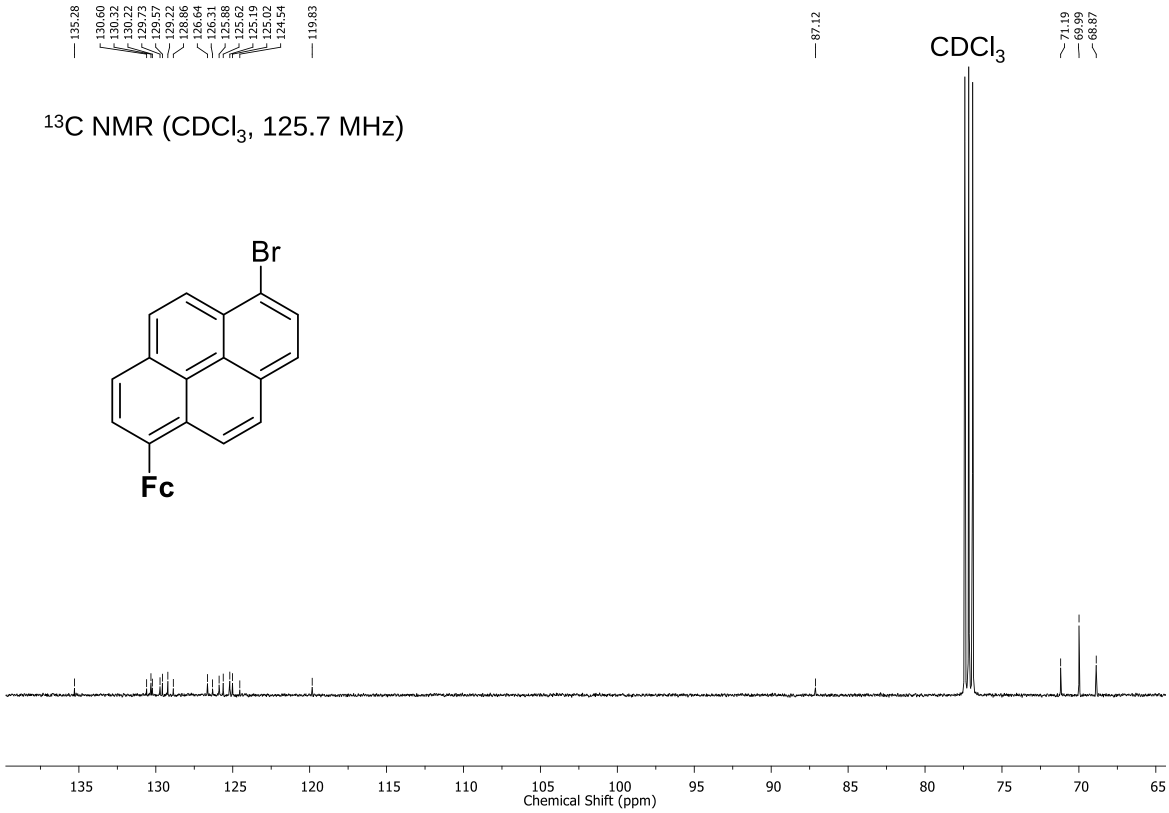

Figure SI32. ^{13}C NMR spectrum of **5a** in $CDCl_3$.

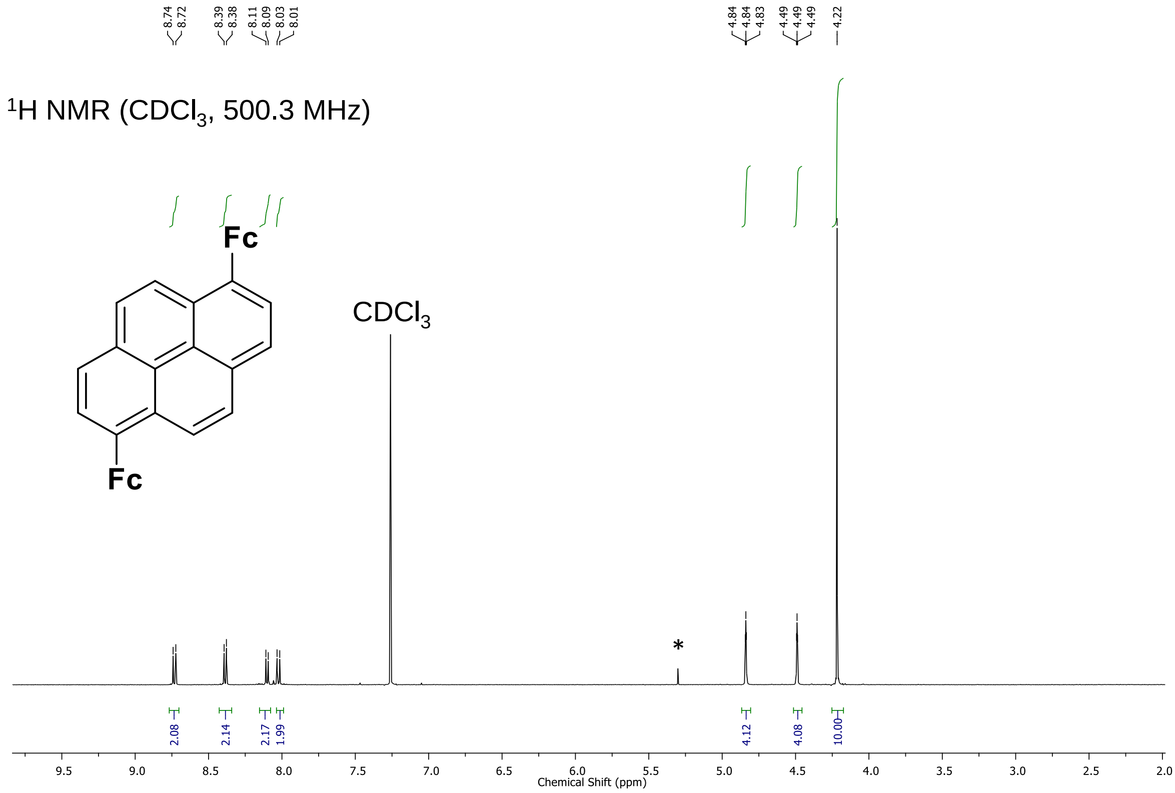

Figure SI33. ^{1}H NMR spectrum of **5b** in $CDCl_3$, $*CH_2Cl_2$.

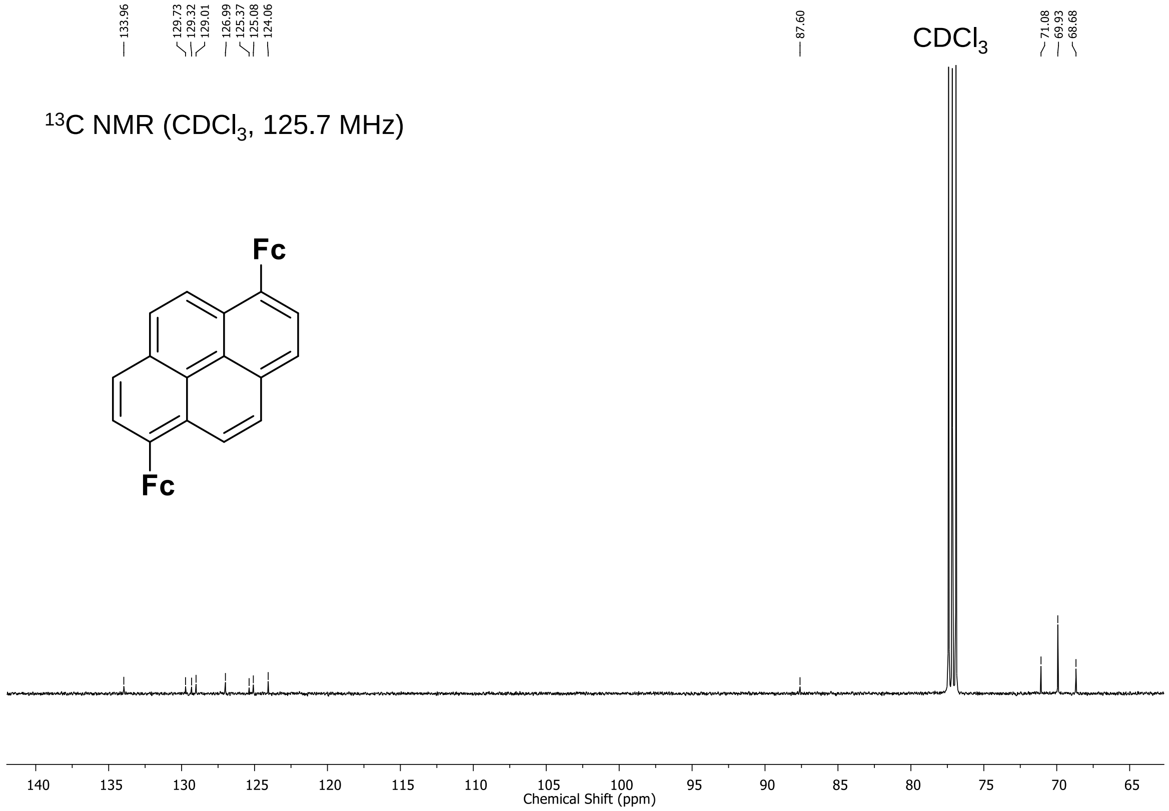

Figure SI34. ^{13}C NMR spectrum of **5b** in $CDCl_3$.

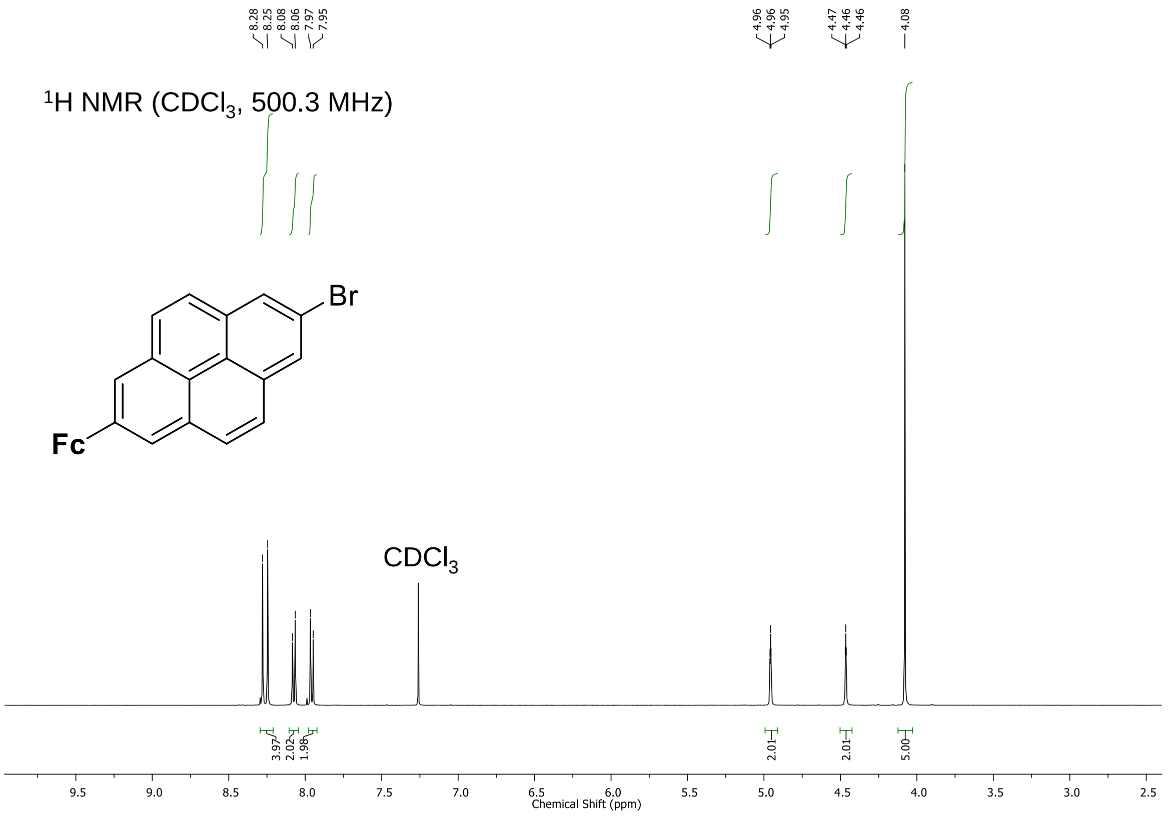

Figure SI35. ^{1}H NMR spectrum of **7a** in $CDCl_3$.

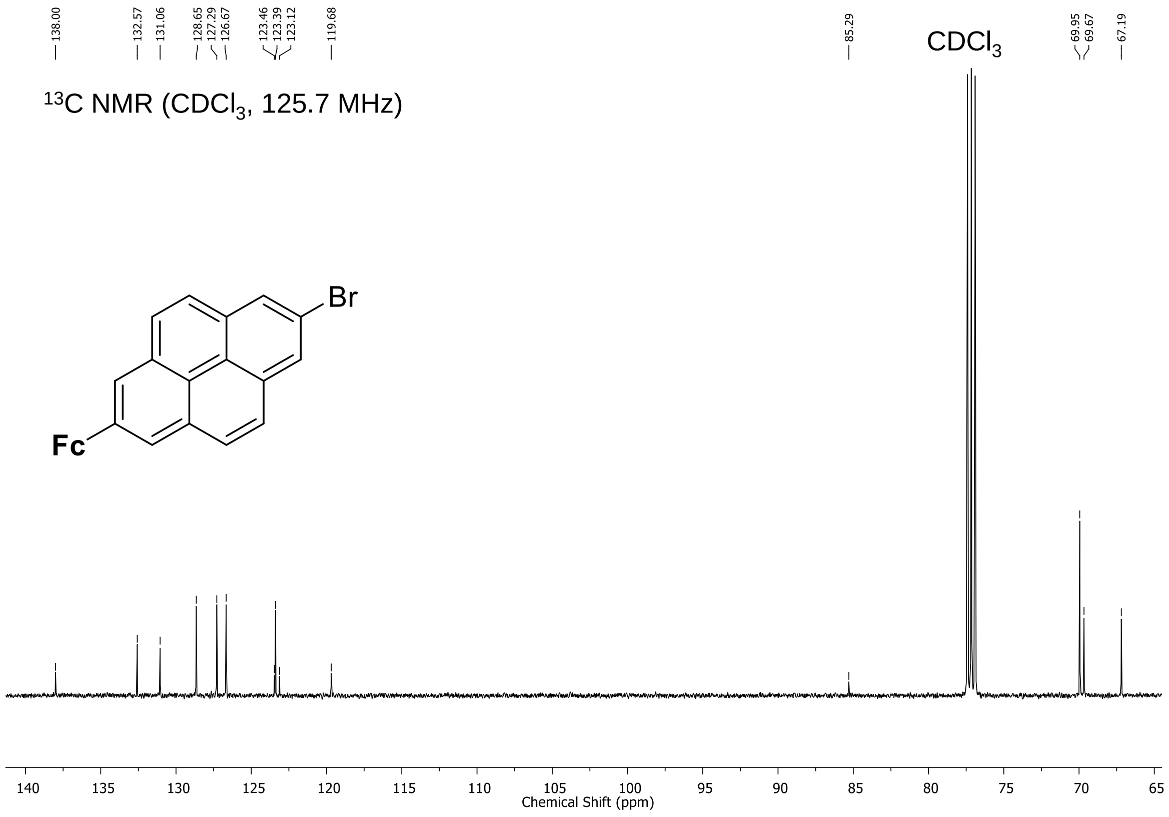

Figure SI36. ^{13}C NMR spectrum of **7a** in $CDCl_3$.

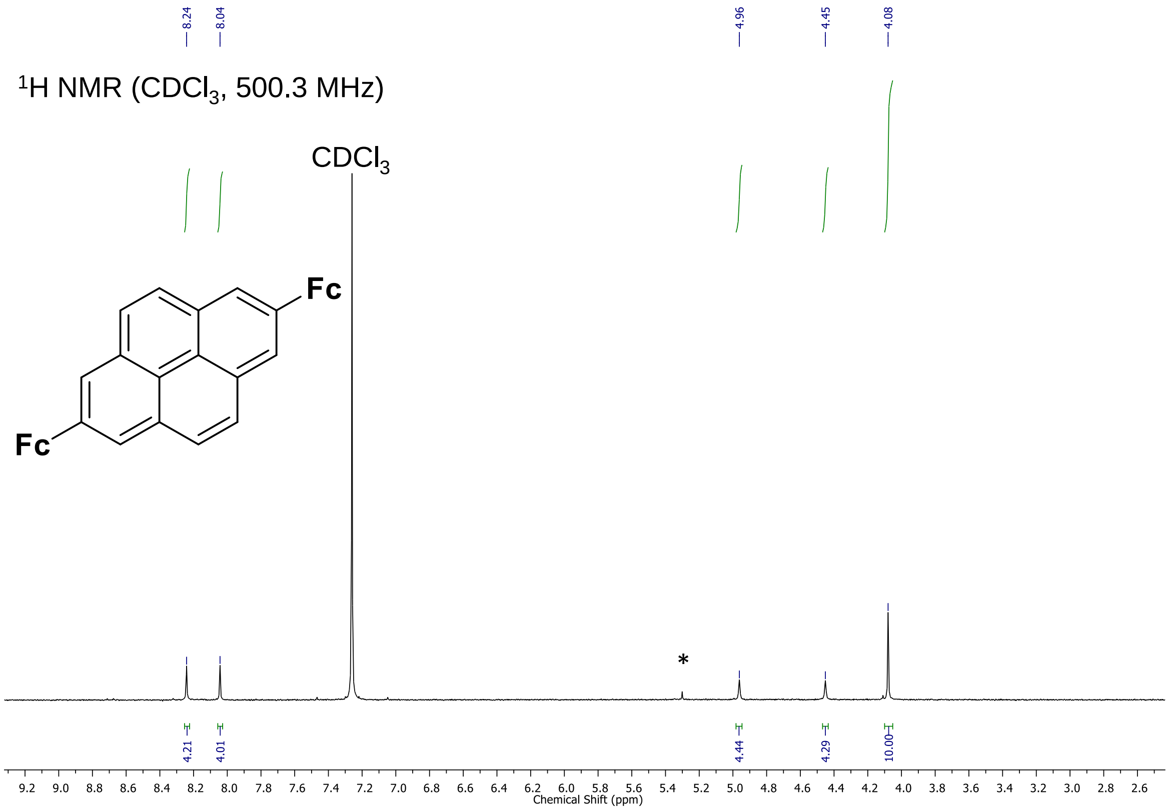

Figure SI37. ^{1}H NMR spectrum of **7b** in $CDCl_3$, $*CH_2Cl_2$.

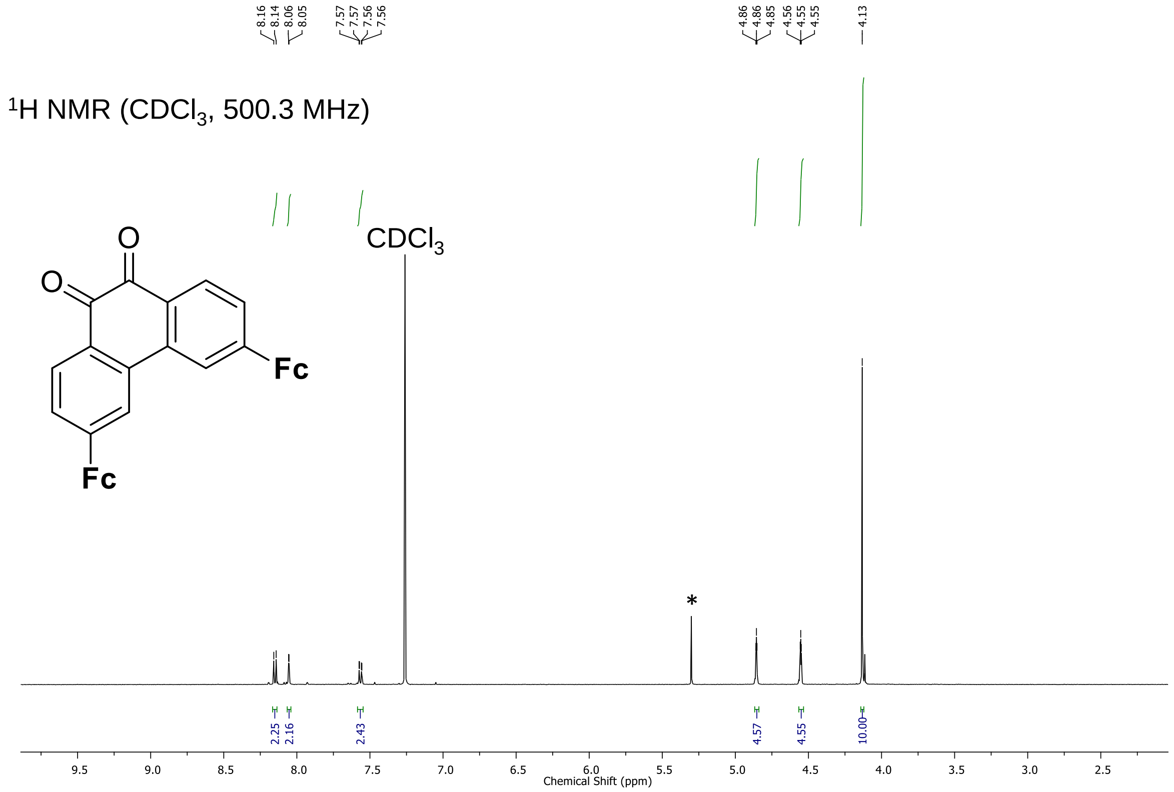

Figure SI38. ^{1}H NMR spectrum of **10** in $CDCl_3$, $*CH_2Cl_2$.

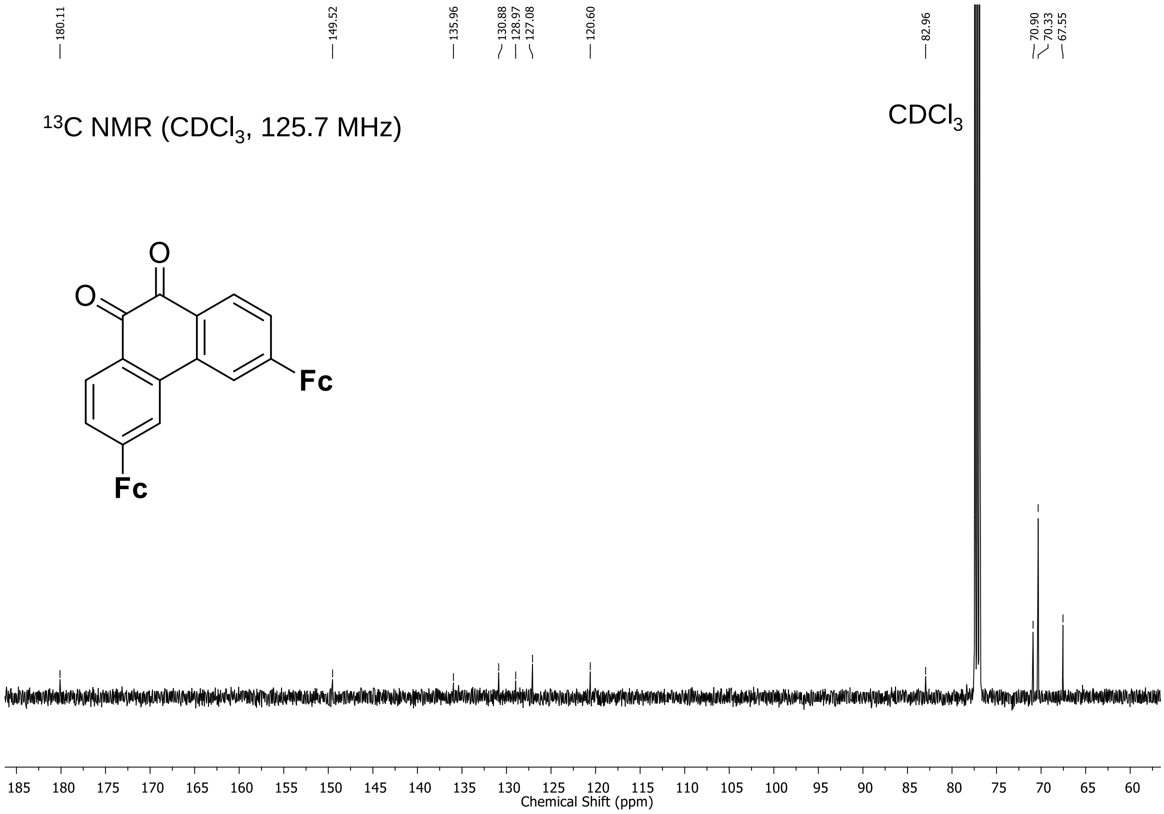

Figure SI39. ^{13}C NMR spectrum of **10** in $CDCl_3$.

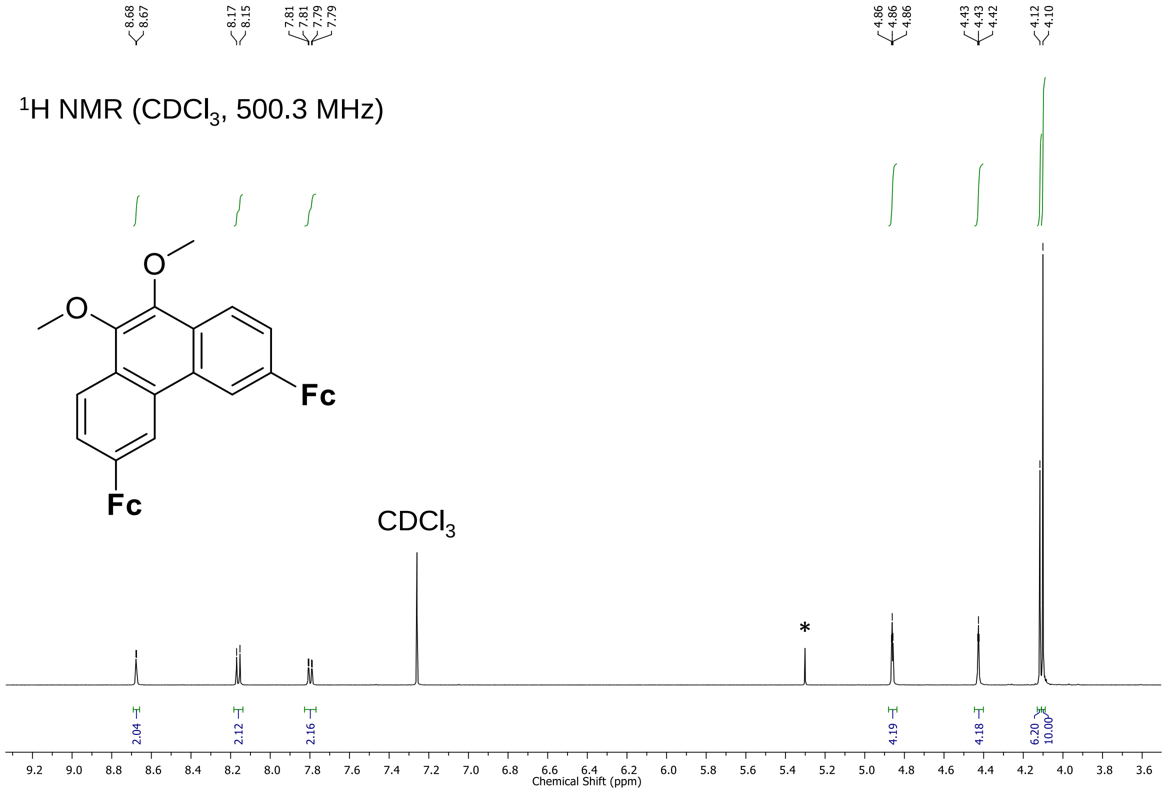

Figure SI40. ^{1}H NMR spectrum of **12** in $CDCl_3$, *CH_2Cl_2.

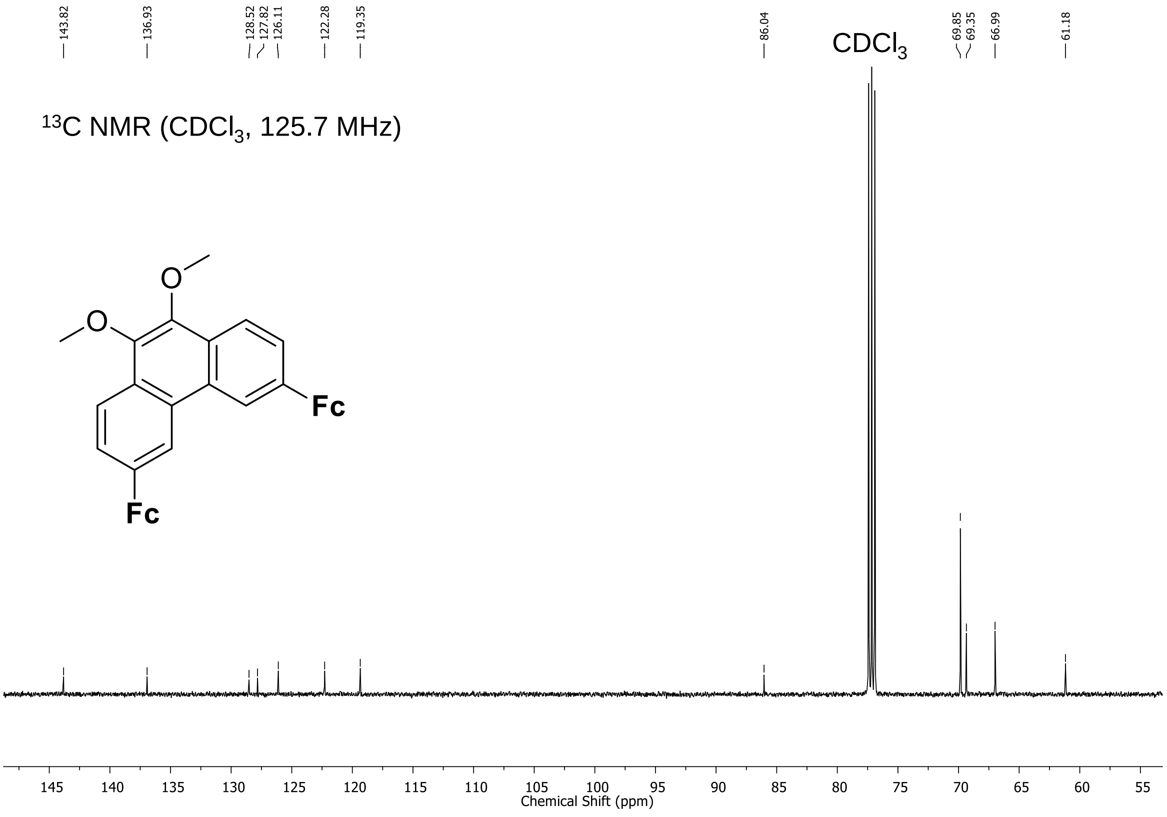

Figure SI41. ^{13}C NMR spectrum of **12** in $CDCl_3$.

References

[1] Y. Marcus, *Chem. Soc. Rev.* **1993**, *22*, 409–416.

[2] D. E. Richardson, H. Taube, *Inorg. Chem.* **1981**, *20*, 1278–1285.

[3] T. Blaudeck, D. Adner, S. Hermann, H. Lang, T. Gessner, S. E. Schulz, *Microelectron. Eng.* **2015**, *137*, 135–140.

[4] H. Yu, S. Hermann, S. E. Schulz, T. Gessner, Z. Dong, W. J. Li, *Chem. Phys.* **2012**, *408*, 11–16.

[5] S. Hartmann, T. Blaudeck, O. Hölck, S. Hermann, S. E. Schulz, T. Gessner, B. Wunderle, *J. Appl. Phys.* **2014**, *115*, DOI 10.1063/1.4870871.

[6] T. Fujigaya, N. Nakashima, *Polym. J.* **2008**, *40*, 577–589.

[7] Y. Tomonari, H. Murakami, N. Nakashima, *Chem. Eur. J.* **2006**, *12*, 4027–4034.

[8] N. Nakashima, Y. Tomonari, H. Murakami, *Chem. Lett.* **2002**, *31*, 638–639.

[9] N. Nakashima, Y. Tanaka, Y. Tomonari, H. Murakami, H. Kataura, T. Sakaue, K. Yoshikawa, *J. Phys. Chem. B* **2005**, *109*, 13076–13082.
